# A hidden pandemic? An umbrella review of global evidence on mental health in the time of COVID-19

**DOI:** 10.3389/fpsyt.2023.1107560

**Published:** 2023-03-08

**Authors:** Marlee Bower, Scarlett Smout, Amarina Donohoe-Bales, Siobhan O’Dean, Lily Teesson, Julia Boyle, Denise Lim, Andre Nguyen, Alison L. Calear, Philip J. Batterham, Kevin Gournay, Maree Teesson

**Affiliations:** ^1^The Matilda Centre for Research in Mental Health and Substance Use, Faculty of Medicine and Health, The University of Sydney, Sydney, NSW, Australia; ^2^Centre for Mental Health Research, The Australian National University, Canberra, ACT, Australia

**Keywords:** mental health, COVID-19, epidemiology, review, meta-review, umbrella review, psychopathology, public mental health

## Abstract

**Background:**

The mental health impacts of the COVID-19 pandemic remain a public health concern. High quality synthesis of extensive global literature is needed to quantify this impact and identify factors associated with adverse outcomes.

**Methods:**

We conducted a rigorous umbrella review with meta-review and present (a) pooled prevalence of probable depression, anxiety, stress, psychological distress, and post-traumatic stress, (b) standardised mean difference in probable depression and anxiety pre-versus-during the pandemic period, and (c) comprehensive narrative synthesis of factors associated with poorer outcomes. Databases searched included Scopus, Embase, PsycINFO, and MEDLINE dated to March 2022. Eligibility criteria included systematic reviews and/or meta-analyses, published post-November 2019, reporting data in English on mental health outcomes during the COVID-19 pandemic.

**Findings:**

Three hundred and thirty-eight systematic reviews were included, 158 of which incorporated meta-analyses. Meta-review prevalence of anxiety symptoms ranged from 24.4% (95%CI: 18–31%, *I*^2^: 99.98%) for general populations to 41.1% (95%CI: 23–61%, *I*^2^: 99.65%) in vulnerable populations. Prevalence of depressive symptoms ranged from 22.9% (95%CI: 17–30%, *I*^2^: 99.99%) for general populations to 32.5% (95%CI: 17–52%, *I*^2^: 99.35) in vulnerable populations. Prevalence of stress, psychological distress and PTSD/PTSS symptoms were 39.1% (95%CI: 34–44%; *I*^2^: 99.91%), 44.2% (95%CI: 32–58%; *I*^2^: 99.95%), and 18.8% (95%CI: 15–23%; *I*^2^: 99.87%), respectively. Meta-review comparing pre-COVID-19 to during COVID-19 prevalence of probable depression and probable anxiety revealed standard mean differences of 0.20 (95%CI = 0.07–0.33) and 0.29 (95%CI = 0.12–0.45), respectively.

**Conclusion:**

This is the first meta-review to synthesise the longitudinal mental health impacts of the pandemic. Findings show that probable depression and anxiety were significantly higher than pre-COVID-19, and provide some evidence that that adolescents, pregnant and postpartum people, and those hospitalised with COVID-19 experienced heightened adverse mental health. Policymakers can modify future pandemic responses accordingly to mitigate the impact of such measures on public mental health.

## Introduction

Severe acute respiratory syndrome coronavirus 2 (SARS-CoV-2 or ‘COVID-19’) – identified in November 2019 and declared a global pandemic in March 2020 – has resulted in unprecedented worldwide disruptions to human health and way-of-life for over 3 years (1). The scientific community have sought to quantify the impacts on mental health, with a proliferation of research examining both direct impacts, of COVID-19 infection, and indirect impacts, of infection-control measures, such as quarantine, social distancing, self-isolation and lockdowns, and broader socioeconomic disruptions (2–5).

In response to the rapid expansion of literature, many systematic reviews and meta-analyses have emerged to collate evidence on mental health during COVID-19, and in recent months, several umbrella reviews have been published to synthesise these systematic reviews and meta-analyses (6–8).

Existing umbrella reviews predominantly investigate the prevalence of mental disorders among healthcare workers (HCW) during the COVID-19 period, demonstrating high prevalence of mental ill-health among this group (6, 9–12). However, there are substantial gaps in the meta-review literature that must be addressed to advance our understanding of the global mental health impacts of the COVID-19 pandemic. Firstly, there is a paucity of meta-synthesis of the prevalence of mental disorders in populations other than HCW and children and adolescents (8). Secondly, no existing meta-reviews compare the prevalence of symptoms of mental ill-health during the pandemic period with pre-COVID-19 prevalence. Thirdly, only one other umbrella review identified had a search window past 2021 (8). Given the time taken for publication of original studies and reviews, this necessarily limits the scope of existing umbrella reviews to coverage early within the pandemic period. Therefore, a robust and updated synthesis of research on the mental health impacts of COVID-19 is critical to inform and improve time-sensitive clinical and policy responses to future pandemics (13).

This umbrella review aims to examine (1) the mental health impacts of the COVID-19 pandemic, including direct results of COVID-19 infection, and indirect consequences of infection-control measures and societal changes, and (2) the population groups and characteristics associated with greater risk of adverse mental health outcomes during COVID-19. In doing so, this review synthesises existing evidence to form a global picture of mental health during COVID-19 whilst also identifying influencing contextual factors.

## Methods

This umbrella review was registered *a priori* with PROSPERO (ID: CRD42020223778) and followed the Preferred Reporting Items for Systematic Reviews and Meta-Analyses (PRISMA) and British Medical Journal umbrella review methodological guidelines (14, 15).

### Search strategy and selection criteria

Scopus, Embase, PsycINFO, and MEDLINE were searched to identify any type of review reporting mental health outcomes during the COVID-19 pandemic. Keywords and MeSH terms related to ‘coronavirus disease’, ‘mental health’, and ‘review’ were included (see Supplementary Tables S1–S4 for the complete search strategy). The search window was from 17th November 2019 when COVID-19 was first identified to 29th March 2022. This marks over 2 years from the beginning of the COVID-19 pandemic, allowing for the publication of reviews investigating both the short and longer-term mental health impacts. All reviews that were not systematic reviews and/or meta-analyses were excluded during screening because of the high volume of review articles identified, to only include the highest level of research evidence.

To be included, reviews were required to be: (1) systematic reviews, meta-analyses, or combined systematic reviews and meta-analyses; (2) written in English; (3) published after 17th November, 2019 (first identified COVID-19 case); (4) published in a peer-reviewed journal; and (5) include data on mental illness and/or symptoms of mental illness and/or factors associated with mental illness in human subjects; (6) findings about the link between mental health and COVID-19 (including neuropsychiatric consequences of COVID-19 infection and indirect consequences of associated infection-control measures and societal changes); and (7) a search strategy. Reviews were excluded if they were: (1) primary research, abstracts, dissertations, or letters; (2) specialised medical and surgery-related articles; or (3) did not meet inclusion criteria above.

The emerging nature of COVID-19 led to uncertainty around the quantity of data that would be available if inclusion was limited to reviews covering mental disorders *only* and did not include factors and symptoms associated with mental disorders generally. As such, inclusion criteria five was intentionally broad. However, upon screening completion, we identified sufficient literature to exclude those reviews that only investigated concepts peripheral to mental illness (e.g., quality of life, sleep problems, burnout). Stress was included since reviews often examined acute stress, which is closely related to the Diagnostic Statistical Manual (Edition V) diagnosis of acute stress disorder (16). Although a departure from protocol, this decision enabled the focus of the paper to be more aligned to the review aims. This additional exclusion is indicated in the PRISMA diagram.

### Screening and data extraction

Search results were collated using EndNote X9 and uploaded to Covidence software for screening (17, 18). Review titles, abstracts and full-texts were independently screened by two authors. At every stage, discussion with third independent author resolved any screening conflicts. Eligible full-text articles were independently double-extracted using a Microsoft Excel (v.16). The extraction spreadsheet included: title; author; journal; publication year; type of review; outcomes of interest; populations of interest; search window; search strategy; number of included studies; quality appraisal instrument/s used and results; characteristics of reviewed primary studies (including countries; research settings; pooled n participants; study designs [cross-sectional, longitudinal, etc.]). For reviews that contained a meta-analysis, further information was extracted by outcome and population, including pooled participants (*n*); effect type/s; effect/s (with variance); publication bias; risk of bias; and heterogeneity statistics.

### Quality assessment

To assess quality and risk of bias, all reviews were assessed using the 16-item ‘A MeaSurement Tool to Assess Systematic Reviews (AMSTAR-2)’ (19, 20). Double-appraisal was performed independently by two authors and any conflicts were identified by a third author and then collectively resolved. Per the AMSTAR-2 protocol, for each of the 16 items, reviews could receive ‘yes’, ‘no’, ‘not applicable’, or in certain instances ‘partial yes’ rating. The AMSTAR-2 has a recommended grading procedure that specifies several items as critical: pre-registration of protocol (item 2); literature search adequacy (item 4); justification of excluded studies (item 7); risk of bias assessed (item 9); and risk of bias considered in interpretation of reviews (item 13). For meta-analyses two further items are critical: appropriateness of meta-analytical methods (item 11) and assessment of publication bias (item 15). A ‘no’ for any of these items is tallied as a ‘critical weakness’. A ‘no’ for the remaining items is tallied as a ‘non-critical weakness’. For the present review, criteria 7 was re-categorised as non-critical, as most reviews did not provide a list of excluded studies and – when considering the possible impact on study quality in conjunction with the pragmatic consideration of limitations on supplementary materials imposed by many journals – is unlikely to strongly impact review quality. To receive a rating of ‘high quality,’ reviews must have no critical flaws and less than two non-critical weaknesses; a ‘moderate quality’ rating requires no critical weakness, ‘low quality’ allows one critical weakness, and ‘critically low quality’ is allocated to reviews with more than one critical weakness.

In addition to the AMSTAR-2, three bespoke items were added to record whether reviews reported: (1) representativeness of samples included in original studies, (2) validity or reliability of measures included in original studies, and (3) time-period when original studies were conducted during the pandemic. These items were deemed to be relevant with respect to the aims of the present review and to provide an overview of gaps in reporting but were not incorporated into the overall quality appraisal grading.

Only reviews that received a moderate or higher AMSTAR-2 rating were included in the narrative synthesis to improve overall quality and confidence in findings. All meta-analyses, regardless of quality rating were included in the meta-review (unless excluded for other reasons, as specified in Supplementary Table S11), as the impact of quality could be quantitatively assessed and reported.

### Data synthesis

#### Narrative synthesis

Based on the Synthesis Without Meta-analysis (SWiM) guidelines (21) for quantitative data of intervention effects (see Supplementary Table S6 for completed SWiM checklist), a narrative synthesis was conducted to synthesise review findings that could not be included in the meta-review (including systematic reviews and meta-analyses that did not include adequate data to be included in the meta-review) and provide further context around data from meta-analyses that were included in the meta-review. We employed a quality-based approach to determine results reported in the narrative synthesis. Only reviews that had a ‘moderate’ or higher AMSTAR-2 quality rating (including risk of bias) were included in the narrative review, to mitigate the risk of including chance findings. This ensured only the most rigorous evidence were included. Of those reviews included in the narrative synthesis, our confidence in findings and making inferences around the impact on COVID-19 on mental health (from ‘very low’, ‘low’, ‘moderate’ to ‘high’) was based on the quality of evidence presented, including: assessed strength of evidence by review authors; risk of bias; between-study heterogeneity; consistency and preciseness of results (including directness of comparisons made); study design (e.g., cohort studies over correlational evidence); sample (e.g., more representative or larger samples); and, appropriate use of confounders.

As per the review protocol, the narrative synthesis was structured around the research aims, with the first section drawing on overall/general population COVID-19 impacts, including as a direct result of COVID-19 infection, and as an indirect consequence of infection-control measures and societal changes (1) and the second drawing on population groups associated with greater risk of adverse mental health outcomes (2).

Quantitative effects data were not transformed into standardised metrics, because in most cases insufficient information was provided in systematic reviews to achieve this. In order to establish the impacts of COVID-19 on mental health, the narrative review drew on several measures of effect, including (in order of inferential rigour): (1) standard mean difference (SMD) change over time from pre- and post-commencement of the pandemic or other relevant phenomena (e.g., lockdowns), using longitudinal studies or high-quality cross-sectional studies comparing equivalent measures in the same population; (2) the pooled prevalence or relative risk of probable mental disorder or symptoms; (3) quantitative meta-regressions or subgroup analyses examining the differential prevalence or effects in population groups, and (4) significant associations between population characteristics and mental health outcomes.

The narrative review synthesis was conducted by two authors. One author sorted extracted studies into the previously defined groups into the previously defined groups, and wrote a description of the relevant review (e.g., population/settings, phenomena, outcomes), along with an assessment of the certainty of the findings in relation to the review research questions. This analysis was cross-checked by a second author, who independently read eligible extracted studies, then reviewed the synthesis and conclusions based on this reading.

#### Meta-review: Statistical analysis

To determine the pooled prevalence of mental health conditions (outcomes), primary meta-analyses were pooled into a meta-review separately for each outcome using logit transformed proportions based on total sample *N* and *n* of events, with a back-transformation to obtain the pooled prevalence rates. Five mental health outcomes had enough data within included reviews to be pooled: probable anxiety, depression, stress, psychological distress, and post-traumatic stress disorder /symptoms (PTSD/PTSS). Additionally, due to the range of population-level estimates provided in primary reviews, subgroup meta-reviews were conducted to determine pooled prevalence rates of each mental health outcome at a population level. Meta-regression was used to investigate AMSTAR-2 grade as a potential moderator of pooled prevalence estimates. Analyses were performed using the ‘rma.glmm’ function for generalised linear mixed effects meta-analysis model in the R (version 4.2.1) package ‘metafor’ (22). *I*^2^ and Wald type Q statistic assessed between-review heterogeneity for each outcome model. *I*^2^ thresholds were interpreted as 50% reflecting moderate between-review heterogeneity, and 75% reflecting high between-review heterogeneity (23). The *p* values for the *Q* statistic indicated whether heterogeneity in umbrella review models was statistically significant (<0.05). Prediction intervals indicated the level of variability around the estimated pooled effect, and the certainty of a new study estimate (24). We also performed meta-regressions investigating AMSTAR-2 grade as a potential moderator of pooled prevalence estimates. *Q* and degrees of freedom are reported for these moderator tests [*Q*(df)].

Primary meta-analyses that combined COVID-19 and non-COVID-19 data, did not report a pooled population *N*, or did not provide relevant estimates were excluded from the meta-review of pooled prevalence (Supplementary Table S11).

To determine a pooled SMD in mental health outcomes compared to pre-pandemic levels, a meta-review of primary meta-analyses reporting changes in mental health was performed using the ‘rma’ function for random effects meta-analysis model with restricted maximum likelihood estimation. Primary reviews with effect sizes that were not reported as SMD (*K* = 2) were converted from risk ratios and odd ratios to SMD before meta-review.

Where an overall effect estimate was reported in addition to multiple subgroup effect estimates within a single review, extractors systematically coded the levels of data-portioning applied by the review. Where estimates were partitioned based on factors such as publication bias or influential cases, the overall estimate was used in the meta-review to maximise included data and apply a consistent approach across all reviews.

#### Meta-review: Population categorisation

To perform the subgroup analysis, population-level estimates provided by meta-analyses required categorisation to group comparable populations. Many reviews reported general population estimates and for the present review, COVID-19 survivors were also grouped with the general population, given that – at time of writing – over 600 million people worldwide were COVID-19 survivors (25) and reviews lacked detail about the severity or recency of infection, or the presence of Long-COVID symptoms. Conversely, COVID-19 patients were retained as a unique population, as these individuals had COVID-19 at time of measurement. Estimates among children, adolescents, or students (other than healthcare students) were grouped as ‘young people.’ Healthcare students were grouped with healthcare workers (‘HCW’) due to original studies often being conducted while HCW students were on placement in hospitals performing duties similar to those of HCW. Estimates among people at any stage of pregnancy were re-categorised to ‘pregnant and postpartum people.’ Finally, vulnerable populations were grouped (elderly, people with chronic conditions, people hospitalised for non-COVID-19 reasons). There were several reviews that provided estimates for a ‘mixed’ population group that combined disparate populations into one group (and did not report estimates by population), and there was one review that contained quarantined individuals and one review that contained only elderly individuals in different sub-populations (26). These reviews were not included within the main meta-review models due to the lack of suitability for sub-population models. However, they were included within supporting information (Supplementary Table S13).

#### Original study overlap: Corrected covered area analysis

Corrected covered area (CCA) analysis was used to examine non-independence or ‘overlap’ of the original studies included across meta-analyses prior to performing the meta-review. Overlap occurs when a primary study is included in multiple meta-analyses and these meta-analyses are then pooled in a meta-review, which can introduce a particular study to bias results through duplication of findings (27).

Hennessy and Johnson’s CCA procedure (27), as an extension to the standard procedure developed by Pieper et al. (28), was used. The CCA methodology provides an adjusted proportion of overlap (0–5% indicates low overlap, 6–10% moderate overlap, 11–15% high overlap and over 15% very high overlap). The CCA analysis is performed multiple times to examine the overlap between reviews included in each estimate (e.g., overall estimate and subgroup estimates).

To perform CCA analysis, citations of included primary studies in meta-analyses were extracted into a Microsoft Excel (v16) spreadsheet ‘matrix’ that facilitated calculations of overlap across reviews. This matrix was then duplicated for every meta-reviewed outcome (depression, anxiety, stress, psychological distress, and PTSD/PTSS) so that each meta-reviewed outcome had a dedicated matrix of citations that were included. These five matrixes were then further replicated – and the citations further narrowed – to produce one matrix for each population group within each outcome for which a pooled prevalence was to be generated (i.e., depression in HCW, depression in general population, etc.) The full spreadsheet of matrixes is available as a Supplementary File. Each of these matrixes were then used to perform the CCA calculations to identify the amount of overlap for that particular outcome and for each population within that outcome (27).

Each matrix was checked for any instances where two reviews had a complete overlap of primary studies, which did not occur. Had this occurred, the Hennessy and Johnson method provides guidance on selecting which meta-analysis to include (27). There was one review that did not list the full references of included studies (29). Review authors were contacted but did not respond, so this review was excluded from CCA analysis.

## Results

### Study characteristics and quality appraisal

**Figure 1:**
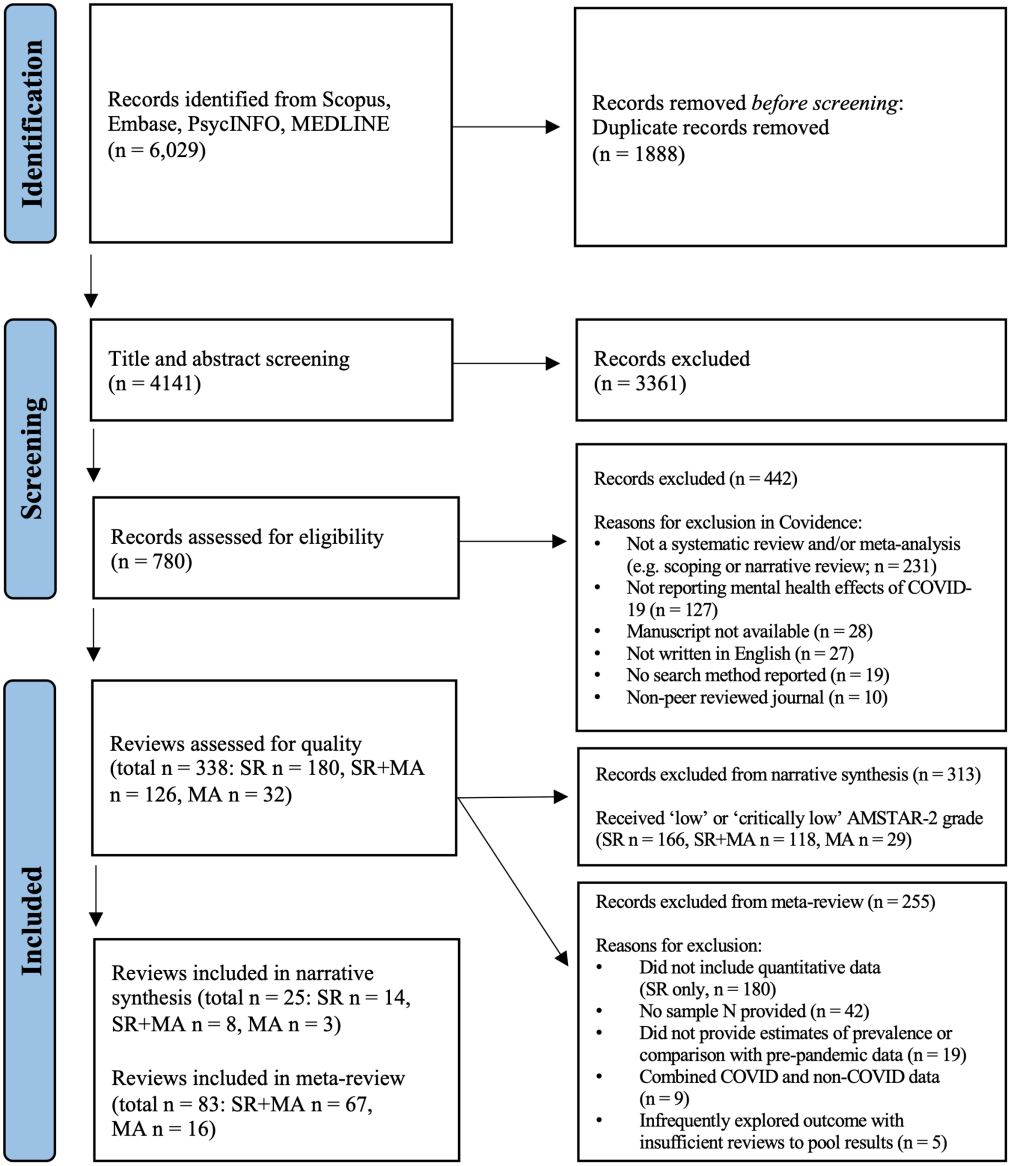
PRISMA diagram. PRISMA flow diagram outlining the progression of the 5-phase systematic search undertaken to retrieve articles examining the impact of COVID-19 on mental health outcomes. Phase 1: The initial search across four databases (Scopus, Embase, PsycINFO, MEDLINE) yielded 6,029 records, of which 1,888 articles were identified as duplicates, and removed before screening commenced. Phase 2: The titles and abstracts of 4,141 records were screened for relevance, which led to 3,361 records being excluded. Phase 3: Subsequently, 780 articles underwent full-text screening, with 442 records removed prior to quality appraisal. Phase 4: Of the 338 reviews assessed for quality using the AMSTAR-2 critical appraisal tool, 313 records were excluded from the narrative synthesis, and 255 records were excluded from the meta-review. Phase 5: Twenty-five records were included in the narrative synthesis, comprising 14 systematic reviews, 8 combined systematic reviews and meta-analyses, and 3 meta-analyses. There were 84 records included in the meta-review consisting of 67 combined systematic reviews and meta-analyses and 16 meta-analyses. *n*, number of records; SR, systematic review; MA, meta-analysis; SR + MA, systematic review and meta-analysis; AMSTAR-2, Assessment of Multiple Systematic Reviews Revised; PRISMA, Preferred Reporting Items for Systematic Reviews and Meta-Analyses.

Per the PRISMA diagram (Figure 1), 6,029 citations were identified through searches. Post-screening, 338 reviews were eligible for inclusion, comprising 180 systematic reviews, 32 meta-analyses, and 126 systematic reviews with meta-analysis.

All 338 full-text reviews were appraised using the AMSTAR-2 criteria, with 236 ‘critically-low’, 77 ‘low’, 25 ‘moderate’ and 0 ‘high’ quality reviews. Applying the three bespoke items across the 338 reviews: 51% reported the degree of representativeness of samples included in original studies; 57% reported information about the validity and/or reliability of measures included in original studies; and only 26% reported the time-period original studies were conducted during the pandemic (Figure 1).

#### Reviews included in narrative synthesis

All 25 reviews rated as moderate quality on the AMSTAR-2 (*K* = 14 systematic reviews and *K* = 11 reviews with meta-analysis) were included in the narrative synthesis (30–42) (see Supplementary Table S8 for detailed review characteristics). The total number of participants was 6,008,573 (two reviews did not report participant numbers). HCW were the most common population group reported (*K* = 8), followed by the general population (*K* = 5). Other populations included young people (children, adolescents or college students, *K* = 5), current COVID-19 patients (*K* = 2), people with previous COVID-19 infection (*K* = 2), pregnant or postpartum people and mothers of young children (*K* = 2). The remaining reviews (*K* = 3) did not examine a specific population. One review (43) limited inclusion to studies with samples from China, whereas all others included studies with samples from minimum three different countries (Supplementary Table S8). Reviews excluded from narrative synthesis due to low-quality are presented in Supplementary Table S7.

#### Reviews included in meta-reviews of pooled prevalence and standard mean difference

After excluding meta-analyses failing to provide adequate data required for meta-review, 83 meta-analyses were included in the meta-reviews (see Supplementary Table S11 for excluded studies). All 83 reviews were included regardless of AMSTAR-2 rating, as the impact of review quality could be quantitatively examined as a moderating variable (Supplementary Table S9). Seventy-six meta-analyses were included in the meta-review of pooled prevalence (total pooled sample size 10,983,831). The most analysed probable outcomes were depression (*k* = 70), anxiety (*k* = 61), stress (*k* = 18), post-traumatic stress disorder (PTSD, *k* = 24) and psychological distress (*k* = 5). As with the reviews included in the narrative synthesis, HCW, general population and young people were the most examined groups. Eight meta-analyses were included in the meta-review of pooled standard mean difference comparing during-COVID-19 estimates to pre-COVID-19 estimates; these reviews provided sufficient estimates to pool findings for depression (*k* = 9) and anxiety (*k* = 8). Remaining estimates for other mental health conditions were also pooled (*k* = 7). Supplementary Table S10 details the countries of original studies in each review included in the meta-review.

### Study duplication and corrected covered area analysis

CCA analysis revealed low overlap (<5%) for the overall estimates for all five outcomes (Supplementary Table S5). For depression, a total of 1,393 original studies were included across all reviews, 1,230 were included for anxiety, 728 for stress, 191 for psychological distress and 632 for PTSD/PTSS. Overlap was low-to-moderate for all population groups aside from depression and anxiety for pregnant and postpartum people, and for stress among young people, for which the overlap was moderate-to-high. Pooled analysis against these three groups should be interpreted with caution given potential bias introduced by non-independence of observations.

### Meta-review: Pooled prevalence

Table 1 displays probable pooled prevalence and heterogeneity statistics for all meta-review models.

**Table 1:** Meta-review model summary and heterogeneity statistics for all models of probable depression, anxiety, stress, psychological distress, and post-traumatic stress disorder during the COVID-19 pandemic, including pooled prevalence and standardised mean difference.

Prevalence during COVID-19 pandemic period (pooled prevalence)							
	k (N)	Pooled prevalence	95% CI	Prediction interval	*Tau*^2^	*I*^2^	QE
**Depression**							
Overall	70	0.2809	0.26, 0.30	0.13, 0.51	0.24	99.97%	96139.40 (<0.001)
General population	15 (2330112)	0.2288	0.17, 0.30	0.07, 0.54	0.47	99.99%	42666.29 (<0.001)
Healthcare workers	27 (1271283)	0.2916	0.26, 0.32	0.16, 0.46	0.14	99.92%	19685.18 (<0.001)
COVID-19 patients	5 (56742)	0.3159	0.22, 0.43	0.13, 0.60	0.29	99.72%	1521.05 (<0.001)
Vuln. populations	3 (8039)	0.3246	0.17, 0.52	0.08, 0.72	0.53	99.35%	286.29 (<0.001)
Young people	11(1889497)	0.3210	0.28, 0.36	0.21, 0.46	0.08	99.95%	8796.25 (<0.001)
Pregnant/postpartum	9 (134353)	0.2620	0.24, 0.29	0.19, 0.35	0.04	99.16%	455.32 (<0.001)
**Anxiety**							
Overall	61	0.3075	0.28, 0.33	0.14, 0.54	0.24	99.97%	80317.56 (<0.001)
General population	13 (1134349)	0.2435	0.18, 0.31	0.08, 0.55	0.42	99.98%	22350.45 (<0.001)
Healthcare workers	24 (1130882)	0.3141	0.29, 0.34	0.19, 0.47	0.11	99.91%	21648.02 (<0.001)
COVID-19 patients	5 (54613)	0.3212	0.22, 0.44	0.12, 0.63	0.35	99.73%	2976.61 (<0.001)
Vuln. populations	3 (9843)	0.4109	0.23, 0.61	0.12, 0.78	0.53	99.65%	599.01 (<0.001)
Young people	10 (1872049)	0.3159	0.28, 0.35	0.22, 0.43	0.06	99.94%	12655.74 (<0.001)
Pregnant/postpartum	6 (93838)	0.3597	0.33, 0.39	0.28, 0.45	0.03	98.98%	640.38 (<0.001)
**Stress**							
Overall	18	0.3910	0.34, 0.44	0.21, 0.60	0.19	99.91%	4910.25 (<0.001)
General population	4 (240146)	0.3819	0.32, 0.45	0.25, 0.53	0.08	99.89%	1229.26 (<0.001)
Healthcare workers	11 (239588)	0.4087	0.36, 0.46	0.24, 0.60	0.14	99.85%	2906.40 (<0.001)
Pregnant/postpartum	1 (1765)	NA – Only one review					
Young people	2 (2537)	0.2509	0.23, 0.27	0.23, 0.28	0.00	5.08%	2.50 (*p* = 0.11)
**Psychological distress**							
Overall	5	0.4417	0.32, 0.58	0.17, 0.75	0.38	99.95%	5439.91 (<0.001)
General population	2 (138343)	0.4019	0.28, 0.54	0.21, 0.64	0.16	99.96%	4813.59 (<0.001)
Healthcare workers	2 (16954)	0.3536	0.28, 0.44	0.23, 0.50	0.06	99.10%	222.19 (<0.001)
Pregnant/postpartum	1 (705)	NA – Only one review					
**Post-traumatic stress disorder**							
Overall	24	0.1877	0.15, 0.23	0.07, 0.43	0.36	99.87%	5960.67 (<0.001)
General population	7 (217218)	0.1384	0.10, 0.20	0.05, 0.35	0.33	99.92%	2268.11 (<0.001)
Healthcare workers	12 (130517)	0.2014	0.16, 0.26	0.08, 0.43	0.29	99.78%	2420.20 (<0.001)
COVID-19 patients	4 (6216)	0.2255	0.14, 0.33	0.08, 0.49	0.30	98.52%	458.43 (<0.001)
Young people	1 (4242)	0.3210	0.28, 0.36	0.21, 0.46	0.08	99.95%	8796.25 (<0.001)
**Comparison to pre-pandemic (pooled standard mean difference)**							
**Outcome**	**k**	**SMD**	**95% CI**	**Prediction interval**	**Tau**^**2**^	***I***^**2**^	**QE**
Depression	9	0.2	0.07, 0.33	−0.09, 0.49	0.018	73.21%	22.41 (*p* < 0.01)
Anxiety	8	0.2866	0.12, 0.45	−0.13, 0.70	0.038	80.59%	26.76 (*p* < 0.001)
Other	7	0.0965	-0.07, 0.26	−0.29, 0.48	0.031	80.58%	24.97 (*p* < 0.001)

This Table 1 provides a summary of pooled prevalence and heterogeneity statistics for meta-review models across five mental health outcomes (probable depression, anxiety, stress, psychological distress, and PTSD/PTSS) across the overall sample, and the six population subgroups (general population, healthcare workers (HCW), COVID-19 patients, pregnant/postpartum individuals, vulnerable populations, and young people). It also provides a summary of pooled standardised mean difference (SMD) and heterogeneity statistics for meta-review models that reported an effect of COVID-19 compared to pre-pandemic for three mental health outcomes (probable depression, anxiety, and other mental health conditions) across the overall sample. K, number of studies; N, number of participants; 95%CI, 95% confidence interval; Tau^2^, estimated amount of total heterogeneity, *I*^2^, total heterogeneity/total variability; *H*^2^, total variability/sampling variability; QE, quantile estimation; SMD, pooled standardised mean difference; PTSD, post-traumatic stress disorder/post-traumatic stress symptoms.

**Figure 2:**
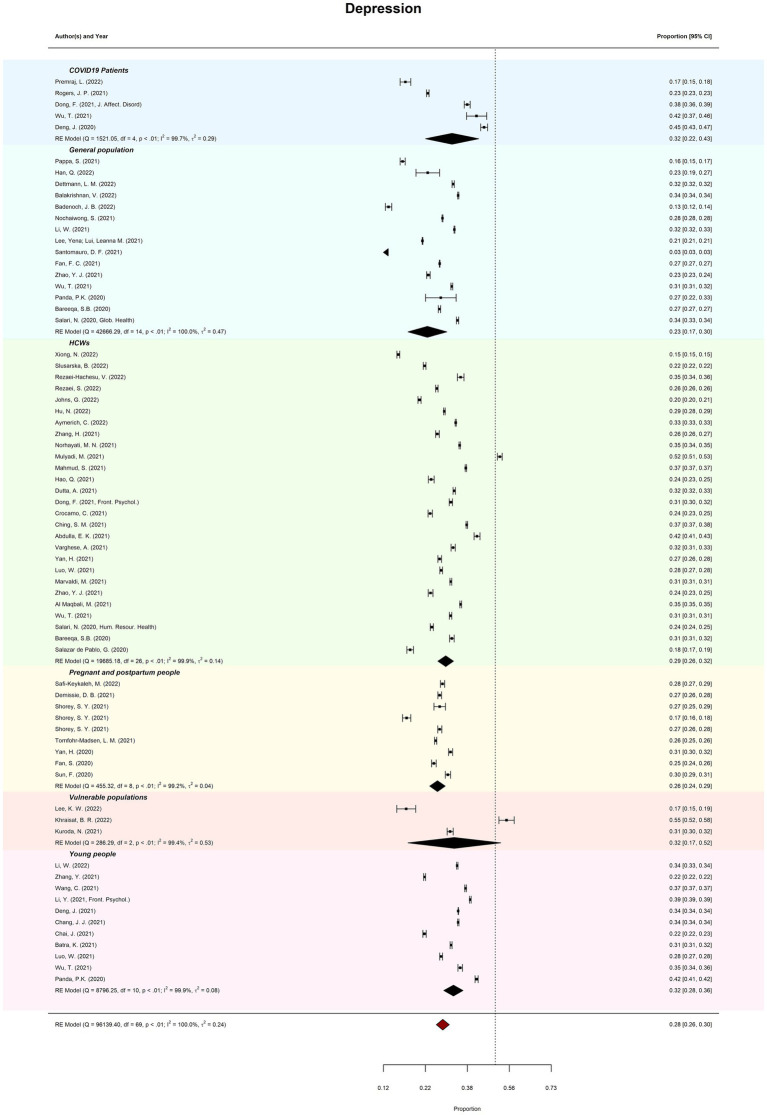
Meta-review of pooled prevalence of depression. Displays the forest plots of probable depression prevalence (and 95% CIs) across all subgroups.

Meta-review of overall probable depression prevalence (*k* = 70) was 28.09% (95%CI = 26–30%; PI = 13–51%). There was considerable between-review heterogeneity (*I*^2^ = 99.97%; *Tau*^2^ = 0.24; QE(69) = 96139.40, *p* < 0.001). Subgroup analysis revealed that prevalence of depression ranged from 22.88% (general populations) to 32.46% (vulnerable populations). Meta-regression moderation analysis indicated no significant difference in prevalence estimates by review quality, *QM*(2) = 0.81, *p* = 0.668. See Figure 2 for forest plots by depression.

**Figure 3:**
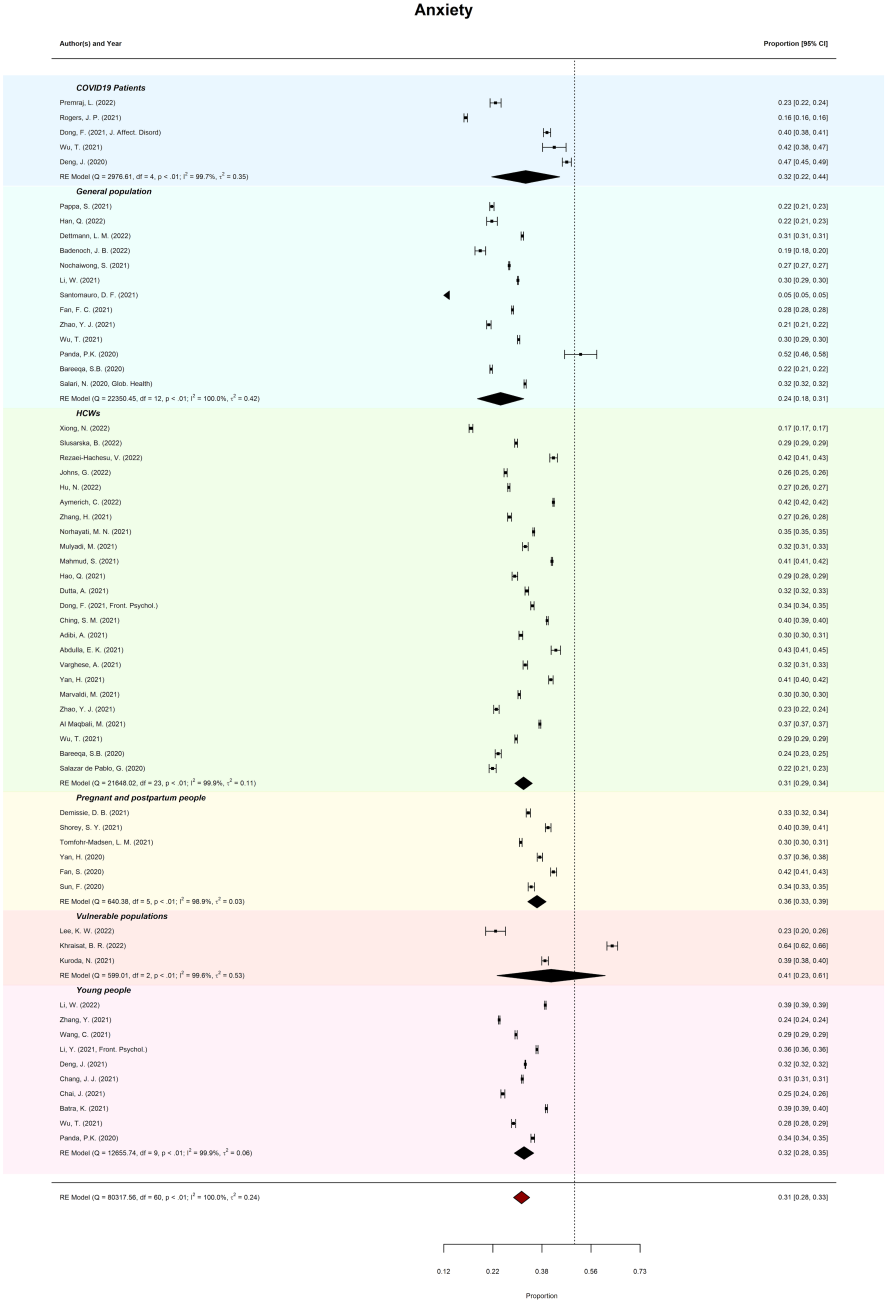
Meta-review of pooled prevalence of anxiety. The forest plots of probable anxiety prevalence (with 95% CIs) across all subgroups.

Meta-review of overall probable anxiety prevalence (*k* = 61) was 30.75% (95%CI = 28–33%; PI = 14–54%). There was considerable between-review heterogeneity (*I*^2^ *=* 99.97%; *Tau*^2^ = 0.24; QE(60) = 80317.56, *p* < 0.001). Subgroup analysis revealed prevalence of anxiety ranged from 24.35% (general populations) to 41.09% (vulnerable populations). Meta-regression moderation analysis indicated no significant difference in prevalence estimates by review quality, *QM*(2) = 0.62, *p* = 0.735. See Figure 3 for forest plots by anxiety.

Meta-review of overall stress prevalence (*k* = 18) was 39.10% (95%CI = 34–44%; PI = 21–60%). There was considerable between-review heterogeneity (*I*^2^ = 99.91%; *Tau^2^* = 0.19; QE(17) = 4910.25, *p* < 0.001). Subgroup analysis revealed prevalence of stress ranged from 25.09% (young people) to 40.87% (HCW). Meta-regression moderation analysis indicated no significant difference in prevalence estimates by review quality, *QM*(2) = 3.59, *p* = 0.166.

**Figure 4:**
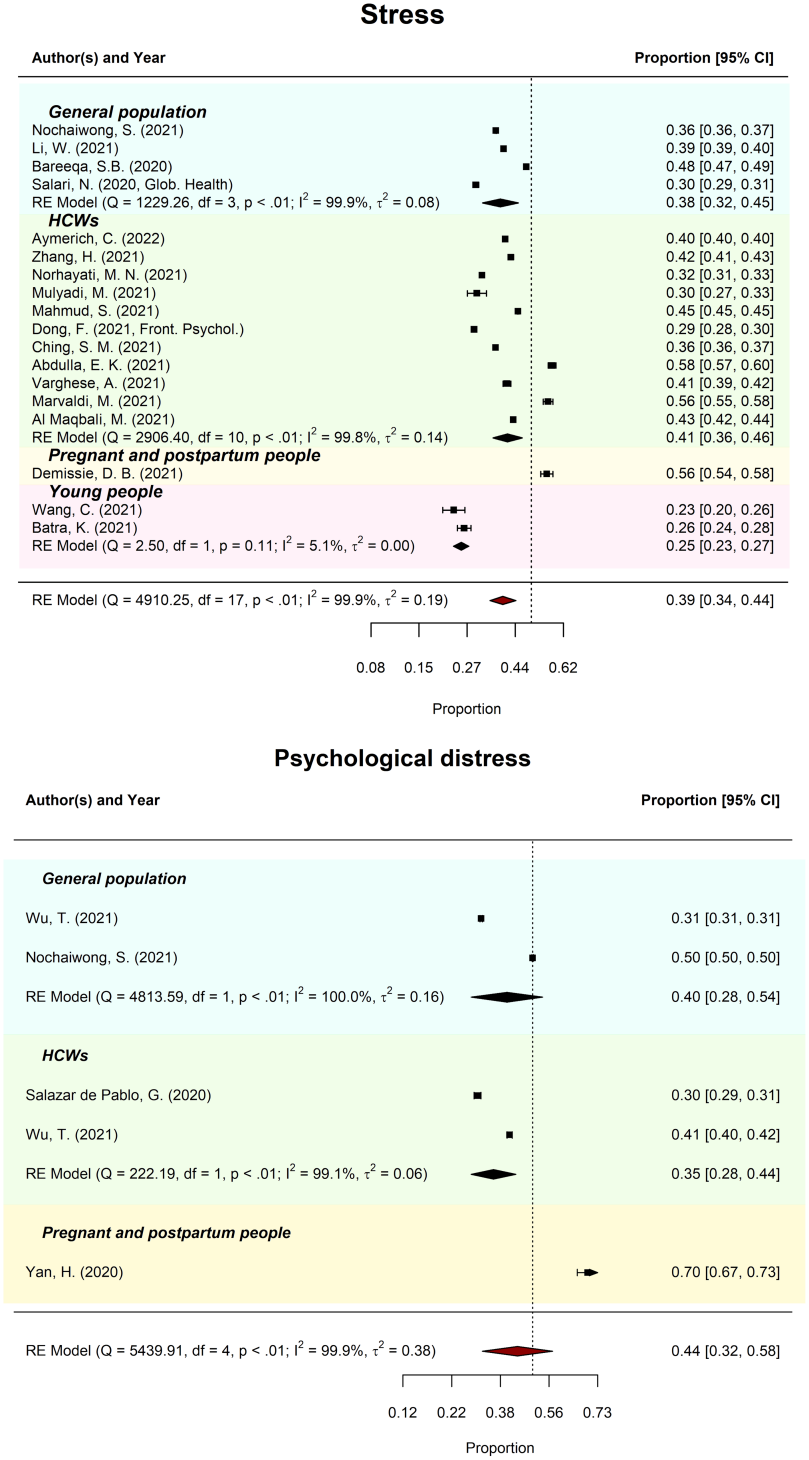
Meta-reviews of pooled prevalence of stress and psychological distress. The forest plots of the pooled prevalence (and 95% CIs) for stress across the general population, healthcare workers (HCW), pregnant and postpartum people, and young people (above) and psychological distress amongst the general population, HCW, and pregnant and postpartum people (below).

Meta-review of overall probable psychological distress prevalence (*k* = 5) was 44.17% (95%CI = 32–58%; PI = 17–75%). There was considerable between-review heterogeneity (*I*^2^ = 99.95%; *Tau*^2^ = 0.38; QE(4) = 5439.91, *p* < 0.001). Subgroup analysis revealed prevalence of psychological distress ranged from 35.36% (HCW) to 40.19% (general populations). Meta-regression moderation analysis indicated no significant difference in prevalence estimates by review quality, *QM*(2) = 0.19, *p* = 0.665. See Figure 4 for forest plots by stress and psychological distress.

**Figure 5:**
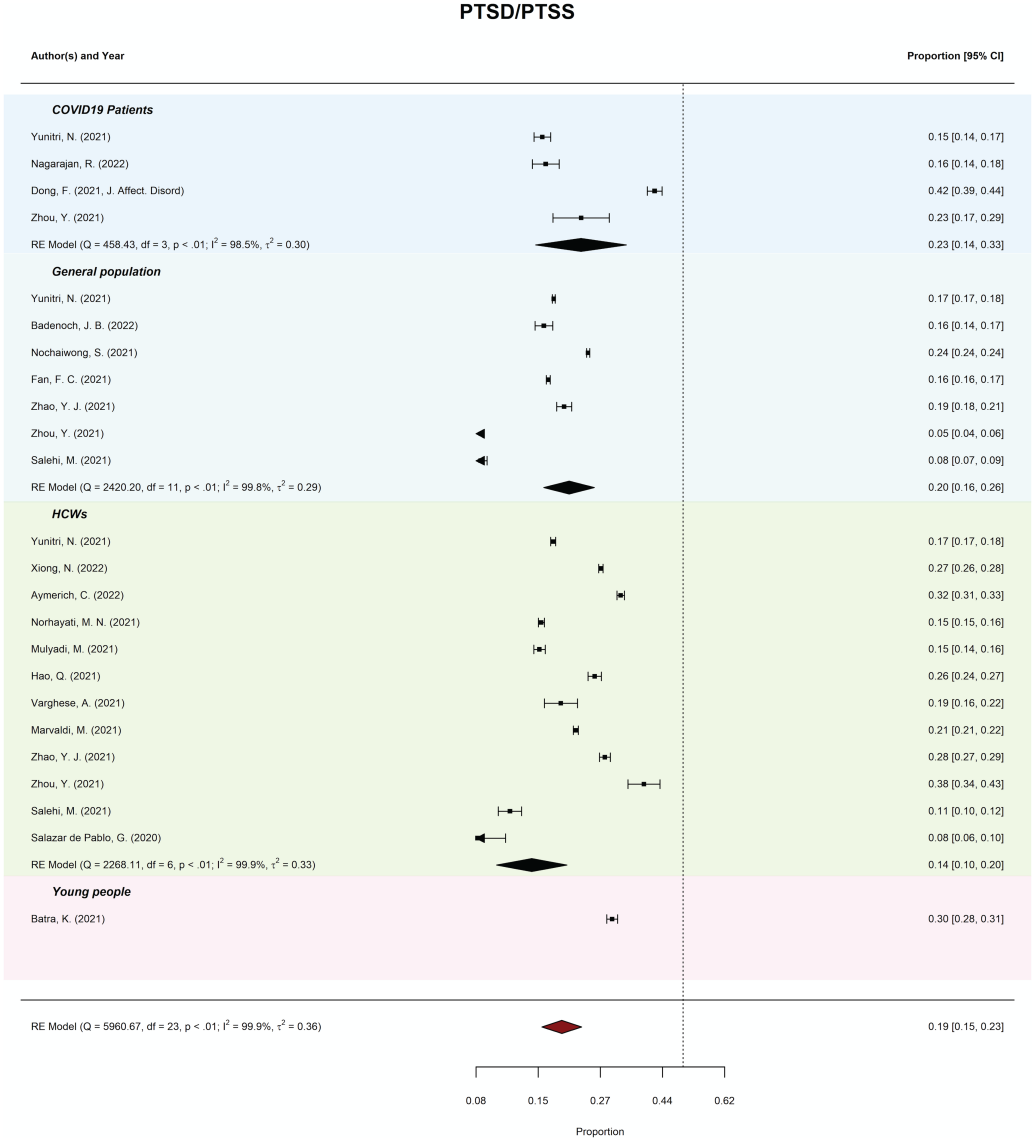
Meta-review of pooled prevalence of PTSD/PTSS. The forest plots of the prevalence (and 95% CIs) of probable post-traumatic stress disorder (PTSD) combined with post-traumatic stress symptoms (PTSS) (PTSD/PTSS) across all subgroups.

Meta-review of overall probable PTSD/PTSS prevalence (*k* = 24) was 18.77% (95%CI = 15–23%; PI = 7–43%). There was considerable between-review heterogeneity (*I*^2^ = 99.87%; *Tau^2^* = 0.36; QE(23) 748.80, *p* < 0.001). Subgroup analysis revealed prevalence of PTSD/PTSS ranged from 13.84% (general populations) to 32.10% (young people). Meta-regression moderation analysis indicated no significant difference in prevalence estimates by review quality, *QM*(2) = 0.40, *p* = 0.820. See Figure 5 for forest plots by PTSD/PTSS.

Each model was re-run with ‘mixed’ and un-categorizable population groups added (see Supplementary Table S13 and Supplementary Figures S1–S5 for full results). Briefly, supplementary model estimates were largely similar to the main models presented above, except for psychological distress, which had a lower pooled prevalence and a wider prediction interval.

### Meta-review: Comparison to pre-pandemic levels of mental ill-health

**Figure 6:**
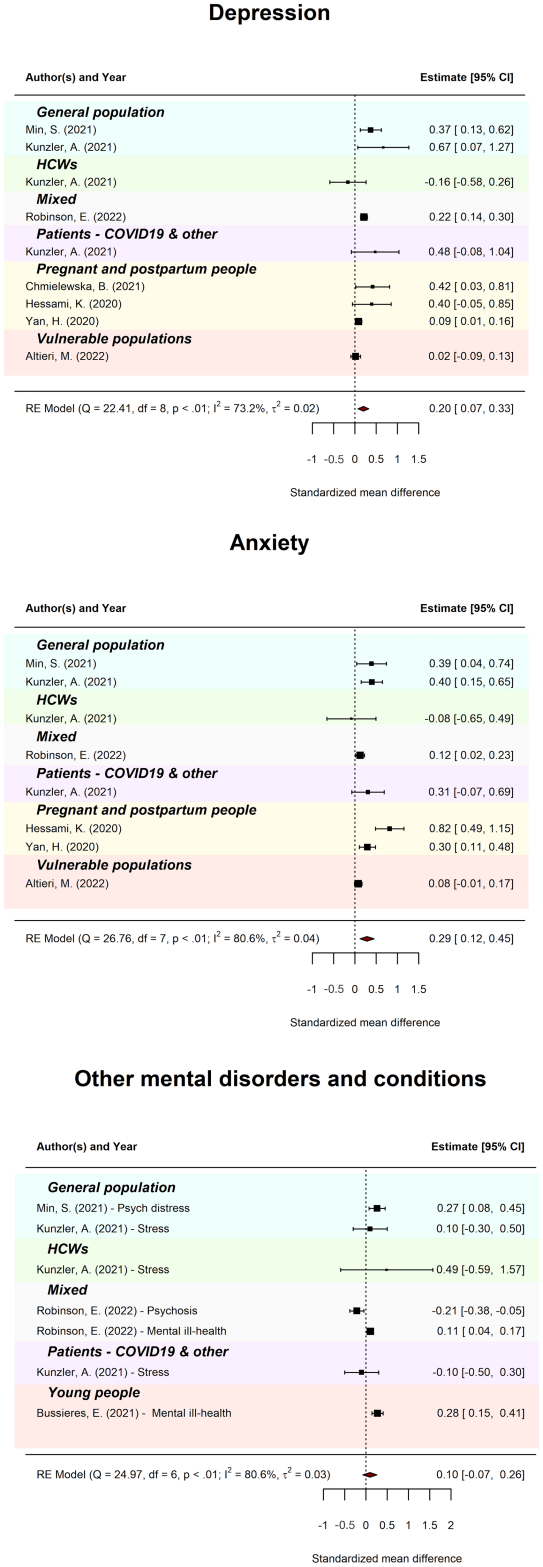
Meta-review of pooled standardised mean difference comparing COVID-19 to pre-COVID-19 levels. The forest plots of pooled standardised mean difference (SMD) comparing COVID-19 levels to pre-COVID-19 levels for probable depression, anxiety, and other mental disorders across all subgroups. The 95%CI is indicated by the rightmost, and leftmost points of the diamond.

Table 1 displays pooled standardised mean difference (SMD) and heterogeneity statistics for meta-review models of combined meta-analyses that reported an effect of COVID-19 compared to pre-pandemic. Figure 6 displays forest plots by outcome. Meta-review of the effect of COVID-19 on overall probable depression (*k* = 9) revealed an SMD of 0.20 (95%CI = 0.07–0.33; PI = −0.09–0.49). There was substantial between-review heterogeneity (*I*^2^ = 73.21%; *Tau*^2^ = 0.02; QE(8) = 22.41, *p* < 0.01). Meta-review of the effect of COVID-19 on overall probable anxiety (*k* = 8) revealed an SMD of 0.29 (95%CI = 0.12–0.45; PI = −0.13–0.70). There was considerable between-review heterogeneity (*I*^2^ = 80.59%; *Tau*^2^ = 0.04; QE(7) = 26.76, *p* < 0.001). Meta-review of the effect of COVID-19 on other overall mental health conditions (*k* = 7) revealed an SMD of 0.10 (95%CI = −0.07, 0.26; PI = −0.29–0.48). There was considerable between-review heterogeneity (*I*^2^ = 80.58%; *Tau*^2^ = 0.03; QE(6) = 24.97, *p* < 0.001).

### Narrative synthesis of mental health impacts: Population-level findings

#### Suicidal thoughts and behaviours

One review described six studies investigating the impact of COVID-19 on suicidal thoughts and self-harm behaviours in the general population, with four showing significant increases (36). Two nationally-representative samples (United States and Czech) comparing 2017 and 2020 prevalence data found that suicidal ideation increased from 3.4 to 16.3% [no CIs] in the United States (higher amongst those experiencing unaffordable housing, job loss and loneliness), and in the Czech Republic moderate-to-high suicide risk increased from (3.9% [95%CI = 3.2–4.5%] to 12.3% [95%CI = 11.1–13.4%]). The high quality and consistency of evidence provided gives us low-to-moderate confidence COVID-19 was associated with increased suicidal ideation and risk in the general population.

#### Proximity to COVID-19 infection, fear of infection and confirmed infection

Three reviews provided evidence that fear of COVID-19 infection was associated with poor mental health. A meta-analysis of global studies showed that while fear of COVID-19 was generally low, it was moderately correlated with anxiety (*d* = 0.54, *K* = 48, 95%CI = 0.48–0.61, *I*^2^ = 97.6%), depression (0.40, *K* = 49, 95%CI = 0.35–0.44, *I*^2^ = 95%) and stress (0.42, *K* = 19, 95%CI = 0.35–0.50, *I*^2^ = 92.6%) (44). Another review, citing a cross-sectional study of Turkish adolescents (*n* = 598), found an association between fear of COVID-19 and higher ‘depression-anxiety’ scores (*β* = 0.81, *p* < 0.01) (31). One review cited an Indian case-series study (*n* = 72) where fear of infection was a primary antecedent to suicide (*n* = 21) (36). We have low confidence in the strength of evidence that fear of COVID-19 infection was associated with poor mental health due to high heterogeneity in estimates and limited information about study quality.

Numerous reviews found varied prevalence estimates of probable mental disorders and symptoms amongst those currently-infected with COVID-19. One meta-analysis of Chinese studies (*n* = 5,153, *K* = 31) reported pooled prevalence of depression (45, 95%CI = 37–54%, *I*^2^ *=* 96%) and anxiety symptoms (47, 95%CI = 37–57%, *I*^2^ = 97%) in COVID-19 patients (30). Subgroup analyses found single-arm cohort studies (*K* = 3) yielded significantly higher symptoms compared to cross-sectional studies whereby COVID-19 patients showed higher symptoms than the general population, with no evidence of gender differences. Confidence in these results were limited by the variable quality of included studies, high risk of bias, and between-study heterogeneity for outcome measures. Another meta-analysis of global studies (*n* = 4,318, *K* = 22) had substantial overlap with the former review, but reported substantially lower pooled prevalence of depression, anxiety, and comorbid depression/anxiety symptoms amongst non-representative samples of COVID-19 patients as 38% (95%CI = 25–51%, *I*^2^ = 98%), 38% (95%CI = 24–52%, *I*^2^ = 98%), and 29% (95%CI = 0–69%; *I*^2^ = 99%), respectively (45). While high-quality studies produced higher prevalence of anxiety and depressive symptoms than low-quality studies, considerable between-study heterogeneity and minor publication bias reduces confidence in the overall findings. The differences in estimates between both meta-analyses may be attributed to the more stringent inclusion criteria of the second review (e.g., higher minimum sample size) and later publication date, increasing availability of primary studies. Another review cited a fair-quality cross-sectional study of Turkish hospitalised COVID-19 patients (*n* = 281), finding 42 and 35% had depression and anxiety symptoms, respectively (heterogeneity data not reported) (46).

A review by John, Okolie (36) (*K* = 4) investigated currently-infected people using convenience sampling, suggesting higher levels of self-harm and suicidal ideation and behaviours in COVID-19 patients than in the general population. Three included studies used small Chinese samples (*n* = 106–376) of COVID-19 patients, compared with non-infected controls or existing general population data. The fourth study, adopting a large, closely-representative United Kingdom-based sample (*n* = 44,775), found those with COVID-19 infection were more likely than those without to engage in suicidal/self-harm thoughts (33% vs. 17%) and suicide attempts (14% vs. 5%) (no heterogeneity indicators provided). Confidence in this evidence linking COVID-19 infection to poor mental health is limited by inappropriate comparisons made between often non-equivalent populations and failing to account for potential confounders for poor mental health, including stigma associated with COVID-19 infection.

COVID-19 hospitalisation may worsen the mental health impacts of infection. Drawing on a good-quality, large US-based retrospective cohort study examining hospital data(*n* = 200,000+), Veazie at al (46) concluded that hospitalised patients are at increased risk of new psychiatric diagnosis, relative to outpatients. This study examined the incidence of first-time psychiatric diagnoses at 6 months post-hospitalisation: 4.5% were diagnosed with a mood disorder, 6.9% with an anxiety disorder, 2.1% with a substance use disorder and 0.9% with a psychotic disorder. The cohort design and large sample size yields moderate confidence in the strength of evidence.

Several reviews found that most individuals do not develop new-onset psychiatric diagnoses in the months post-COVID-19 infection. Cross-sectional data included within reviews revealed substantial heterogeneity in reported prevalence of depression symptoms within 3 months post-discharge from hospitalisation with COVID-19 infection. Depression symptoms ranged from 9 to 65% in one review (46) but 11–28% (or 3–12% for clinically-significant depression) in another (42). Conversely, one review reported fairly-consistent symptom prevalence across other disorders in original studies: 30–39% anxiety, 9.5–15.4% PTSD,19.6–26% obsessive compulsive disorder (46).

Relying primarily on cross-sectional associations from two reviews, identified risk factors for poorer mental health post-infection with COVID-19 included: female gender (42, 46), psychiatric history, and degree of systemic inflammation during the acute infection phase (42). There was mixed evidence around patient age, infection acuteness, hospitalisation length, or neurocognitive impairment during infection as moderators of mental health outcomes (42, 46). Due to high heterogeneity, poor-quality of original studies, reliance on cross-sectional samples, and lack of unexposed control groups in the aforementioned reviews, we ascribe very-low confidence in the strength of evidence around these risk factors. (42, 46).

#### Access to news, information, and media

In one review, Chinese adolescents with lower depression and anxiety symptoms tended to have greater knowledge about COVID-19 control measures (31). Another review found that news and social media exposure were predictive of adverse mental health outcomes amongst HCW, particularly when these were not sites of support or helpfulness (33). However, lack of sample representativeness and reliance on cross-sectional data yields very low confidence in the strength of this evidence.

#### Alcohol and drug use disorders

Two reviews found evidence of increased incidence of new-onset substance use disorders (SUD) during COVID-19.One review, citing longitudinal mental health patient records of older people in the United Kingdom (*n* = 336), found while average alcohol use did not differ significantly from pre-pandemic (2019–2020), the prevalence of hazardous drinking decreased (17–8%), while probable alcohol dependency increased (19–28%) (47). Another review, citing a good-quality United States-cohort study of COVID-19 survivors (*n* = 200,000+) 6 months post-hospitalisation, found 2.1% had been diagnosed with a new-onset substance use disorder (46). The longitudinal nature of these data provides moderate confidence in the association between COVID-19 and development of probable substance use disorder/s.

#### Containment measures

One review cited a large United States-based cross-sectional ‘natural experiment’ (*n* = 10,625 using quota convenience sampling) comparing suicidal thoughts/attempts amongst residents of States with stay-at-home orders or gathering restrictions compared with States without these measures, finding no evidence of increased risk (36). Conversely, another review found consistent, but cross-sectional associations between lockdowns and immediate psychiatric symptoms including acute anxiety and depression symptoms in six of seven studies (the seventh included a very small sample, *n* = 26) (40).

Five of these studies (3 cross-sectional, 2 longitudinal) reported risk factors for adverse mental health outcomes including stress, loneliness, female gender, worse self-perceived health, poor physical health, pre-existing psychological disorders, lower educational levels, lower household income and economic fallout (40). The review was unclear about how risk factors were identified, providing very-low confidence in these findings. Another review suggested lockdowns could be psychologically protective for adolescents; a prospective cohort study of 14,241 adolescents identified reductions in depression symptoms (51.5–38.3%) and anxiety symptoms (38.5–23.7%) from pre-pandemic to post-confinement (all *p* < 0.0001) (31), however authors note the findings should be interpreted carefully due to high attrition (31).

Muehlschlegel and colleagues (40) cited one cross-sectional study of Chinese adults (*n* = 2,839) and one nationally representative longitudinal study of United States adults (*n* = 7,319), both finding psychiatric symptoms usually reduced or returned to baseline after infection-control/containment measures were lifted. Detail on effects and heterogeneity were not provided. Conversely one Chinese longitudinal study of students (*n* = 173) reported *reduced* stress, depression and anxiety symptoms during lockdown, which *increased* once lockdown ended (40).

Only one review examined social distancing measures, finding that among 683 United States adolescents, degree of social distancing had no association with anxiety (*R*^2^ = 0.083; 95%CI = −0.2–0.15; *p* = 0.09) or depression (*R*^2^ = 0.057; 95%CI = −0.28–0.05; *p* = 0.09) (31).

One review (*K* = 114, *N* = 640,037, 33 countries) found timing of government intervention moderated mental health outcomes (38). The prevalence of clinically-significant depressive symptoms was significantly lower in countries with rapid COVID-19 containment policies, after accounting for national COVID-19 cases at study commencement, Healthcare Access and Quality index, participants infected with COVID-19, and withstanding sensitivity analyses removing Chinese studies (*Q_E_* = 9485.92, *p* < 0.001; *R*^2^ = 33.61%). (38) While over 70% of included studies had serious risk of bias, the large number of included studies and appropriate confounders provide low-to-moderate confidence in this evidence.

One review including 36 studies (11 countries) with pre-pandemic comparison data, (*n* = 79,781 students, 18,028 parents) concluded that school closures appeared to increase anxiety or depression symptoms (48). Due to concurrent lockdowns and the observational nature of these studies, effects could not be disentangled from the impacts of broader lockdown measures (48). Two high-quality cohort studies found no significant increase in national suicide rates during school closure and lockdown compared with historical control periods in England and Japan (48). However, a high-quality cross-sectional United Kingdom study found rates of depression, anxiety, and trauma symptoms above population thresholds amongst 13–18-year-olds during school closures. Risk factors for poor mental health identified through cross-sectional studies included female gender, pre-pandemic mental health and connection to school (48).

#### Geographic region

Several reviews found that mental health outcomes differed as a function of a participant’s country’s Human Development Index (HDI), a composite score of inhabitants’ life expectancy, education, and income (UNDP, 2022). One review including 33 countries (*K* = 114, *N* = 640,037) found clinically-significant depressive symptoms were higher in ‘very-high’ HDIs and lower in ‘medium’ or ‘low’ HDI countries, relative to countries with a ‘high’ HDI (38), however another review (*K* = 107, *n* = 398,771, 32 countries) found mental health problems (depression, anxiety, PTSS, and psychological distress) were higher in low-to-medium HDI countries (41).

The latter review’s high-quality subgroup analysis also identified other disparities between countries, finding higher probable mental ill-health in countries with a high gender inequality index, stringent government response index, low-to-medium hospital beds per 10,000 people, low-to-medium current health expenditure, estimated percent change of real GDP growth 2020 below −3.0, low resilience (fourth-quartile) of business environment, medium resilience (second-quartile) of business environment, and high economic vulnerability-inbound tourism expenditure (41). While the design and rigour of both reviews is of moderate quality, the lack of available original studies containing prevalence data from ‘very-low’ HDI countries and pre-pandemic baseline data, precludes generalisations of these findings. As such, there is overall low confidence in the evidence that COVID-19 differentially impacted the mental health of different geographic regions by HDI (38).

### Narrative synthesis of mental health impacts: Subpopulation-level findings

#### Children, young people, and students

Reviews suggested youth mental health likely worsened during COVID-19, except for a finding pertaining to home confinement. One review included 18 studies from 19 countries/regions, finding adolescent psychiatric service use fell, including emergency department presentations for self-harm and suicidal ideation (49). However, the authors concluded that this reduction was not an indicator of improved adolescent mental health, but due to ‘stay-at-home’ orders and reduced service availability and highlight that ED presentations for suicide attempts had increased (49).This same review included a study across 10 countries/regions that found a 7% increase in the proportion of children and young people within total psychiatric ED visits from 2019 to 2020 (49). Yet, this review examined percentage scores change within included studies, and did not weight scores based on study factors, such as sample sizes, because of high study heterogeneity (49). Moreover, considerations were not made about the representativeness of data, limiting generalisations that can be made (49).

Despite findings that ED presentations for suicidal ideation decreased in the aforementioned review, one review included a longitudinal Chinese cohort study of 1,271 school children, finding significant increases in odds of non-suicidal self-injury (42.0% vs. 31.8%; aOR, 1.35 95%CI = 1.17–1.55; *p* < 0.001), suicidal ideation (29.7% vs. 22.5%; aOR, 1.32 95%CI = 1.08–1.62; *p* = 0.008), plans (14.6% vs. 8.7%; aOR, 1.71 95%CI = 1.31–2.24; *p* < 0.001), and attempts (6.4% vs. 3.0%; aOR, 1.74 95%CI = 1.14–2.67; *p* < 0.001) in 2020 compared to 2019 (36). Despite unclear evidence about the representativeness of this cohort, a strong follow-up rate (93.1%) increases the validity of these findings.

As discussed in ‘Containment Measures’, one review which included a longitudinal study of adolescents found home confinement due to COVID-19 was correlated with lower anxiety and depression symptoms relative to pre-pandemic levels (31).

One meta-analysis investigated the mental health of college students in China over the first few months of the pandemic (January to May 2020). The prevalence of probable depression was 39% (95%CI = 27–51%, *K* = 18, *I*^2^ = 99.9%, *p* ≤ 0.01) and probable anxiety was 36% (95%CI = 26–46%, *K* = 20, *I*^2^ = 99.9%, *p* ≤ 0.01) (39). The review found that the prevalence of depression was significantly higher after 1 March 2020 than before then (21, 95%CI = 16–25% and 19, 95%CI = 13–25%, *I*^2^ ≥ 99.4). Conversely, John et al (36) narratively synthesised findings from two longitudinal surveys consisting of samples from 11 universities in Poland and Canada and found no evidence of increases in suicidal thoughts or behaviours over the course of the pandemic, however suicide register data from Japan revealed a 47% increase in student suicides in 2020 in comparison to 2017–2019 data. Given the substantial variation in findings in different geographic regions, at present there is low confidence in a global mental health impact on students, as differences identified in reviews may be due to other local factors.

While the quality of evidence that the pandemic had a negative impact on the mental health of children, young people and students is buoyed by some longitudinal data, it is also limited by a lack of representative studies, leading to low confidence in this evidence.

One review described protective and risk factors for anxiety and depression among young people based on a single cross-sectional study of Chinese adolescents (*n* = 8,079) (31). Protective factors for anxiety and depression included living in cities and knowledge of COVID-19 control measures (31). Risk factors included female gender and living in communities with high COVID-19 case numbers (31). A lack of pre-pandemic baseline data and inconsistency in results yields very low confidence in the generalisability of these findings.

#### People with pre-existing mental health or substance use issues

Several reviews reported worse mental health outcomes during the pandemic period among those with pre-existing mental disorders compared to those who did not have a pre-existing disorder (36, 40, 42). However, these inferences were drawn from predominantly cross-sectional studies or longitudinal studies with no pre-pandemic baseline; the data used shows a higher incidence of mental ill-health at the timepoints measured, not a comparison of the amount of increase between the two groups. As such, this provides very-low confidence in the finding that people with pre-existing mental disorders experienced greater pandemic-related mental health impacts than people without pre-existing disorders.

Two reviews reported evidence that pre-pandemic alcohol use disorders (AUD) were a risk factor for increased alcohol consumption during the pandemic (47, 50). One of these was a narrative synthesis of European studies, which included a retrospective cohort study among specialist hospital outpatients and found chemical screening indicators for relapse almost doubled during the lockdown period and another study among people seeking SUD treatment that found 12.5% reported an increase in consumption relative to the 6 months prior to lockdown, however 66% self-reported no change. A cross-sectional study comparing those at risk of AUD with moderate drinkers showed consumption did not change significantly from pre-pandemic levels, however another study showed that participants at risk of AUD were more likely than moderate drinkers to reduce their consumption post-lockdown (47). Despite the overall findings between these two reviews aligning, the designs of included studies were predominantly cross-sectional or retrospective cohort studies, thus providing low-to-moderate confidence that having a pre-existing AUD/SUD was associated with an increased risk of greater consumption during the pandemic period.

#### Pregnancy and parenthood

A meta-analysis of global studies examining mothers of children under 5-years-old yielded a pooled prevalence of 26.9% (95%CI = 21.3–33.4%, *K* = 16, *I*^2^ = 96.52%) for clinically-elevated depressive symptoms and 41.9% (95%CI = 26.7–58.8%, *K* = 8, *I*^2^ = 99.07%) for clinically-elevated anxiety. Elevated depression and anxiety symptoms were significantly higher in Europe and North America than South America, the Middle East and Asia and amongst older mothers (51). Depressive symptoms were significantly lower in studies with a higher percentage of racial minority mothers, whereas anxiety symptoms were higher among low-quality studies and in samples with highly-educated mothers (51). No reviews assessed the impact of COVID-19 on parental mental health by comparing pandemic with pre-pandemic data.

Drawing on longitudinal data, two reviews showed negative impacts of COVID-19 on the mental health of pregnant people. One review, which compared pre-pandemic and pandemic data, cited seven of 11 studies reporting a statistically significant increase in postnatal depression, maternal anxiety, or both (34). Pooled prevalence data of three studies showed the pooled mean difference from pre-pandemic was 0.42 (95%CI = 0.02–0.81, *I*^2^ = 79%, *p* = 0.038). The review found a statistically significant increase in mean Edinburgh Postnatal Depression Scale score within low-to-middle income countries of 0.22 (95%CI = 0.21–0.23), relative to countries with higher incomes (34). Another review reported higher levels of suicidal/self-harm thoughts reported in third-trimester pregnant women (*n* = 4,124) compared to third-trimester women pre-pandemic (*n* = 2,839; aRR = 2.85, 95%CI = 1.70–8.85, *p* = 0.005) (36). The use of cohort data and high consistency of findings yields moderate confidence in the negative mental health impacts of the pandemic on perinatal people.

#### Healthcare workers

Reviews examining the mental health of HCW during COVID-19 described the current state of evidence as poor, with few studies utilising pre-pandemic data or representative, non-convenience sampling (32, 33, 35, 37, 52–54). Consequently, there is very-low confidence in reported results pertaining to this population. One review meta-analysed cross-sectional mental health data on Chinese HCW across the first 4 weeks of the pandemic, finding psychological distress prevalence peaked early and subsequently slowly reduced.

Two systematic reviews citing cross-sectional studies provided conflicting evidence for HCW mental health outcomes during COVID-19 relative to other groups. One review found HCW were just as, or significantly less, likely to report mental ill-health than those in other occupations (37). Another found a higher prevalence of psychological symptoms in HCW than the general population during lockdown (40). A meta-regression found HCW appeared more susceptible to depression and stress, but not anxiety, associated with fear of COVID-19, than the general population (44).

Six reviews identified occupational, demographic, psychological and infection-based risk and protective factors for mental health amongst HCW (available in S1 Results). While based primarily on cross-sectional studies, several findings were consistently associated with mental ill-health, increasing our confidence in their reliability as evidence. These included exposure to COVID-19 patients (32), particularly in a high epidemic locations (33) – which may be a more important distinction than frontline versus non-frontline roles for which evidence was mixed (35) – and poor organisational support (33, 37, 52, 53). Perceived personal and occupational support and positive co-worker attitude was protective (33, 35, 52).

**Figure 7:**
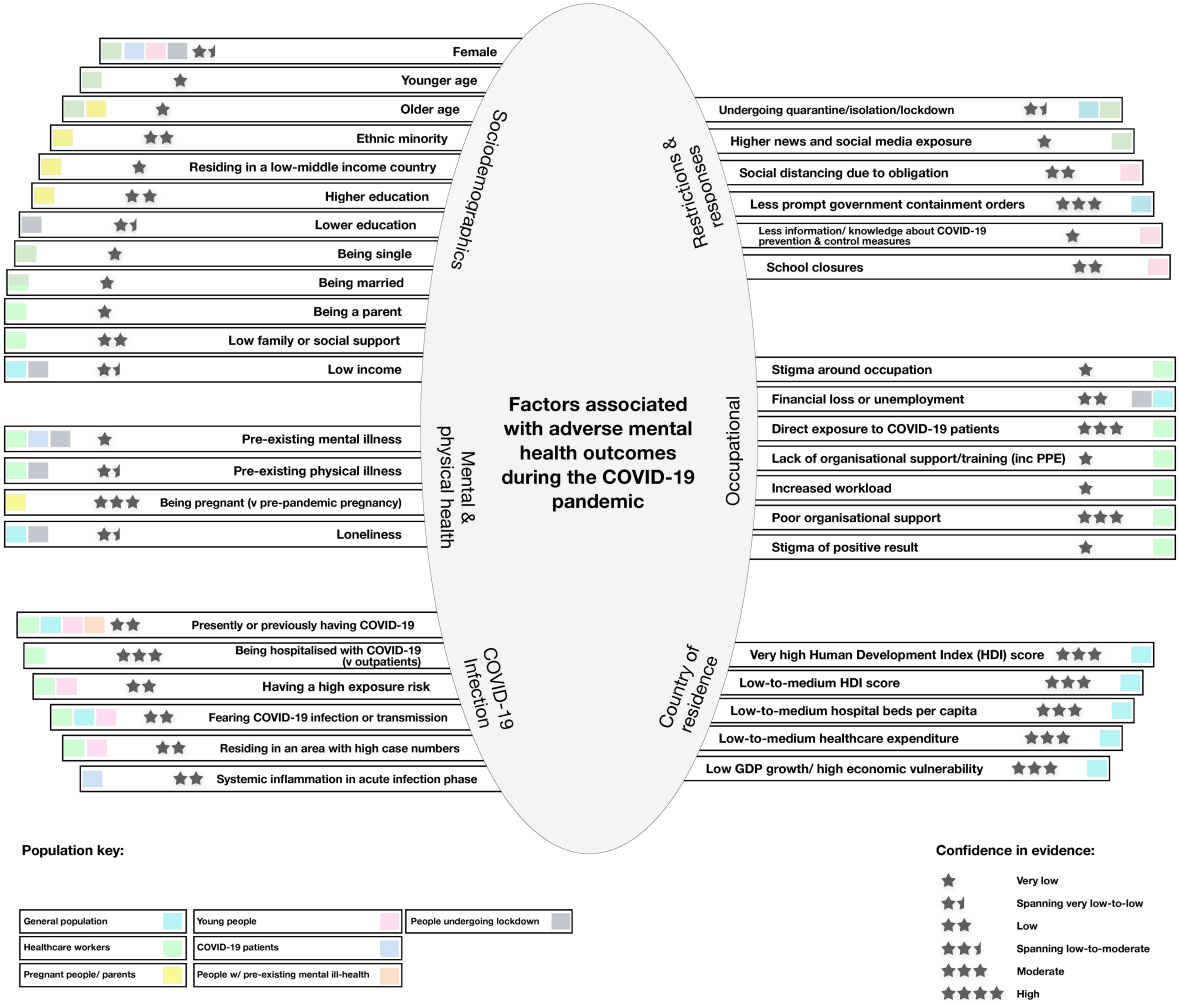
Diagrammatic representation of factors associated with adverse mental health outcomes during the COVID-19 period, as identified through narrative synthesis. A diagrammatic representation of the factors associated with adverse mental health in the COVID-19 period, as identified through narrative synthesis. The key has color coding for population group in which the association was investigated. The stars show the level of confidence in the findings, based off the Synthesis Without Meta-analysis (SWiM) method, in addition to the AMSTAR-2 quality appraisal.

A diagrammatic representation of the factors associated with adverse mental health in the COVID-19 period, as identified through narrative synthesis, is shown in Figure 7.

## Discussion

This umbrella review aimed to synthesise available evidence and draw conclusions on the overall impact of COVID-19 on mental health. This is the first meta-review to report a pooled comparison of mid-pandemic to pre-pandemic prevalence of mental ill-health. We identified 338 reviews, including 180 systematic reviews, 32 meta-analyses and 126 systematic reviews with meta-analyses. Following extraction and appraisal, 25 reviews of moderate quality were narratively synthesised and 83 meta-analyses were meta-reviewed. Review quality did not significantly moderate findings. Overall, the meta-review of pooled prevalence indicated that individuals globally experienced probable anxiety (30.8%), depression (28.1%), stress (39.1%) psychological distress (44.2%), and PTSD/PTSS (18.8%) during the pandemic. Meta-review findings showed that probable mental disorders appeared to increase significantly during COVID-19 compared to pre-pandemic. Narrative review evidence suggested an increased incidence of new-onset substance use disorders and increased suicidal thoughts and assessed risk. This latter finding is at odds with previous longitudinal global data during the first 9–15 months of the pandemic, showing no evidence of greater-than-expected numbers of suicide deaths (55). This discrepancy may indicate that increased suicidal thoughts did not translate into suicide deaths.

While existing umbrella reviews have mostly examined HCW mental health, this review found little evidence that this group had a higher prevalence of probable mental disorders than other groups, indicating similar or lower prevalence than the general population. Across multiple outcomes, vulnerable populations and young people demonstrated higher pooled prevalence of probable disorders relative to other populations. Using moderate quality evidence, the narrative review identified risk factors for mental ill-health, including hospitalisation with COVID-19, pregnancy, adolescence, and geographic region (e.g., countries with more delayed COVID-19 containment policies). Lockdowns appeared to be associated with poorer mental health, but there was little evidence this persisted once lockdowns eased. Female gender and pre-existing mental health issues were consistently associated with poorer mental health during the pandemic. However, people with mental disorders are by definition symptomatic and female gender is associated with poorer mental health irrespective of the pandemic (56); the lack of high-quality longitudinal data and pre-pandemic baseline data in studies identifying these risk factors precludes inference that the pandemic widened disparities amongst these populations. This umbrella review had several strengths. It fills an important gap in the literature as the first meta-review to pool comparisons between mid-COVID-19 to pre-COVID-19 prevalence of mental health conditions. It reports the most extensive examination of population groups and systematically summarised current evidence for the mental health impacts of COVID-19 using a robust search strategy and rigorous methodology. All studies were independently double-reviewed and assessed using a validated quality appraisal tool (19, 20). CCA analysis was used to calculate degree of primary study overlap across included reviews (27). The SWiM approach guided the narrative synthesis, increasing transparency around methodologies used to synthesize systematic review findings (21). Moderator analyses were conducted to investigate whether review quality moderated the meta-review effect sizes reported.

Findings of the current review should be interpreted with caution given their broad limitations. The validity and accuracy of the meta-review pooled prevalence and pre-pandemic comparison estimates are limited by the low quality of, and high heterogeneity (with most *I*^2^ estimates ≥99%) between, included reviews, and the considerable within-review heterogeneity between included studies, reducing confidence in true estimates (Supplementary Tables S12, S14). Heterogeneity may be due to wide variance in measurement times, screening tool/instrument variation and the populations assessed. Additionally, we cannot confirm that the meta-review pooled effects are correct, as authors may publish meta-analytic mistakes (57). Despite a rigorous synthesis approach using gold standard frameworks and protocols, as well as double-applied AMSTAR-2 quality appraisals for each review, we cannot know whether these were performed without error.

Further, most reviews utilised non-representative samples, which likely inflated or biased prevalence estimates, with possible oversampling of those with mental disorders and/or under-sampling of populations most in need (58). However, rates are consistent with a recent paper showing increased prevalence of anxiety and depression across 204 countries, highlighting consistent, adverse mental health effects of COVID-19 (2).

Included reviews’ primary studies almost exclusively provided cross-sectional prevalence data published early in the pandemic, without comparison to pre-COVID baseline data or exploring changes over time. Longitudinal research would allow for the exploration of persisting mental health-related phenomena, such as the cumulative impacts of different viral strains, multiple lockdowns, habituation, and long-term socio-emotional impacts of working from home and school closures (59–61). For example, longitudinal assessments of SARS survivors post-infection revealed long-term psychological impacts, with clear implications for clinicians and policymakers (62).

Additionally, few reviews controlled for, or disaggregated, the differential impact of COVID-19 restrictions on mental health, including the role of physical distancing, quarantine, or lockdown. Finally, only peer-reviewed reviews published in English language were included, despite higher case numbers occurring in non-English speaking countries during initial COVID-19 outbreaks. Despite the aim to provide an international perspective on mental health, there were substantial geographical gaps, particularly South America, Oceania, and Africa, which limits the generalisability of findings. Further, the range of instruments of variable psychometric quality used in primary studies is an important limitation. Finally, it should be noted that there was a departure (detailed in Methods) from the pre-registered inclusion criteria listed *a priori* in PROSPERO and details were limited surrounding planned analyses registered in the protocol.

The current review was the first to provide a detailed summary of factors and groups associated with adverse mental health outcomes during COVID-19. By identifying those most at risk of mental ill-health, including vulnerable populations, younger and pregnant people, amongst others, this review assists clinicians and policymakers in targeting resources and support now or in future pandemics. By identifying the differential mental health impacts of pandemic control measures, including aspects of quarantine and access and exposure to COVID-19 information, policymakers can modify responses accordingly to mitigate the negative impact of such measures.

This review highlighted areas that warrant further investigation. Echoing the messages of Zeng et al., there is a need for representative samples, prospective cohort studies and consistent, standardised and clinically-relevant outcome measures (63). While our search strategy covered a wide range of disorders and populations, many of the outcomes (e.g., eating disorders and childhood behavioural disorders) and other vulnerable groups (e.g., children, essential workers, people experiencing housing issues) were absent or insufficiently explored within the literature and should be addressed by future research. Systematic reviews and meta-analyses hereafter would benefit from focusing on under-studied subpopulations who were likely affected by restrictions and lockdowns. Moreover, the roll-out of COVID-19 vaccines, occurring after some included reviews were published, likely reduced infection-related anxiety (64).

Notwithstanding the methodological limitations of reviewed studies outlined above, our findings suggest policymakers and clinicians should not discount the potentially ongoing impacts of COVID-19 on individual and population mental health, particularly for people who were adolescent, pregnant, post-partum during the pandemic or were hospitalised with COVID-19. The resultant need for continued attention and investment in the mental health of these groups echoes statements from global peak bodies and initiatives, including the World Psychiatric Association’s call for continued funding for mental health services despite COVID-19-related strains on global government budgets (65); and the United Nations Educational, Scientific and Cultural Organisation (UNESCO) *Youth as Researchers* initiative, which released ten youth-led global policy recommendations to address COVID-19 impacts on youth, including a focus on improving access to online counselling services (66).

Future research should explore the impact of vaccination on mental health, recognising global inequalities in vaccine access. Further research is also needed to determine the mediating and moderating factors influencing mental health during the pandemic to provide a more nuanced understanding of the causal mechanisms involved (67). Future research should also continue prioritising the aggregation and analysis of high-quality longitudinal data to understand the complex, continuing impacts of COVID-19 on global mental health (2).

## Author contributions

MB, SS, AD-B, and MT contributed to developing the title and research/review questions, introduction, inclusion criteria and search strategy. Study selection, assessment of methodological quality, and data extraction were conducted by MB, SS, AD-B, LT, JB, DL, and AN. Data summary was completed by MB, SS, AD-B, and SO’D. Drafting and refinement of the article was conducted by MB, SS, AD-B, SO’D, PB, AC, KG, and MT. All authors contributed to the article and approved the submitted version.

## Funding

MT, PB, and AC are supported by Australian National Health and Medical Research Council (NHMRC) Fellowships (1195284, 1158707 and 1173146). This work was supported by the BHP Foundation.

## Conflict of interest

MT, PB, and AC are members of Australia’s Mental Health Think Tank.

The remaining authors declare that the research was conducted in the absence of any commercial or financial relationships that could be construed as a potential conflict of interest.

## Publisher’s note

All claims expressed in this article are solely those of the authors and do not necessarily represent those of their affiliated organizations, or those of the publisher, the editors and the reviewers. Any product that may be evaluated in this article, or claim that may be made by its manufacturer, is not guaranteed or endorsed by the publisher.
