# Supplementary material for: A hidden pandemic? An umbrella review of global evidence on mental health in the time of COVID-19
Source: Front Psychiatry. 2023 Mar 8;14:1107560. doi: 10.3389/fpsyt.2023.1107560 (PMC10032377; doi:10.3389/fpsyt.2023.1107560)
Supplement: Supplementary file 1 [file Data_Sheet_1.docx]

**Supporting Information**

[S1 Table. Search Terms: Cochrane Database of Systematic Reviews 2](#_Toc118473117)

[S2 Table. Search Terms: PsycINFO 3](#_Toc118473118)

[S3 Table. Search Terms: Embase and Medline 4](#_Toc118473119)

[S4 Table. Search Terms: SCOPUS 6](#_Toc118473120)

[S5 Table. Results of corrected covered area analysis 7](#_Toc118473121)

[S6 Table. SWiM Table 8](#_Toc118473122)

[S7 Table. Reviews excluded from narrative synthesis due to low or critically low quality 10](#_Toc118473123)

[S8 Table. Characteristics of reviews that received a moderate AMSTAR grade 17](#_Toc118473124)

[S9 Table. Meta-analyses included in meta-review 28](#_Toc118473125)

[S10 Table. Countries distribution of original studies in meta-review 30](#_Toc118473126)

[S11 Table. Meta-analyses excluded from meta-review 46](#_Toc118473127)

[S12 Table. Heterogeneity and risk of bias information for pooled estimates included in meta-review of pooled prevalence 49](#_Toc118473128)

[S13 Table. Model results including mixed and quarantined populations 63](#_Toc118473129)

[S14 Table. Heterogeneity and risk of bias information for pooled estimates included in meta-review of standardised mean difference 64](#_Toc118473130)

[S1 Figure. Forest plot of supplementary model of probable depression pooled prevalence 66](#_Toc118473131)

[S2 Figure. Forest plot of supplementary model of probable anxiety pooled prevalence 68](#_Toc118473132)

[S3 Figure. Forest plot of supplementary model of stress pooled prevalence (no change to main model due to no additional reviews. 69](#_Toc118473133)

[S4 Figure. Forest plot of supplementary model of PTSD pooled prevalence 70](#_Toc118473134)

[S5 Figure. Forest plot of supplementary model of psychological distress pooled prevalence 71](#_Toc118473135)

[S1 Results. Further narrative synthesis detail on Healthcare workers 72](#_Toc118473136)

[S1 References. Complete list of references 73](#_Toc118473137)

## S1 Table. Search Terms: Cochrane Database of Systematic Reviews

| # | Search Statement |
| --- | --- |
| 1 | ("coronavirus disease 2019" or "covid-19" or "severe acute respiratory syndrome coronavirus 2" or "SARS-CoV-2" or "2019-nCoV").ti,ab. |
| 2 | (mental health or well-being or wellbeing or psychiatr* or psycholog*).mp. |
| 3 | (neuropsycholog* or neurolog* or anxious* or anxiety or depress* or post-traumatic or isolat* or fear* or insomnia).mp. |
| 4 | (hopeless* or panic * or hysteri* or stress* or insecurity or confine* or confus* or trauma* or irritab* or frustrat* or bored* or anger* or angry).mp. |
| 5 | (disorder* adj4 (mental or psychological or psychiatric or mood or affective or anxiety or panic or obsessive-compulsive or body dysmorphic or hoarding or hair-pulling)).tw. |
| 6 | (disorder* adj4 (excoriation or skin-picking or post-traumatic or posttraumatic or acute stress or adjustment or delusional or dissociative)).tw. |
| 7 | (disorder* adj4 (conversion or depersonali?ation or dereali?ation or eating or feeding or oppositional defiant or conduct or disruptive or impulse control)).tw. |
| 8 | (disorder* adj4 (personality or attention deficit or motor or neurocognitive or neurodevelopmental or neurotic or paraphilic or psychotic or substance-related or trauma or stressor)).tw. |
| 9 | (illness* adj4 (mental or psychological or psychiatric)).tw. |
| 10 | (mental health or depress* or dysthymi* or bipolar or cyclothymi* or mania or manic or hypomania or gad or agoraphobia or phobia* or ocd or bdd).tw. |
| 11 | (trichotillomania or ptsd or psychotic or psychos* or schizo* or somati* or somatoform or anorexi* or bulimi* or pyromania or kleptomania or adhd).tw. |
| 12 | 2 or 3 or 4 or 5 or 6 or 7 or 8 or 9 or 10 or 11 |
| 13 | ((abuse or use* or usage or disorder or depend*) adj4 (substance or drug or alcohol or marijuana or benzodiazepine or cannabis or opioid or stimulant or methamphetamine)).mp. |
| 14 | 12 or 13 |
| 15 | 1 and 14 |
| 16 | (review* adj4 (systemati* or literature or critical or mapping or scoping or umbrella or rapid or evidence-based)).mp. |
| 17 | (overview or meta-analys*).mp. |
| 18 | 16 or 17 |
| 19 | systemati*.mp. |
| 20 | 18 and 19 |
| 21 | 15 and 20 |

## S2 Table. Search Terms: PsycINFO

| # | Search Statement |
| --- | --- |
| 1 | coronavirus/ |
| 2 | ("coronavirus disease 2019" or "covid-19" or "severe acute respiratory syndrome coronavirus 2" or "SARS-CoV-2" or "2019-nCoV").mp. |
| 3 | 1 or 2 |
| 4 | exp mental health/ |
| 5 | public mental health/ |
| 6 | well being/ |
| 7 | (mental health or well-being or wellbeing or psychiatr* or psycholog*).mp. |
| 8 | (neuropsycholog* or neurolog* or anxious* or anxiety or depress* or post-traumatic or isolat* or fear* or insomnia).mp. |
| 9 | (hopeless* or panic * or hysteri* or stress* or insecurity or confine* or confus* or trauma* or irritab* or frustrat* or bored* or anger* or angry).mp. |
| 10 | (disorder* adj4 (mental or psychological or psychiatric or mood or affective or anxiety or panic or obsessive-compulsive or body dysmorphic or hoarding or hair-pulling)).tw. |
| 11 | (disorder* adj4 (excoriation or skin-picking or post-traumatic or posttraumatic or acute stress or adjustment or delusional or dissociative)).tw. |
| 12 | (disorder* adj4 (conversion or depersonali?ation or dereali?ation or eating or feeding or oppositional defiant or conduct or disruptive or impulse control)).tw. |
| 13 | (disorder* adj4 (personality or attention deficit or motor or neurocognitive or neurodevelopmental or neurotic or paraphilic or psychotic or substance-related or trauma or stressor)).tw. |
| 14 | (illness* adj4 (mental or psychological or psychiatric)).tw. |
| 15 | (mental health or depress* or dysthymi* or bipolar or cyclothymi* or mania or manic or hypomania or gad or agoraphobia or phobia* or ocd or bdd).tw. |
| 16 | (trichotillomania or ptsd or psychotic or psychos* or schizo* or somati* or somatoform or anorexi* or bulimi* or pyromania or kleptomania or adhd).tw. |
| 17 | 4 or 5 or 6 or 7 or 8 or 9 or 10 or 11 or 12 or 13 or 14 or 15 or 16 |
| 18 | "substance related and addictive disorders"/ or exp "substance use disorder"/ |
| 19 | ((abuse or use* or usage or disorder or depend*) adj4 (substance or drug or alcohol or marijuana or benzodiazepine or cannabis or opioid or stimulant or methamphetamine)).mp. |
| 20 | 18 or 19 |
| 21 | 17 or 20 |
| 22 | 3 and 21 |
| 23 | "systematic review"/ |
| 24 | meta analysis/ |
| 25 | (review* adj4 (systemati* or literature or critical or mapping or scoping or umbrella or rapid or evidence-based)).mp. |
| 26 | (overview or meta-analys*).mp. |
| 27 | 23 or 24 or 25 or 26 |
| 28 | systemati*.mp. |
| 29 | 27 and 28 |
| 30 | 22 and 29 |

## S3 Table. Search Terms: Embase

| # | Search Statement |
| --- | --- |
| 1 | "coronavirus disease 2019"/ |
| 2 | ("coronavirus disease 2019" or "covid-19" or "severe acute respiratory syndrome coronavirus 2" or "SARS-CoV-2" or "2019-nCoV").mp. |
| 3 | 1 or 2 |
| 4 | exp mental health/ |
| 5 | (mental health or well-being or wellbeing or psychiatr* or psycholog*).mp. |
| 6 | (neuropsycholog* or neurolog* or anxious* or anxiety or depress* or post-traumatic or isolat* or fear* or insomnia).mp. |
| 7 | (hopeless* or panic * or hysteri* or stress* or insecurity or confine* or confus* or trauma* or irritab* or frustrat* or bored* or anger* or angry).mp. |
| 8 | (disorder* adj4 (mental or psychological or psychiatric or mood or affective or anxiety or panic or obsessive-compulsive or body dysmorphic or hoarding or hair-pulling)).tw. |
| 9 | (disorder* adj4 (excoriation or skin-picking or post-traumatic or posttraumatic or acute stress or adjustment or delusional or dissociative)).tw. |
| 10 | (disorder* adj4 (conversion or depersonali?ation or dereali?ation or eating or feeding or oppositional defiant or conduct or disruptive or impulse control)).tw. |
| 11 | (disorder* adj4 (personality or attention deficit or motor or neurocognitive or neurodevelopmental or neurotic or paraphilic or psychotic or substance-related or trauma or stressor)).tw. |
| 12 | (illness* adj4 (mental or psychological or psychiatric)).tw. |
| 13 | (mental health or depress* or dysthymi* or bipolar or cyclothymi* or mania or manic or hypomania or gad or agoraphobia or phobia* or ocd or bdd).tw. |
| 14 | (trichotillomania or ptsd or psychotic or psychos* or schizo* or somati* or somatoform or anorexi* or bulimi* or pyromania or kleptomania or adhd).tw. |
| 15 | 4 or 5 or 6 or 7 or 8 or 9 or 10 or 11 or 12 or 13 or 14 |
| 16 | substance abuse/ |
| 17 | ((abuse or use* or usage or disorder or depend*) adj4 (substance or drug or alcohol or marijuana or benzodiazepine or cannabis or opioid or stimulant or methamphetamine)).mp. |
| 18 | 16 or 17 |
| 19 | 15 or 18 |
| 20 | 3 and 19 |
| 21 | "systematic review"/ |
| 22 | meta-analysis/ |
| 23 | (review* adj4 (systemati* or literature or critical or mapping or scoping or umbrella or rapid or evidence-based)).mp. |
| 24 | (overview or meta-analys*).mp. |
| 25 | 21 or 22 or 23 or 24 |
| 26 | systemati*.mp. |
| 27 | 25 and 26 |
| 28 | 20 and 27 |

**Search Terms: MEDLINE**

| # | Search Statement |
| --- | --- |
| 1 | COVID-19/ |
| 2 | ("coronavirus disease 2019" or "covid-19" or "severe acute respiratory syndrome coronavirus 2" or "SARS-CoV-2" or "2019-nCoV").mp. |
| 3 | 1 or 2 |
| 4 | exp mental health/ |
| 5 | (mental health or well-being or wellbeing or psychiatr* or psycholog*).mp. |
| 6 | (neuropsycholog* or neurolog* or anxious* or anxiety or depress* or post-traumatic or isolat* or fear* or insomnia).mp. |
| 7 | (hopeless* or panic * or hysteri* or stress* or insecurity or confine* or confus* or trauma* or irritab* or frustrat* or bored* or anger* or angry).mp. |
| 8 | (disorder* adj4 (mental or psychological or psychiatric or mood or affective or anxiety or panic or obsessive-compulsive or body dysmorphic or hoarding or hair-pulling)).tw. |
| 9 | (disorder* adj4 (excoriation or skin-picking or post-traumatic or posttraumatic or acute stress or adjustment or delusional or dissociative)).tw. |
| 10 | (disorder* adj4 (conversion or depersonali?ation or dereali?ation or eating or feeding or oppositional defiant or conduct or disruptive or impulse control)).tw. |
| 11 | (disorder* adj4 (personality or attention deficit or motor or neurocognitive or neurodevelopmental or neurotic or paraphilic or psychotic or substance-related or trauma or stressor)).tw. |
| 12 | (illness* adj4 (mental or psychological or psychiatric)).tw. |
| 13 | (mental health or depress* or dysthymi* or bipolar or cyclothymi* or mania or manic or hypomania or gad or agoraphobia or phobia* or ocd or bdd).tw. |
| 14 | (trichotillomania or ptsd or psychotic or psychos* or schizo* or somati* or somatoform or anorexi* or bulimi* or pyromania or kleptomania or adhd).tw. |
| 15 | 4 or 5 or 6 or 7 or 8 or 9 or 10 or 11 or 12 or 13 or 14 |
| 16 | exp Substance-Related Disorders/ |
| 17 | ((abuse or use* or usage or disorder or depend*) adj4 (substance or drug or alcohol or marijuana or benzodiazepine or cannabis or opioid or stimulant or methamphetamine)).mp. |
| 18 | 16 or 17 |
| 19 | 15 or 18 |
| 20 | 3 and 19 |
| 21 | "systematic review"/ |
| 22 | meta-analysis/ |
| 23 | (review* adj4 (systemati* or literature or critical or mapping or scoping or umbrella or rapid or evidence-based)).mp. |
| 24 | (overview or meta-analys*).mp. |
| 25 | 21 or 22 or 23 or 24 |
| 26 | systemati*.mp. |
| 27 | 25 and 26 |
| 28 | 20 and 27 |

## S4 Table. Search Terms: SCOPUS

( ( ( TITLE-ABS-KEY ( "coronavirus disease 2019"  OR  "covid-19"  OR  "severe acute respiratory syndrome coronavirus 2"  OR  "SARS-CoV-2"  OR  "2019-nCoV" ) )  AND  ( ( TITLE-ABS-KEY abuse  OR  use*  OR  usage  OR  disorder  OR  depend*  W/4  substance  OR  drug  OR  alcohol  OR  marijuana  OR  benzodiazepine  OR  cannabis  OR  opioid  OR  stimulant  OR  methamphetamine ) )  OR  ( ( TITLE-ABS-KEY ( "mental health"  OR  "well-being"  OR  wellbeing  OR  psychiatr*  OR  psycholog*  OR  neuropsycholog*  OR  neurolog*  OR  anxious*  OR  anxiety  OR  depress*  OR  "post-traumatic"  OR  isolat*  OR  fear*  OR  insomnia  OR  hopeless*  OR  panic*  OR  hysteri*  OR  stress*  OR  insecur*  OR  confine*  OR  confus*  OR  trauma*  OR  irritab*  OR  frustrat*  OR  bored*  OR  anger*  OR  angry  OR  depress*  OR  dysthymi*  OR  bipolar  OR  cyclothymi*  OR  mania  OR  manic  OR  hypomania  OR  gad  OR  agoraphobia  OR  phobia*  OR  ocd  OR  bdd  OR  trichotillomania  OR  ptsd  OR  psychotic  OR  psychos*  OR  schizo*  OR  somati*  OR  somatoform  OR  anorexi*  OR  bulimi*  OR  pyromania  OR  kleptomania  OR  adhd ) )  OR  ( TITLE-ABS-KEY ( disorder*  OR  illness*  W/4  mental  OR  psychological  OR  psychiatric  OR  mood  OR  affective  OR  anxiety  OR  panic  OR  "obsessive-compulsive"  OR  "body dysmorphic"  OR  hoarding  OR  "hair-pulling"  OR  excoriation  OR  "skin-picking"  OR  "post-traumatic"  OR  posttraumatic  OR  "acute stress"  OR  adjustment  OR  delusional  OR  dissociative  OR  conversion  OR  depersonali?ation  OR  dereali?ation  OR  eating  OR  feeding  OR  "oppositional defiant"  OR  conduct  OR  disruptive  OR  "impulse control"  OR  personality  OR  "attention deficit"  OR  motor  OR  neurocognitive  OR  neurodevelopmental  OR  neurotic  OR  paraphilic  OR  psychotic  OR  "substance-related"  OR  trauma  OR  stressor ) ) ) ) )  AND  ( ( TITLE-ABS-KEY ( systematic ) )  AND  ( ( TITLE-ABS-KEY (review*  W/4  systemati*  OR  literature  OR  critical  OR  mapping  OR  scoping  OR  umbrella  OR  rapid  OR  evidence-based ) )  OR  ( TITLE-ABS-KEY ( overview*  OR  meta-analys* ) ) ) ) )  AND  ORIG-LOAD-DATE  AFT  1639377569  AND  ORIG-LOAD-DATE  BEF  1639982367  AND  PUBYEAR  AFT  2019

## S5 Table. Results of corrected covered area analysis

|  | **Overall** | **General population** | **HCW** | **COVID-19 Patients** | **Pregnant/ postpartum** | **Vulnerable populations** | **Young people** |
| --- | --- | --- | --- | --- | --- | --- | --- |
| **Depression** | 1.24% | 1.66% | 3.28% | 8.43% | 16.86% | 0.00% | 3.59% |
| **Anxiety** | 1.33% | 1.98% | 4.00% | 8.43% | 23.88% | 0.00% | 3.28% |
| **Stress** | 3.07% | 3.29% | 6.59% | NA (k=0) | NA (k=1) | NA (k=0) | 14.29% |
| **Psychological distress** | 3.14% | 6.13% | 9.59% | NA (k=0) | NA (k=1) | NA (k=0) | NA (k=0) |
| **PTSD/PTSS** | 2.18% | 4.85% | 3.20% | 4.55% | NA (k=0) | NA (k=0) | NA (k=1) |

## S6 Table. SWiM Table

| SWiM reporting item | Item description | Section in manuscript where item is reported | Other* |
| --- | --- | --- | --- |
| **Methods** | | | |
| 1 Grouping studies for synthesis | 1a) Provide a description of, and rationale for, the groups used in the synthesis (eg, groupings of populations, interventions, outcomes, study design) | Methods: Search strategy  Methods: Data synthesis – Narrative synthesis |  |
|  | 1b) Detail and provide rationale for any changes made subsequent to the protocol in the groups used in the synthesis | N/A |  |
| 2 Describe the standardised metric and transformation methods used | Describe the standardised metric for each outcome. Explain why the metric(s) was chosen and describe any methods used to transform the intervention effects, as reported in the study, to the standardised metric, citing any methodological guidance consulted | Methods: Data synthesis – Narrative synthesis |  |
| 3 Describe the synthesis methods | Describe and justify the methods used to synthesise the effects for each outcome when it was not possible to undertake a meta-analysis of effect estimates | Methods: Data synthesis – Narrative synthesis |  |
| 4 Criteria used to prioritise results for summary and synthesis | Where applicable, provide the criteria used, with supporting justification, to select the particular studies, or a particular study, for the main synthesis or to draw conclusions from the synthesis (eg, based on study design, risk of bias assessments, directness in relation to the review question) | Methods: Data synthesis – Narrative synthesis |  |
| 5 Investigation of heterogeneity in reported effects | State the method(s) used to examine heterogeneity in reported effects when it was not possible to undertake a meta-analysis of effect estimates and its extensions to investigate heterogeneity | Methods: Data synthesis – Narrative synthesis |  |
| 6 Certainty of evidence | Describe the methods used to assess the certainty of the synthesis findings | Methods: Quality Assessment  Methods: Data synthesis – Narrative synthesis |  |
| 7 Data presentation methods | Describe the graphical and tabular methods used to present the effects (eg, tables, forest plots, harvest plots) | Results: Reviews included in narrative synthesis | S8 Table |
|  | Specify key study characteristics (eg, study design, risk of bias) used to order the studies, in the text and any tables or graphs, clearly referencing the studies included |  |  |
| **Results** | | | |
| 8 Reporting results | For each comparison and outcome, provide a description of the synthesised findings and the certainty of the findings. Describe the result in language that is consistent with the question the synthesis addresses, and indicate which studies contribute to the synthesis | Results: Narrative synthesis of mental health impacts | S8 Table |
| **Discussion** | | | |
| 9 Limitations of the synthesis | Report the limitations of the synthesis methods used and/or the groupings used in the synthesis and how these affect the conclusions that can be drawn in relation to the original review question | Discussion: Limitations section |  |

## S7 Table. Reviews excluded from narrative synthesis due to low or critically low quality

| **K = 313** | | |
| --- | --- | --- |
| **First author et al (year)** | **Type of review** | **AMSTAR-2 grade** |
| Abasiyanik, Z., et al. (2022)[1] | SR | Low |
| Abdulla, E. K., et al. (2021)[2] | SR+MA | Critically low |
| Al Moaleem, M. M. (2022)[3] | SR | Critically low |
| Acuff, S. F., et al. (2022)[4] | SR+MA | Low |
| Adibi, A., et al. (2021)[5] | SR+MA | Critically low |
| Ahmad, M. S., et al. (2021)[6] | SR | Critically low |
| Ahmadi Hekmatikar, A. H., et al. (2022)[7] | SR | Low |
| Al Falasi, B., et al. (2021)[8] | SR+MA | Critically low |
| Al Mamun, F., et al. (2021)[9] | SR | Critically low |
| Al Maqbali, M., et al. (2021)[10] | SR+MA | Critically low |
| Alaradi, A., et al. (2021)[11] | SR | Critically low |
| Alimoradi, Z., et al. (2021)[12] | SR+MA | Low |
| Abedi, N. (2020)[13] | SR | Critically low |
| Allan, S. M., et al. (2020)[14] | SR+MA | Critically low |
| Almeida, I. L. L., et al. (2021)[15] | SR | Critically low |
| Almqvist, J., et al. (2020)[16] | SR | Low |
| Alonso-Esteban, Y., et al. (2021)[17] | SR | Critically low |
| Altieri, M., et al. (2022)[18] | MA | Critically low |
| Alzahrani, F., et al. (2022)[19] | SR+MA | Critically low |
| Amanullah, S., et al. (2020)[20] | SR | Critically low |
| Amorós-Reche, V., et al. (2022)[21] | SR | Critically low |
| Araujo, L. A., et al. (2020)[22] | SR | Critically low |
| Arora, T., et al. (2020)[23] | SR+MA | Critically low |
| Aymerich, C., et al. (2022)[24] | SR+MA | Critically low |
| Ayu, N. M. S., et al. (2021)[25] | SR | Critically low |
| Ayubi, E., et al. (2021)[26] | SR+MA | Critically low |
| Badenoch, J. B., et al. (2022)[27] | SR+MA | Critically low |
| Balakrishnan, V., et al. (2022)[28] | SR+MA | Critically low |
| Banerjee, D., et al. (2020)[29] | SR | Critically low |
| Bareeqa, S. B., et al. (2020)[30] | SR+MA | Critically low |
| Basnayake, O., et al. (2021)[31] | SR | Critically low |
| Batista, P., et al. (2021)[32] | SR | Critically low |
| Batra, K., et al. (2021)[33] | MA | Low |
| Behrmann, J. T., et al. (2021)[34] | SR | Low |
| Bekele, F., et al. (2021, SAGE Open Med.)[35] | SR | Critically low |
| Bekele, F., et al. (2021, Ann. Med. Surg.)[36] | SR | Critically low |
| Berger, E., et al. (2021)[37] | SR | Critically low |
| Billings, J., et al. (2021)[38] | SR | Critically low |
| Blasco-Belled, A., et al. (2022)[39] | MA | Low |
| Boden, M., et al. (2021)[40] | MA | Critically low |
| Bourmistrova, N. W., et al. (2022)[41] | SR | Low |
| Budiman, A. A., et al. (2021)[42] | SR | Critically low |
| Buecker, Susanne, et al. (2022)[43] | SR | Critically low |
| Bueno-Notivol, J., et al. (2021)[44] | MA | Low |
| Busch, I. M., et al. (2021)[45] | SR+MA | Critically low |
| Bussières, E. L., et al. (2021)[46] | MA | Critically low |
| C Fong, V., et al. (2020)[47] | SR | Critically low |
| Cabarkapa, S., et al. (2020)[48] | SR | Critically low |
| Caffieri, A., et al. (2021)[49] | SR | Critically low |
| Caponnetto, P., et al. (2021)[50] | SR | Critically low |
| Carbone, E. A., et al. (2021)[51] | SR | Critically low |
| Carmassi, C., et al. (2020)[52] | SR | Critically low |
| Caruso, R., et al. (2021)[53] | SR+MA | Critically low |
| Cavicchioli, M., et al. (2021)[54] | SR | Low |
| Cenat, J. M., et al. (2021)[55] | SR+MA | Critically low |
| Chaabane, S., et al. (2021)[56] | SR | Critically low |
| Chai, J., et al. (2021)[57] | SR+MA | Critically low |
| Chang, J. J., et al. (2021)[58] | MA | Critically low |
| Chawla, N., et al. (2021)[59] | SR | Critically low |
| Chekole, Y. A., et al. (2021)[60] | SR+MA | Low |
| Chen, J., et al. (2021)[61] | SR+MA | Low |
| Cheng, C. K. T., et al. (2022)[62] | SR+MA | Low |
| Chigwedere, O. C., et al. (2021)[63] | SR | Low |
| Ching, S. M., et al. (2021)[64] | SR+MA | Critically low |
| Chirico, F., et al. (2021)[65] | SR | Critically low |
| Chtourou, H., et al. (2020)[66] | SR | Critically low |
| Chu, I. Y., et al. (2020)[67] | SR | Low |
| Ciuffreda, G., et al. (2021)[68] | SR | Critically low |
| Crocamo, C., et al. (2021)[69] | SR+MA | Critically low |
| Cunning, C., et al. (2022)[70] | SR | Critically low |
| D'Ettorre, G., et al. (2021)[71] | SR | Critically low |
| D'Ettorre, G., et al. (2020)[72] | SR | Critically low |
| da Silva, F. C. T., et al. (2021, Prog. Neuropsychopharmacol. Biol., vol 110)[73] | SR | Critically low |
| da Silva, F. C. T., et al. (2021, Prog. Neuropsychopharmacol. Biol., vol 104)[74] | SR+MA | Critically low |
| da Silva, M. L., et al. (2020)[75] | MA | Critically low |
| De Brier, N., et al. (2020)[76] | SR | Low |
| Della Monica, A., et al. (2021)[77] | SR | Critically low |
| Dellazizzo, L., et al. (2021)[78] | SR | Critically low |
| Demissie, D. B., et al. (2021)[79] | SR+MA | Critically low |
| Deng, J., et al. (2021)[80] | SR+MA | Critically low |
| Dettmann, L. M., et al. (2022)[81] | SR+MA | Low |
| Dhada, S., et al. (2021)[82] | SR | Low |
| Dong, F. (2021, Front. Psychol.)[83] | SR+MA | Low |
| Dong, F. (2021, J. Affect. Disord)[84] | SR+MA | Critically low |
| Dorri, M., et al. (2021)[85] | SR+MA | Low |
| dos Santos, E. R. R., et al. (2021)[86] | SR | Critically low |
| Dragioti, E., et al. (2021)[87] | MA | Critically low |
| Dube, J. P., et al. (2021)[88] | MA | Critically low |
| Dullius, W. R., et al. (2021)[89] | SR | Critically low |
| Dutta, A., et al. (2021)[90] | SR+MA | Low |
| Elharake, J. A., et al. (2022)[91] | SR | Critically low |
| Esposito, C., et al. (2021)[92] | SR | Critically low |
| Fan, F. C., et al. (2021)[93] | MA | Critically low |
| Fan, S., et al. (2020)[94] | SR+MA | Critically low |
| Farooq, S., et al. (2021)[95] | SR+MA | Low |
| Fleischmann, E., et al. (2021)[96] | SR | Critically low |
| d’Ettorre, G., et al. (2022)[97] | SR | Critically low |
| Gentry, S. V., et al. (2022)[98] | SR | Critically low |
| Ghazanfarpour, M., et al. (2021)[99] | MA | Critically low |
| Gianfredi, V., et al. (2021)[100] | SR | Critically low |
| Gibson, B., et al. (2021)[101] | SR | Low |
| Gray, K. L., et al. (2021)[102] | SR | Critically low |
| Groff, D., et al. (2021)[103] | SR | Low |
| Gross, J. V., et al. (2021)[104] | SR | Critically low |
| Guo, S., et al. (2021)[105] | SR+MA | Critically low |
| Guzick, A. G., et al. (2021)[106] | SR | Critically low |
| Han, Q., et al. (2022)[107] | SR+MA | Low |
| Hannemann, J., et al. (2022)[108] | SR | Low |
| Hao, Q., et al. (2021)[109] | SR+MA | Low |
| Hekmat, A., et al. (2021)[110] | SR | Critically low |
| Hesary, F. B., et al. (2021)[111] | SR | Critically low |
| Hessami, K., et al. (2020)[112] | SR+MA | Critically low |
| Hill, J. E., et al. (2022)[113] | SR+MA | Critically low |
| Hintermeier, M., et al. (2021)[114] | SR | Low |
| Hosen, I., et al. (2021)[115] | SR+MA | Critically low |
| Hossain, M. M., et al. (2021)[116] | SR+MA | Low |
| Hu, N., et al. (2022)[117] | MA | Critically low |
| Huerta-Gonzalez, S., et al. (2021)[118] | SR | Critically low |
| Hugelius, K., et al. (2021)[119] | SR | Critically low |
| Imran, N., et al. (2020)[120] | SR | Critically low |
| Imtiaz, S., et al. (2021)[121] | SR | Critically low |
| Iyengar, U., et al. (2021)[122] | SR | Critically low |
| Jammu, A. S., et al. (2020)[123] | SR | Critically low |
| Jennings, G., et al. (2021)[124] | SR | Critically low |
| Jesline, J., et al. (2021)[125] | SR | Critically low |
| Johns, G., et al. (2022)[126] | SR+MA | Low |
| Jones, E. A. K., et al. (2021)[127] | SR | Critically low |
| Jothishanmugam, A., et al. (2020)[128] | SR | Critically low |
| Jurecka, A., et al. (2021)[129] | SR | Low |
| Kahil, K., et al. (2021)[130] | SR | Low |
| Keskin, S., et al. (2021)[131] | SR | Critically low |
| Khaffaf, E. S., et al. (2021)[132] | SR | Critically low |
| Khoundabi, B., et al. (2021)[133] | SR | Critically low |
| Khraisat, B. R., et al. (2022)[134] | MA | Critically low |
| Khraisat, B., et al. (2021)[135] | MA | Critically low |
| Killikelly, C., et al. (2021)[136] | SR | Critically low |
| Kirubarajan, A., et al. (2021)[137] | SR | Low |
| Kisely, S., et al. (2020)[138] | MA | Critically low |
| Koontalay, A., et al. (2021)[139] | SR | Critically low |
| Krishnamoorthy, Y., et al. (2020)[140] | SR+MA | Low |
| Kunz, M., et al. (2021)[141] | SR | Critically low |
| Kunzler, A. M., et al. (2021)[142] | SR+MA | Critically low |
| Kuroda, N., et al. (2021)[143] | SR+MA | Critically low |
| Kusumawati, M. W., et al. (2022)[144] | SR | Critically low |
| Lasheras, I., et al. (2020)[145] | SR+MA | Critically low |
| Lee, H. J., et al. (2021)[146] | SR | Critically low |
| Lee, K. W., et al. (2022)[147] | SR+MA | Critically low |
| Lehmann, J., et al. (2021)[148] | SR | Critically low |
| Li, W., et al. (2021)[149] | SR+MA | Low |
| Li, W., et al. (2022)[150] | SR+MA | Critically low |
| Li, Y., et al. (2021, PLoS ONE)[151] | SR+MA | Low |
| Lieneck, C., et al. (2021)[152] | SR | Critically low |
| Lin, C. T., et al. (2021)[153] | SR | Critically low |
| Lin, C. Y., et al. (2020)[154] | SR+MA | Critically low |
| Linardon, J., et al. (2022)[155] | SR | Critically low |
| Liyanage, S., et al. (2021)[156] | SR+MA | Critically low |
| Luo, F., et al. (2021)[157] | SR+MA | Critically low |
| Luo, M., et al. (2020)[158] | SR+MA | Critically low |
| Mamun, M. A. (2021)[159] | SR | Critically low |
| Luo, W., et al. (2021)[160] | SR+MA | Critically low |
| Luo, Y., et al. (2020)[161] | SR | Low |
| Luo, Y., et al. (2022)[162] | MA | Critically low |
| Ma, L., et al. (2021)[163] | SR+MA | Low |
| Mahmud, S., et al. (2021)[164] | SR+MA | Low |
| Mahmud, S., et al. (2022)[165] | SR+MA | Critically low |
| Malik, P., et al. (2022)[166] | SR+MA | Critically low |
| Marciano, L., et al. (2022)[167] | SR+MA | Critically low |
| Marconcin, P., et al. (2022)[168] | SR | Low |
| Marvaldi, M., et al. (2021)[169] | SR+MA | Critically low |
| Matondang, E. R. S., et al. (2021)[170] | SR | Critically low |
| McGowan, V. J., et al. (2021)[171] | SR | Low |
| Meherali, S., et al. (2021)[172] | SR | Low |
| Min, S., et al. (2021)[173] | SR+MA | Critically low |
| Miniati, M., et al. (2021)[174] | SR | Critically low |
| Mohamed, N., et al. (2021)[175] | SR | Critically low |
| Momenimovahed, Z., et al. (2021)[176] | SR | Critically low |
| Monteleone, A. M., et al. (2021)[177] | SR | Critically low |
| Morrish, N., et al. (2021)[178] | SR | Critically low |
| Mousavizadeh, S. N., et al. (2021)[179] | SR | Critically low |
| Muller, A. E., et al. (2020)[180] | SR | Low |
| Mulyadi, M., et al. (2021)[181] | SR+MA | Critically low |
| Munro, A., et al. (2021)[182] | SR | Critically low |
| Nagarajan, R., et al. (2022)[183] | SR+MA | Critically low |
| Nagi, R., et al. (2021)[184] | SR | Critically low |
| Nam, S. H., et al. (2021)[185] | SR+MA | Critically low |
| Nasserie, T., et al. (2021)[186] | SR | Critically low |
| Nearchou, F., et al. (2020)[187] | SR | Low |
| Necho, M., et al. (2021)[188] | SR+MA | Critically low |
| Neelam, K., et al. (2020)[189] | SR+MA | Critically low |
| Nobari, H., et al. (2021)[190] | SR | Critically low |
| Norhayati, M. N., et al. (2021)[191] | SR+MA | Low |
| Nowrouzi-Kia, B., et al. (2021)[192] | SR+MA | Critically low |
| Nursalam, N., et al. (2020)[193] | SR | Low |
| Okpua, N. C., et al. (2021)[194] | SR | Critically low |
| Olashore, A., et al. (2021)[195] | SR | Critically low |
| Olaya, B., et al. (2021)[196] | SR+MA | Critically low |
| Oliveira Carvalho, P., et al. (2021)[197] | SR+MA | Critically low |
| Oliveira, J. M. D., et al. (2022)[198] | SR | Low |
| Ozamiz-Etxebarria, N., et al. (2021)[199] | SR+MA | Critically low |
| Ozguc, S., et al. (2021)[200] | SR+MA | Critically low |
| Pai, N., et al. (2021)[201] | SR | Critically low |
| Panchal, U., et al. (2021)[202] | SR | Low |
| Panda, P. K., et al. (2020)[203] | SR+MA | Low |
| Pappa, S., et al. (2020)[204] | SR+MA | Critically low |
| Pappa, S., et al. (2021)[205] | SR+MA | Low |
| Pashazadeh Kan, F., et al. (2021)[206] | SR+MA | Critically low |
| Phiri, P., et al. (2021)[207] | SR+MA | Critically low |
| Pian, W., et al. (2021)[208] | SR | Critically low |
| Raihan, M. M. H. (2021)[209] | SR | Critically low |
| Porter, B., et al. (2021)[210] | SR | Low |
| Premraj, L., et al. (2022)[211] | MA | Critically low |
| Purnama, A., et al. (2021)[212] | SR | Critically low |
| Qiu, D., et al. (2021, Eur. Psychiatry)[213] | MA | Low |
| Qiu, D., et al. (2021, Front. Psychol.)[214] | SR+MA | Critically low |
| Racine, N., et al. (2021)[215] | MA | Critically low |
| Raoofi, S., et al. (2021)[216] | SR+MA | Critically low |
| Rawat, D., et al. (2021)[217] | SR | Critically low |
| Ren, X., et al. (2020)[218] | MA | Critically low |
| Rezaei-Hachesu, V., et al. (2022)[219] | SR+MA | Critically low |
| Rezaei, S., et al. (2022)[220] | SR+MA | Critically low |
| Roberts, A., et al. (2021)[221] | SR | Critically low |
| Robinson, E., et al. (2022)[222] | SR+MA | Critically low |
| Rocha, Y. M., et al. (2021)[223] | SR | Critically low |
| Rodriguez-Fernandez, P., et al. (2021)[224] | SR | Critically low |
| Rogers, J. P., et al. (2020)[225] | SR+MA | Critically low |
| Rogers, J. P., et al. (2021)[226] | SR+MA | Low |
| Ruksakulpiwat, S., et al. (2021)[227] | SR | Critically low |
| Runacres, A., et al. (2021)[228] | SR+MA | Critically low |
| Russo, G., et al. (2021)[229] | SR | Critically low |
| Saeed, H., et al. (2022)[230] | SR | Critically low |
| Safi-Keykaleh, M., et al. (2022)[231] | SR+MA | Critically low |
| Salamanna, F., et al. (2021)[232] | SR | Critically low |
| Salari, N., et al. (2020, Glob. Health)[233] | SR+MA | Low |
| Salari, N., et al. (2020, Hum. Resour. Health)[234] | SR+MA | Critically low |
| Salazar de Pablo, G., et al. (2020)[235] | SR+MA | Critically low |
| Salehi, M., et al. (2021)[236] | SR+MA | Critically low |
| Samji, H., et al. (2021)[237] | SR | Critically low |
| Sanghera, J., et al. (2020)[238] | SR | Critically low |
| Santabarbara, J., et al. (2020, July. Prog. Neuropsychopharmacol. Biol. Psychiatry)[239] | MA | Low |
| Santabarbara, J., et al. (2021, Medicina [Kaunas])[240] | MA | Critically low |
| Santabarbara, J., et al. (2021, Prog. Neuropsychopharmacol. Biol. Psychiatry)[241] | SR+MA | Critically low |
| Santomauro, D.F., et al. (2021)[242] | SR+MA | Critically low |
| Saragih, I. D., et al. (2021)[243] | SR+MA | Low |
| Schneider, J., et al. (2022)[244] | SR | Low |
| Schou, T. M., et al. (2021)[245] | SR | Critically low |
| Schubert, M., et al. (2021)[246] | SR+MA | Critically low |
| Scortegagna, Silvana Alba, et al. (2021)[247] | SR | Critically low |
| Serrano-Ripoll, M. J., et al. (2020)[248] | SR+MA | Critically low |
| SeyedAlinaghi, S., et al. (2021)[249] | SR | Critically low |
| Shankar, A., et al. (2021)[250] | SR | Critically low |
| Sheraton, M., et al. (2020)[251] | SR+MA | Critically low |
| Shorey, S. Y., et al. (2021)[252] | SR+MA | Critically low |
| Shukla, J., et al. (2021)[253] | SR | Critically low |
| Sideli, L., et al. (2021)[254] | SR+MA | Low |
| Silva, D. F. O., et al. (2021)[255] | SR | Critically low |
| Simonetti, A., et al. (2020)[256] | SR | Critically low |
| Simsir, Z., et al. (2021)[257] | MA | Critically low |
| Singh, R. K., et al. (2021)[258] | SR+MA | Critically low |
| Slusarska, B., et al. (2022)[259] | SR+MA | Critically low |
| Smith, C. M., et al. (2021)[260] | SR | Critically low |
| Soklaridis, S., et al. (2020)[261] | SR | Critically low |
| Solehati, T., et al. (2021)[262] | SR | Critically low |
| Soltani, S., et al. (2021)[263] | SR+MA | Low |
| Soysal, P., et al. (2022)[264] | SR+MA | Low |
| Sterina, E., et al. (2021)[265] | SR | Critically low |
| Strasser, M. A., et al. (2022)[266] | SR | Low |
| Suarez-Gonzalez, A., et al. (2021)[267] | SR | Low |
| Sun, F., et al. (2020)[268] | MA | Critically low |
| Sun, P., et al. (2021)[269] | SR+MA | Critically low |
| Tashakori-Miyanroudi, M., et al. (2021)[270] | SR | Low |
| Thakur, B., et al. (2021)[271] | SR+MA | Critically low |
| Thatrimontrichai, A., et al. (2021)[272] | SR | Critically low |
| Tibubos, A. N., et al. (2021)[273] | SR | Critically low |
| Tomfohr-Madsen, L. M., et al. (2021)[274] | MA | Critically low |
| Troglio da Silva, F. C., et al. (2021)[275] | SR | Critically low |
| Uphoff, E. P., et al. (2021)[276] | SR | Critically low |
| Usmani, S., et al. (2021)[277] | SR | Critically low |
| van Reekum, E. A., et al. (2021)[278] | SR | Low |
| Vanderlind, W. M., et al. (2021)[279] | SR | Critically low |
| Varghese, A., et al. (2021)[280] | SR+MA | Low |
| Vescovi, Gabriela, et al. (2021)[281] | SR | Critically low |
| Vindegaard, N., et al. (2020)[282] | SR | Critically low |
| Violant-Holz, V., et al. (2020)[283] | SR | Critically low |
| Vizheh, M., et al. (2020)[284] | SR | Low |
| Wang, C., et al. (2021)[285] | SR+MA | Critically low |
| Wang, F., et al. (2022)[286] | SR+MA | Low |
| Wang, Y., et al. (2020)[287] | SR+MA | Low |
| Wang, Z., et al. (2021)[288] | SR | Critically low |
| Whear, R., et al. (2021)[289] | SR | Low |
| Willi, S., et al. (2021)[290] | SR | Low |
| Wolf, S., et al. (2021)[291] | SR | Critically low |
| Wu, L., et al. (2021)[292] | SR | Critically low |
| Wu, T., et al. (2021)[293] | SR+MA | Critically low |
| Wu, Y., et al. (2021)[294] | SR | Critically low |
| Xiong, Jiaqi, et al. (2020)[295] | SR | Critically low |
| Xiong, N., et al. (2022)[296] | SR+MA | Critically low |
| Xu, H., et al. (2021)[297] | SR | Critically low |
| Yaghoubi, M., et al. (2022)[298] | SR | Low |
| Yan, H., et al. (2020)[299] | SR+MA | Critically low |
| Yan, H., et al. (2021)[300] | SR+MA | Low |
| Yan, Y., et al. (2022)[301] | SR+MA | Critically low |
| Yilmaz, B., et al. (2021)[302] | SR | Critically low |
| Yuan, Kai, et al. (2021)[303] | SR+MA | Low |
| Yunitri, N., et al. (2021)[304] | MA | Low |
| Zarghami, A., et al. (2022)[305] | SR | Low |
| Zhang, H., et al. (2022)[306] | SR | Critically low |
| Zhang, L., et al. (2021)[307] | SR+MA | Critically low |
| Zhang, S. X., et al. (2021)[308] | SR | Critically low |
| Zhang, S. X., et al. (2022, Int. J. Environ. Health Res)[309] | SR+MA | Low |
| Zhang, Y., et al. (2021)[310] | MA | Critically low |
| Zhao, Y. J., et al. (2021)[311] | SR+MA | Critically low |
| Zhou, Y., et al. (2021)[312] | SR+MA | Critically low |
| Zhu, J., et al. (2021)[313] | MA | Critically low |

## S8 Table. Characteristics of reviews that received a moderate AMSTAR grade

| **K = 25** | | | | | | | |
| --- | --- | --- | --- | --- | --- | --- | --- |
| **Review** | **Search window end date** | **Population of interest** | **K included studies** | **Study country distribution** | **N included participants** | **Age and sex or gender distribution of pooled sample** | **Study types** |
| Deng, J., Zhou, F., Hou, W., et al (2021). Annals of the New York Academy of Sciences[314] | 18/8/2020 | COVID-19 Patients | 31 | China (27)  Italy (1)  Ecuador (1)  Turkey (1)  Iran (1) | 5,153 | Age: NR  Sex: 49% male | Cross-sectional (28)  Single-arm cohort (3) |
| Octavius, G. S.; Silviani, F. R.; Lesmandjaja, A.; Angelina; Juliansen, A. (2020). Middle East Current Psychiatry (Cairo)[315] | 7/7/2020 | Adolescents | 4 | China (2)  USA (1)  Turkey (1) | 23,601 | Age range: 12 - 18 years  Sex: NR | Cross-sectional (3)  Prospective cohort (1) |
| Hope, C.; Reilly, J. J.; Griffiths, G.; Lund, J.; Humes, D. (2021). Techniques in Coloproctology[316] | 31/08/2020 | HCWs (Surgical trainees and program directors) | 11 | France (1)  India (1)  United States (6)  Pakistan (1)  Belgium (1)  Multi-country (1) | NR | NR | Cross-sectional (11) |
| Schneider, J.; Talamonti, D.; Gibson, B.; Forshaw, M. (2021). Journal of Health Psychology[317] | 7/6/2020 | HCWs | 12 | Italy (1)  China (9)  Israel (1)  Multi-country (1) | 19,640 | Age range: 18 – 79 years  Sex: 71% female | Cross-sectional (12) |
| Chmielewska, B.; Barratt, I.; Townsend, R.; Kalafat, E.; van der Meulen, J.; Gurol-Urganci, I.; O'Brien, P.; Morris, E.; Draycott, T.; Thangaratinam, S.; Le Doare, K.; Ladhani, S.; von Dadelszen, P.; Magee, L.; Khalil, A. (2021). Lancet Global Health[318] | 8/1/2021 | Pregnant and perinatal people | 40 | Turkey (1)  Netherlands (1)  United States (7)  Canada (1)  United Kingdom (3)  Botswana (1)  Italy (4)  India (3)  China (4)  Denmark (1)  Hong Kong (1)  Israel (5)  Japan (2)  Nepal (1)  Mexico (1)  Ireland (3)  Brazil (1) | 2,909,869  Exposed to COVID-19 = 402,678 (included in 2,909,869) | Age: NR  Sex: NR – 100% able to become pregnant (born female) | Historical cohort study design (40) |
| Sirois, F. M.; Owens, J. (2021). Frontiers in Psychiatry[319] | 15/11/2020 | HCWs | 107 | Kurdistan (1)  China (38)  Turkey (8)  Oman (2)  Egypt (2)  Pakistan (3)  Italy (9)  Greece (2)  Saudi Arabia (1)  France (1)  Singapore (1)  Brazil (1)  Spain (7)  United States (4)  Australia (2)  Libya (1)  Malaysia (1)  Argentina (1)  Iran (2)  Mali (1)  Jordan (1)  Russia (1)  Serbia (1)  South Ethiopia (1)  India (5)  Multi-country (6) | 120,711 | NR | Cross-sectional (107) |
| John, A.; Okolie, C.; Eyles, E.; Webb, R. T.; Schmidt, L.; McGuiness, L. A.; Olorisade, B. K.; Arensman, E.; Hawton, K.; Kapur, N.; Moran, P.; O'Connor, R. C.; O'Neill, S.; Higgins, J. P. T.; Gunnell, D. (2021). F1000 Research[320] | 6/7/2020 | General population, with examination of sub-populations including people with pre-existing mental illness, pregnant people, people who have contracted COVID-19 | 29 articles  28 independent studies | United States (6)  China (2)  India (2)  Australia (1)  Bangladesh (1)  Canada (1)  Germany (1)  Greece (1)  Pakistan (1)  Spain (1)  France (1)  Switzerland (1)  Multi-country (6) | 33,345 | NR | Cross-sectional (13, with 12 independent samples)  Case series (8)  Modelling studies (5)  Service utilisation studies (3) |
| Eyles, E., Moran, P., Okolie, C., Dekel, D., Macleod-Hall, C., & Webb, R. et al. (2021). Journal of Affective Disorder Reports[321] | 31/3/2021 | HCW (frontline, non-frontline) | 10 | Belgium (1)  China (4)  Mexico (1)  Malaysia (1)  Colombia (1)  United States (1)  Spain (1) | 40,228 | NR | Cross-sectional (8)  Cross-sectional with planned follow-ups (2) |
| Lee, Y., Lui, L., Chen-Li, D., Liao, Y., Mansur, R., & Brietzke, E. et al. (2021). Journal of Affective Disorders[322] | 16/9/2020 | General population | 114 | Albania (1)  Austria (1)  Bangladesh (1)  Bosnia and Herzegovina (1)  Brazil (1)  Canada (3)  China (41)  Ecuador (1)  France (1)  Germany (4)  Greece (3)  Hong Kong (1)  India (2)  Iran (3)  Ireland (3)  Israel (3)  Italy (4)  Japan (1)  Jordan (1)  Kenya (1)  Mexico (1)  Nepal (1)  Norway (1)  Pakistan (1)  Poland (1)  Saudi Arabia (1)  South Korea (4)  Spain (3)  Sweden (2)  Switzerland (2)  Turkey (1)  United Kingdom (5)  United States (20)  Vietnam (1) | 640,037 | NR | NR |
| Li, Y.,  Wang, A.,  Wu, Y.,  Han, N.,  Huang, H., (2021). Frontiers in Psychology[323] | October 2020 | College students | 27 | China (15)  France (1)  United States (4)  Jordan (1)  Spain (1)  Bangladesh (1)  Lebanon (1)  Switzerland (1)  Israel (1)  Multi-country (1) | 706,415 | Age: NR   59.7% male  40.3% female  0.01% other gender  (Excluding one study that did not report gender) | Cross-sectional (27) |
| Muehlschlegel, P., Parkinson, E., Chan, R., Arden, M., & Armitage, C. (2021). Journal of Global Health[324] | 12/1/2021 | No specific population – included original studies that spanned any population | 15 | United Kingdom (1)  United States (2)  Vietnam (1)  Italy (1)  India (2)  China (7)  Austria (1) | 38,112 | NR | Cross-sectional (8)  Longitudinal (7) |
| Nochaiwong, S., Ruengorn, C., Thavorn, K., Hutton, B., Awiphan, R., & Phosuya, C. et al. (2021). Scientific Reports[325] | 16/6/2020 | General population | 107 | Nigeria (1)  South Africa (1)  Brazil (4)  Mexico (1)  United States (7)  Bangladesh (3)  India (5)  Nepal (1)  Thailand (1)  Germany (1)  Greece (1)  Ireland (1)  Italy (1)  Norway (1)  Portugal (1)  Spain (6)  Sweden (1)  Turkey (2)  United Kingdom (7)  Egypt (1)  Iran (2)  Jordan (2)  Pakistan (1)  Saudi Arabia (2)  Tunisia (1)  United Arab Emirates (2)  Australia (4)  China- including Hong Kong, Macau, Taiwan (36)  Japan (1)  Malaysia (1)  New Zealand (1)  Vietnam (1) | 398,771 | Females: 60.9% (range = 16.0 – 51.6)  Age:  33.5 ± 9.5 years | Cross-sectional (96)  Not extracted (11) |
| Renaud-Charest, O., Lui, L., Eskander, S., Ceban, F., Ho, R., & Di Vincenzo, J. et al. (2021). Journal of Psychiatric Research[326] | 5/6/2021 | People who have had COVID-19 | 8 | Italy (2)  Spain (1)  Austria (1)  France (1)  Germany (1)  United States (1)  The Netherlands (1) | 1,088 | NR | Observational (8) |
| Schmidt, R., Genois, R., Jin, J., Vigo, D., Rehm, J., & Rush, B. (2021). Drug and Alcohol Dependence[327] | March 2021 | No specific population – included original studies that spanned any population | 53 | China (1)  Argentina (1)  Norway (1)  Poland (6)  United States (11)  Australia (6)  France (3)  Croatia (1)  Canada (4)  New Zealand (2)  United Kingdom (3)  Germany (1)  Lithuania (1)  Spain (3)  Italy (2)  Belgium (1)  Czech Republic (1)  Finland (1)  Multi-country (4) | 203,889 | NR | Cross-sectional (49)  Longitudinal (4) |
| Soto-Cámara, R., García-Santa-Basilia, N., Onrubia-Baticón, H., Cárdaba-García, R., Jiménez-Alegre, J., & Reques-Marugán, A. et al. (2021). Journal of Clinical Medicine[328] | November 2021 | HCW | 20 | Italy (6)  Germany (2)  Spain (2)  Turkey (2)  Belgium (1)  India (1)  Iran (1)  Pakistan (1)  Poland (1)  Russia (1)  United States (1)  Multi-country (1) | 15,396 | NR | Cross-sectional (16)  Qualitative with phenomenological focus (1)  Qualitative with thematic analysis (2)  Qualitative ethnographic with an interpretative focus (1) |
| Zhang, H., Li, W., Li, H., Zhang, C., Luo, J., & Zhu, Y. et al. (2021). General Psychiatry[329] | 8/5/2020 | HCW | 26 | China (26) | 22,062 | NR | Cross-sectional (20) |
| Alimoradi, Z., Ohayon, M., Griffiths, M., Lin, C., & Pakpour, A. (2022). Bjpsych Open[330] | June 2021 | No specific population – included original studies that spanned any population | 91 | Australia (2)  Bangladesh (5)  Brazil (1)  Canada (1)  China (5)  Ecuador (1)  Egypt (2)  Germany (1)  Greece (4)  Hong Kong (1)  India (3)  Iran (6)  Israel (2)  Italy (4)  Japan (4)  Jordan (1)  Korea (1)  Lebanon (1)  Malaysia (2)  Mexico (1)  Pakistan (6)  Paraguay (1)  Peru (1)  Philippines (2)  Poland (3)  Romania (1)  Russia (2)  Saudi Arabia (4)  Singapore (1)  Spain (3)  Taiwan (1)  The Netherlands (1)  Turkey (10)  United Arab Emirates (1)  United Kingdom (4)  United States (1)  Vietnam (1) | 88,320 | Sex: Female = 60.66%  Mean age: 38.88 years | Cross-sectional (89)  Longitudinal (1)  Time-lagged (1) |
| Jefferson, L., Golder, S., Heathcote, C., Avila, A., Dale, V., & Essex, H. et al. (2022). British Journal of General Practice[331] | 3 June 2021 | HCW (General Practitioners) | 31 | Italy (4)  China (4)  Singapore (3)  France (2)  Colombia (2)  United Kingdom (2)  United States (2)  Australia (1)  Croatia (1)  Indonesia (1)  Jordan (1)  Oman (1)  Portugal (1)  Romania (1)  Saudi Arabia (1)  Spain (1)  Turkey (1)  Multi-country (2) | 10,821 | Sex: 41.3% male  Mean age: 42.4 years | Cross-sectional (25)  Qualitative (5)  Mixed survey and qualitative study (1) |
| Racine, N., Eirich, R., Cooke, J., Zhu, J., Pador, P., Dunnewold, N., & Madigan, S. (2021). Infant Mental Health Journal[332] | 3 March 2021 | Mothers of young children | 18 | Bangladesh (1)  Brazil (1)  Canada (1)  China (1)  Indonesia (1)  Israel (1)  Italy (5)  Japan (1)  Serbia (1)  Spain (1)  Turkey (1)  United States (1)  United Kingdom (2) | 8,981 | Sex: Females (100%) | Cross-sectional (18) |
| Veazie, S., Lafavor, B., Vela, K., Young, S., Sayer, N., Carlson, K., & O'Neil, M. (2022). Journal of Affective Disorders Reports[333] | March 2021 | People who have had COVID-19 | 19 (19 articles, from 17 studies) | Single country  Bangladesh (1)  China (6)  Iran (2)  Italy (2)  Norway (1)  Turkey (2)  United Kingdom (2)  United States (3) | 319,597 | Sex: Female percentage 8-65%  Age: mean 35-60 years | Cross-sectional (13)  Prospective cohort (2)  Retrospective cohort (2)  NOTE: There were 17 study designs, and 19 papers. |
| Viner, R., Russell, S., Saulle, R., Croker, H., Stansfield, C., & Packer, J. et al. (2022). JAMA Pediatrics[334] | 1 September 2020 | Children and adolescents | 36 | Japan (1)  United Kingdom (7)  United States (1)  China (7)  Italy (3)  Turkey (1)  Ireland (1)  India (1)  Canada (1)  Brazil (1)  Bangladesh (1) | 97,809 | Age: Range 0 – 19 years was the criterion but they did not specify | Cohort study (5- one used a parallel comparison group, four used an external historical retrospective comparison group)  Uncontrolled pre-post studies (9- eight included pre- and post-exposure measurements, one was a repeated measures time-series study)  Cross-sectional (21)  Modelling study (1) |
| Wan Mohd Yunus, W., Kauhanen, L., Sourander, A., Brown, J., Peltonen, K., & Mishina, K. et al. (2022). Child and Adolescent Psychiatry and Mental Health[335] | 22 March 2021 | Children and adolescents | 18 | United States (6)  United Kingdom (2)  Japan (2)  Spain (1)  Italy (1)  New Zealand (1)  Australia (1)  Canada (1)  France (1)  Israel (1)  Multi-country (1) | NR (reported presentations to the ER before and during the pandemic) | Age range = 0 – 24 years  Sex: NR | Did not specify as all were records collected from electronic, administrative, or national databases. |
| Liu, C., Pan, W., Li, L., Li, B., Ren, Y., & Ma, X. (2021). Journal of Psychosomatic Research[336] | 26 Dec 2020 | COVID-19 Patients | 22 | China (14)  South Korea (1)  India (1)  Ecuador (1)  Jordan (1)  Turkey (1)  Italy (1)  Iran (2) | 4,318 | NR | Cross-sectional (19)  Single-arm cohort (3) |
| Kilian, C., O'Donnell, A., Potapova, N., López‐Pelayo, H., Schulte, B., & Miquel, L. et al. (2022). Drug and Alcohol Review[337] | 31 Sept 2021 | General population | 72 | Austria (2)  Belgium (3)  France (4)  Germany (4)  Netherlands (4)  Denmark (1)  Finland (2)  Ireland (3)  Lithuania (1)  Norway (3)  Sweden (3)  United Kingdom (7)  Croatia (1)  Cyprus (1)  Greece (2)  Slovenia (1)  Spain (3)  Georgia (1)  Poland (3)  Multi-country (6) | 277,157 | NR | Cross-sectional (71)  Longitudinal (1) |
| Zhang, S., Miller, S., Xu, W., Yin, A., Chen, B., & Delios, A. et al. (2022). European Journal of Traumatology[338] | 6/2/2021 | General population, HCW and students | 21 | Albania (2) Bosnia and Herzegovina (1) Bulgaria (1) Croatia (3) Czech Republic (1) Kosovo (1) Poland (4) Russia (3) Serbia (4) Ukraine (1) | 23,253 | Sex: 68.8% (range = 0 – 100) | Cross-sectional (20)  Cohort (1) |

## S9 Table. Meta-analyses included in meta-review

| **K = 83** | | | |
| --- | --- | --- | --- |
| **First author et al (year)** | **AMSTAR-2 grade** | **Included in pooled prevalence meta-review** | **Included in comparison to pre-COVID-19 meta-review** |
| Abdulla, E. K., et al. (2021)[2] | Critically low | Y | N |
| Adibi, A., et al. (2021)[5] | Critically low | Y | N |
| Al Maqbali, M., et al. (2021)[10] | Critically low | Y | N |
| Altieri, M., et al. (2022)[18] | Critically low | N | Y |
| Aymerich, C., et al. (2022)[24] | Critically low | Y | N |
| Badenoch, J. B., et al. (2022)[27] | Critically low | Y | N |
| Balakrishnan, V., et al. (2022)[28] | Critically low | Y | N |
| Bareeqa, S. B., et al. (2020)[30] | Critically low | Y | N |
| Batra, K., et al. (2021)[33] | Low | Y | N |
| Bussières, E. L., et al. (2021)[46] | Critically low | N | Y |
| Cenat, J. M., et al. (2021)[55] | Critically low | Y - Supplementary model only | N |
| Chai, J., et al. (2021)[57] | Critically low | Y | N |
| Chang, J. J., et al. (2021)[58] | Critically low | Y | N |
| Chekole, Y. A., et al. (2021)[60] | Low | Y - Supplementary model only | N |
| Chen, J., et al. (2021)[61] | Low | Y - Supplementary model only | N |
| Ching, S. M., et al. (2021)[64] | Critically low | Y | N |
| Chmielewska, B., et al. (2021)[318] | Moderate | N | Y |
| Crocamo, C., et al. (2021)[69] | Critically low | Y | N |
| Demissie, D. B., et al. (2021)[79] | Critically low | Y | N |
| Deng, J., et al. (2020)[314] | Moderate | Y | N |
| Deng, J., et al. (2021)[80] | Critically low | Y | N |
| Dettmann, L. M., et al. (2022)[81] | Low | Y | N |
| Dong, F. (2021, Front. Psychol.)[83] | Low | Y | N |
| Dong, F. (2021, J. Affect. Disord)[84] | Critically low | Y | N |
| Dutta, A., et al. (2021)[90] | Low | Y | N |
| Fan, F. C., et al. (2021)[93] | Critically low | Y | N |
| Fan, S., et al. (2020)[94] | Critically low | Y | N |
| Han, Q., et al. (2022)[107] | Low | Y | N |
| Hao, Q., et al. (2021)[109] | Low | Y | N |
| Hessami, K., et al. (2020)[112] | Critically low | N | Y |
| Hosen, I., et al. (2021)[115] | Critically low | Y - Supplementary model only | N |
| Hossain, M. M., et al. (2021)[116] | Low | Y - Supplementary model only | N |
| Hu, N., et al. (2022)[117] | Critically low | Y | N |
| Johns, G., et al. (2022)[126] | Low | Y | N |
| Khraisat, B. R., et al. (2022)[134] | Critically low | Y | N |
| Kunzler, A. M., et al. (2021)[142] | Critically low | N | Y |
| Kuroda, N., et al. (2021)[143] | Critically low | Y | N |
| Lee, K. W., et al. (2022)[147] | Critically low | Y | N |
| Lee, Y., et al. (2021)[322] | Moderate | Y | N |
| Li, W., et al. (2021)[149] | Low | Y | N |
| Li, W., et al. (2022)[150] | Critically low | Y | N |
| Li, Y. et al. (2021, Front. Psychol.)[323] | Moderate | Y | N |
| Luo, W., et al. (2021)[160] | Critically low | Y | N |
| Mahmud, S., et al. (2021)[164] | Low | Y | N |
| Marvaldi, M., et al. (2021)[169] | Critically low | Y | N |
| Min, S., et al. (2021)[173] | Critically low | N | Y |
| Mulyadi, M., et al. (2021)[181] | Critically low | Y | N |
| Nagarajan, R., et al. (2022)[183] | Critically low | Y | N |
| Nochaiwong, S., et al. (2021)[325] | Moderate | Y | N |
| Norhayati, M. N., et al. (2021)[191] | Low | Y | N |
| Panda, P. K., et al. (2020)[203] | Low | Y | N |
| Pappa, S., et al. (2021)[205] | Low | Y | N |
| Premraj, L., et al. (2022)[211] | Critically low | Y | N |
| Qiu, D., et al. (2021, Eur. Psychiatry)[213] | Low | Y - Supplementary model only | N |
| Ren, X., et al. (2020)[218] | Critically low | Y - Supplementary model only | N |
| Rezaei-Hachesu, V., et al. (2022)[219] | Critically low | Y | N |
| Rezaei, S., et al. (2022)[220] | Critically low | Y | N |
| Robinson, E., et al. (2022)[222] | Critically low | N | Y |
| Rogers, J. P., et al. (2021)[226] | Low | Y | N |
| Safi-Keykaleh, M., et al. (2022)[231] | Critically low | Y | N |
| Salari, N., et al. (2020, Glob. Health)[233] | Low | Y | N |
| Salari, N., et al. (2020, Hum. Resour. Health)[234] | Critically low | Y | N |
| Salazar de Pablo, G., et al. (2020)[235] | Critically low | Y | N |
| Salehi, M., et al. (2021)[236] | Critically low | Y | N |
| Santomauro, D.F., et al. (2021)[242] | Critically low | Y | N |
| Shorey, S. Y., et al. (2021)[252] | Critically low | Y | N |
| Slusarska, B., et al. (2022)[259] | Critically low | Y | N |
| Sun, F., et al. (2020)[268] | Critically low | Y | N |
| Tomfohr-Madsen, L. M., et al. (2021)[274] | Critically low | Y | N |
| Varghese, A., et al. (2021)[280] | Low | Y | N |
| Wang, C., et al. (2021)[285] | Critically low | Y | N |
| Wu, T., et al. (2021)[293] | Critically low | Y | N |
| Xiong, N., et al. (2022)[296] | Critically low | Y | N |
| Yan, H., et al. (2020)[299] | Critically low | Y | Y |
| Yan, H., et al. (2021)[300] | Low | Y | N |
| Yan, Y., et al. (2022)[301] | Critically low | Y - Supplementary model only | N |
| Yunitri, N., et al. (2021)[304] | Low | Y | N |
| Zhang, H., et al. (2021)[329] | Moderate | Y | N |
| Zhang, S. X., et al. (2022, Eur. J. Psychotraumatol)[338] | Moderate | Y - Supplementary model only | N |
| Zhang, S. X., et al. (2022, Int. J. Environ. Health Res)[309] | Low | Y - Supplementary model only | N |
| Zhang, Y., et al. (2021)[310] | Critically low | Y | N |
| Zhao, Y. J., et al. (2021)[311] | Critically low | Y | N |
| Zhou, Y., et al. (2021)[312] | Critically low | Y | N |

## S10 Table. Countries distribution of original studies in meta-review

| **Review** | **Country approach** | **Number of COVID studies in MA** | **MA countries (COVID studies only)** | **MA continents (COVID studies only)** |
| --- | --- | --- | --- | --- |
| Abdulla, E. K. (2021)[2] | Single-country review | 23 | India (23) | Asia |
| Adibi, A. (2021)[5] | Multi-country review | 19 | South Korea (1) Saudi Arabia (1) Jordan (1) Ecuador (1) India (1) Thailand (1) United States (2) China (11) | Asia North America South America |
| Al Maqbali, M. (2021)[10] | Multi-country review | 93 | China (49) Turkey (4) Iran (4) Italy (3) Germany (2) Jordan (2) Nepal (2) Pakistan (2) Spain (2) United States (2) United Kingdom (2) Austria (1) Bahrain (1) Croatia (1) Egypt (1) Ethiopia (1) France (1) Greece (1) South Korea (1) Kosovo (1) Saudi Arabia (1) Malawi (1) Mongolia (1) Poland (1) Portugal (1) Russia (1) Singapore (1) Switzerland (1)  Multi-country (2) | Asia Africa Europe South America North America |
| Altieri, M., et al. (2022)[18] | Multi-country review | 12 | Spain (1)  Italy (5)  Multinational (1)  Turkey (1)  UK (1)  Iran (1)  Serbia (1)  Egypt (1) | Europe  Asia  Africa |
| Aymerich, C. (2022)[24] | Multi-country review | 239 | Australia (2) Bahrain (1) Belgium (1) Brazil (2) Cameroon (1) Canada (2) China (74) Colombia (1) Croatia (2) Cyprus (1) Egypt (3) Ethiopia (4) Finland (2) France (5) Germany (1) Greece (2) India (12) Indonesia (2) Iran (5) Israel (2) Italy (16) Japan (2) Jordan (2) Kosovo (1) Lebanon (1) Malaysia (3)  Mexico (2) Nepal (1) Nigeria (1) Norway (1) Oman (5) Pakistan (7) Philippines (2) Poland (2) Portugal (1) Qatar (1) Russia (1) Saudi Arabia (5) Serbia (2) Singapore (5) South Korea (3) Spain (7) Togo (1) Tunisia (1) Turkey (15) United Kingdom (5) United States (11) Vietnam (2)  Multi-country (6) | Oceania Asia Europe Africa South America North America |
| Badenoch, J. B. (2022)[27] | Multi-country review | 51 | China (13) Italy (7) United Kingdom (5) United States (5) Iran (1) Israel (1) Germany (1) Norway (2) Faroe Islands (1) Netherlands (2) Spain (3) South Korea (1) Japan (1) Austria (1) France (2) Bangladesh (2) Switzerland (1) Ireland (1)  Multi-country (1) | Asia Europe North America |
| Balakrishnan, V. (2022)[28] | Multi-country review | 82 | China (33) Japan (5) Hong Kong (2) South Korea (1) United States (13) Canada (6) Bangladesh (7) India (4) Nepal (3) Sri Lanka (1) Australia (4) Malaysia (3) | Asia North America Oceania |
| Bareeqa, S.B. (2020)[30] | Single-country review | 19 | China (19) | Asia |
| Batra, K. (2021)[33] | Multi-country review | 27 | China (14) India (1) Israel (1) Jordan (1) Saudi Arabia (1) Turkey (2) France (1) Greece (1) Italy (1) Albania (1) Brazil (1) United States (1)  Multi-country (1) | Asia Europe North America South America |
| Bussières, E. L., et al. (2021)[46] | Multi-country review | 28 | Netherlands (3)  UK (3)  Italy (7)  China (2)  South Korea (1)  Switzerland (1)  USA (1)  Israel (1)  Spain (3)  Singapore (1)  Canada (1)  Argentina (1)  Japan (1)  Turkey (1)  Germany (1) | Europe  Asia  North America  South America |
| Cenat, J.M. (2021)[55] | Multi-country review | 68 | China (45) Italy (4) Iran (5) Malaysia (1) France (1) Pakistan (1) United States (1) Bolivia (1) Ecuador (1) Vietnam (1) Spain (1) Turkey (1) Peru (1) Israel (1)  Multi-country (3) | Asia Europe North America South America |
| Chai, J. (2021)[57] | Single-country review | 12 | China (12) | Asia |
| Chang, J. J. (2021)[58] | Multi-country review | 16 | France (3) United States (2) China (5) Malaysia (1) Poland (1) India (1) Bangaladesh (1) Greece (1) Turkey (1) | Europe Asia North America |
| Chekole, Y. A. (2021)[60] | Multi-country review | 21 | China (12) India (1) Iran (1) Italy (2) Jordan (1) Singapore (1) Spain (1) United Kingdom (1) Vietnam (1) | Asia Europe |
| Chen, J. (2021)[61] | Multi-country review | 28 | Cameroon (2) Egypt (6) Ethiopia (7) Libya (3) Mali (1) Morocco (2) Nigeria (3) Republic Democratic Congo (1) Rwanda (1) South Africa (2) Togo (2) Tunisia (2) | Africa |
| Ching, S. M. (2021)[64] | Multi-country review | 148 | China (70) India (15) Turkey (11) Saudi Arabia (10) Pakistan (6) Indonesia (4) Nepal (4) Malaysia (3) Singapore (3) Japan (3) Iran (3) Oman (2) Jordan (2) Philippines (2) Bangaladesh (2) South Korea (1) Qatar (1) Iraq (1)  Multi-country (3) | Asia Africa North America |
| Chmielewska, B., et al. (2021)[318] | Multi-country review | 31 | Turkey (1)  Netherlands (1)  USA (7)  Canada (1)  UK (3)  Botswana (1)  Italy (4)  India (3)  China (4)  Denmark (1)  Hong Kong (1)  Israel (5)  Japan (2)  Nepal (1)  Mexico (1)  Ireland (3)  Brazil (1) | North America  Europe  Africa  Asia  South America |
| Crocamo, C. (2021)[69] | Multi-country review | 14 | Italy (1) India (2) United States (1) China (7) Spain (1) South Korea (1)  Multi-country (1) | Europe Asia North America South Africa |
| Demissie, D. B. (2021)[79] | Multi-country review | 19 | Canada (4) China (4) Iran (2) Belgium (1) United Kingdom (1) Turkey (1) Ireland (1) Italy (1) United States (1) Colombia (1) Sri Lanka (1)  Multi-country (1) | South America North America Asia Europe |
| Deng, J. (2020)[314] | Multi-country review | 31 | China (27) Italy (1) Ecuador (1) Turkey (1) Iran (1) | Asia Europe South America |
| Deng, J. (2021)[80] | Multi-country review | 89 | China (51) United States (5) Bangladesh (4) France (3) Spain (3) India (2) Jordan (1) Malaysia (1) Saudi Arabia (1) Italy (1) Ukraine (1) Lebanon (1) Indonesia (1) Poland (1) Turkey (1) United Arab Emirates (1) Argentina (1) Egypt (1) Pakistan (1) Ethiopia (1) Slovakia (1) Switzerland (1)  United Kingdom (1)  Multi-country (4) | Asia Africa North America Europe South America |
| Dettmann, L. M. (2022)[81] | Single-country review | 14 | United Kingdom (14) | Europe |
| Dong, F. (2021)[84] | Multi-country review | 38 | China (31) Italy (1) Iran (1) India (1) South Korea (1) Ecuador (1) Switzerland (1) Germany (1) | Asia Europe South America |
| Dong, F. (2021)[83] | Single-country review | 22 | China (22) | Asia |
| Dutta, A. (2021)[90] | Multi-country review | 32 | China (17) India (3) Pakistan (2) Turkey (1) Singapore (1) Brazil (1) Italy (1) Poland (1) Iran (1) Jordan (1) Nepal (1) United States (1) India/Singapore (1) | Asia Europe North America South America |
| Fan, F. C. (2021)[93] | Multi-country review | 130 | Argentina (1) Bangladesh (4) Cameroon (1) Canada (1) China (70) Colombia (1) Egypt (2) France (3) Germany (1) Greece (2) Hong Kong (2) India (2) Iran (1) Istanbul (1) Italy (7) Japan (2) Jordan (1) South Korea (1) Nepal (1) Pakistan (2) Philippines (1) Portugal (1) Saudi Arabia (2) Sierra Leone (1) South Korea (1) Spain (4) Sweden (1) Thailand (1) Turkey (3) United States (7)  Multi-country (2) | Asia South America Africa North America Europe |
| Fan, S. (2020)[94] | Multi-country review | 19 | Israel (1) Sri Lanka (1) China (5) Turkey (2) Italy (2) Belgium (1) Colombia (1) Japan (2) United States (3) Iran (1) | Asia Europe South America North America |
| Han, Q. (2022)[107] | Multi-country review | 18 | China (7) Italy (5) Spain (4) Germany (2) | Asia Europe |
| Hao, Q. (2021)[109] | Multi-country review | 20 | China (19) Singapore (1) | Asia |
| Hessami, K., et al. (2020)[112] | Multi-country review | 8 | Canada (3)  Italy (2)  China (1)  Turkey (1)  Greece (1) | Europe  Asia  North America |
| Hosen, I. (2021)[115] | Single-country review | 24 | Bangladesh (24) | Asia |
| Hossain, M. M. (2021)[116] | Multi-country review | 35 | India (19) Bangaladesh (7) Pakistan (5) Nepal (3) Sri Lanka (1) | Asia |
| Hu, N. (2022)[117] | Single-country review | 71 | China (71) | Asia |
| Johns, G. (2022)[126] | Multi-country review | 33 | Cyprus (1) Brazil (2) United States (5) Turkey (2) Libya (2) Malaysia (1) France (3) India (3) China (6) Colombia (1) Germany (1) Croatia (1) Turkey (1) Pakistan (1)  Multi-country (3) | North America Europe Asia Africa Oceania South America |
| Khraisat, B. R. (2022)[134] | Multi-country review | 13 | Germany (3) Australia (1) Spain (2) United Kingdom (2) Canada (1) United States (1) Italy (1) Sweden (1)  Multi-country (1) | North America Europe Oceania |
| Kunzler, A. M., et al. (2021)[142] | Multi-country review | 43 | Bangladesh (1)  China (24)  Germany (1)  Greece (1)  Iran (1)  Italy (6)  Multinational (1)  Oman (1)  Singapore (1)  Spain (2)  Turkey (3)  USA (1) | Asia  Europe  North America |
| Kuroda, N. (2021)[143] | Multi-country review | 28 | Kuwait (1) Spain (4) Saudi Arabia (1) Italy (4) China (1) Malaysia (1) United States (4) Lithuania (1) India (2) United Kingdom (2) Iran (1) Brazil (2) Turkey (1) Australia (1)  Multi-country (2) | Asia Europe North America South America Oceania Africa |
| Lee, K. W. (2022)[147] | Multi-country review | 6 | Italy (1) Kenya (1) Turkey (1) India (1) United States (1) Belgium (1) | Europe Africa North America Asia |
| Lee, Yena; Lui, Leanna M. (2021)[322] | Multi-country review | 114 | Austria (1) Canada (3) France (1) Germany (4) Greece (2) Hong Kong (1) Italy (4) Ireland (3) Israel (2) Norway (1) Poland (1) Saudi Arabia (1) South Korea (3) Spain (2) Sweden (2) Switzerland (2) United Kingdom (5) United States (116) Albania (1) Bosnia and Herzegovina (1) Brazil (1) China (40) Ecuador (1) Iran (3) Jordan (1) Mexico (1) Turkey (1) Bangladesh (1) India (2) Kenya (1) Nepal (1) Pakistan (1) Vietnam (1)  Multi-country (3) | Asia Europe North America South America Africa |
| Li, W. (2021)[149] | Single-country review | 66 | China (66) | Asia |
| Li, W. (2022)[150] | Not reported | 5 | Not reported | Not reported |
| Li, Y. (2021)[323] | Multi-country review | 22 | China (12) United States (4) Jordan (1) Spain (1) Bangladesh (1) Lebanon (1) Switzerland (1)  Multi-country (1) | Asia Europe North America |
| Luo, W. (2021)[160] | Single-country review | 84 | China (84) | Asia |
| Mahmud, S. (2021)[164] | Multi-country review | 83 | China (30) Italy (6) India (6) United States (4) Pakistan (3) Iran (3) Canada (2) Bangladesh (2) Egypt (2) Oman (2) Spain (2) Turkey (2) United Kingdom (2) Australia (1) Croatia (1) Finland (1) Germany (1) Ghana (1) Greece (1) Jordan (1) Lebanon (1) Nepal (1) Philippines (1) Poland (1) Singapore (1) South Korea (2) Vietnam (1)  Multi-country (2) | Asia Europe North America Oceania Africa |
| Marvaldi, M. (2021)[169] | Multi-country review | 70 | China (30) India (6) Turkey (5) United States (4) Iraq (1) Saudi Arabia (3) Thailand (1) Egypt (2) France (1) Oman (1) Turkey (5) Ireland (1) Italy (1) Spain (2) Pakistan (2) Bahrain (1) Nepal (1) Phillippine (1) Singapore (1) Iran (2) Portugal (1) United Kingdom (1) Germany (1) Serbia (1)  Multi-country (2) | Asia Europe North America Africa |
| Min, S., et al. (2021)[173] | Multi-country review | 15 | USA (5)  Italy (2)  Czech (2)  Germany (1)  Spain (1)  UK (3)  Switzerland (1) | North America  Europe |
| Mulyadi, M. (2021)[181] | Multi-country review | 17 | Turkey (5) China (3) Indonesia (3) United States (2) Israel (1) Italy (1) Nepal (1)  Multi-country (1) | Asia Europe Oceania North America |
| Nagarajan, R. (2022)[183] | Multi-country review | 13 | Italy (3) Netherlands (1) Switzerland (1) United Kingdom (2) France (1) Turkey (1) China (1) Iran (1) United States (1) Austria (1) | Europe Asia North America |
| Nochaiwong, S. (2021)[325] | Multi-country review | 107 | Nigeria (1) South Africa (1) Brazil (4) Mexico (1) United States (7) Bangladesh (3) India (5) Nepal (1) Thailand (1) Germany (1) Greece (1) Ireland (1) Italy (6) Norway (1) Portugal (1) Spain (6) Sweden (1) Turkey (2) United Kingdom (7) Egypt (1) Iran (3) Jordan (2) Pakistan (1) Saudi Arabia (2) Tunisia (1) United Arab Emirates (2) Australia (4) China (34) Hong Kong (2) Japan (1) Malaysia (1) New Zealand (1) Vietnam (1) | Africa South America Asia Europe North America Oceania |
| Norhayati, M. N. (2021)[191] | Multi-country review | 80 | Saudi Arabia (6) Yemen (1) Kuwait (1) Oman (1) Pakistan (3) China (36) Japan (1) Bangladesh (2) India (5) Iran (3) Nepal (3) Turkey (5) Malaysia (2) Jordan (1) South Korea (3) Sri Lanka (1) Singapore (1) Vietnam (1) Indonesia (1)  Multi-country (3) | Asia |
| Panda, P.K. (2020)[203] | Multi-country review | 15 | China (5) France (1) Italy (2) India (1) Hong Kong (1) Brazil (1) Turkey (1) Bangladesh (1) South Korea (1)  Multi-country (1) | Asia Europe South America |
| Pappa, S. (2021)[205] | Multi-country review | 32 | Indonesia (4) Malaysia (12) Philippines (1) Singapore (9) Thailand (2) Vietnam (4) | Asia |
| Premraj, L. (2022)[211] | Multi-country review | 18 | Germany (1) United States (1) Spain (2) China (3) Switzerland (1) Italy (1) Iran (1) United Kingdom (1)  Multi-country (2)  Not reported (5) | Europe North America South America Africa Oceania Asia |
| Qiu, D. (2021)[213] | Multi-country review | 76 | Australia (1) Austria (1) Brazil (1) China (40) Egypt (1) France (2) Germany (1) Greece (1) India (1) Ireland (1) Israel (1) Italy (7) South Korea (1) Mexico (2) Philippines (1) Saudi Arabia (1) Singapore (1) Spain (3) Tunisia (1) Turkey (1) United States (4) Vietnam (1) Not reported (2) | Africa Australia Asia Europe North America South America |
| Ren, X. (2020)[218] | Single-country review | 12 | China (12) | Asia |
| Rezaei, S. (2022)[220] | Multi-country review | 24 | Not reported (Majority China) | Not reported |
| Rezaei-Hachesu, V. (2022)[219] | Single-country review | 10 | Iran (10) | Asia |
| Robinson, E., et al. (2022)[222] | Multi-country review | 65 | Australia (2)  Brazil (1)  China (1)  Hong Kong (2)  Czech (1)  Denmark (1)  Finland (1)  Germany (4)  India (2)  Italy (6)  Japan (2)  Netherlands (4)  New Zealand (1)  Norway (1)  Scotland (1)  Slovakia (1)  Spain (1)  Sweden (1)  Switzerland (2)  Turkey (2)  UK (8)  USA (16)  Multi-country (3) | Oceania  South America  Asia  Europe  North America |
| Rogers, J. P. (2021)[226] | Multi-country review | 147 | Not reported | Not reported |
| Safi-Keykaleh, M. (2022)[231] | Multi-country review | 24 | China (2) Serbia (1) United Kingdom (2) Turkey (2) Japan (3) Poland (1) Brazil (1) Spain (1) Italy (4) Israel (1) Mexico (1) Belgium (1) Hong Kong (2) Argentina (1) Saudi Arabia (1)  Multi-country (1) | Asia Europe South America |
| Salari, N. (2020)[233] | Multi-country review | 17 | China (8) Iran (1) Japan (1)  Nepal (1) India (1) Iraq (1) United Kingdom (1) Spain (1) Nigeria (1) Italy (1) | Asia Europe Africa |
| Salari, N. (2020)[234] | Multi-country review | 29 | China (19) Singapore (2) Hong Kong (2) Iran (2) Romania (1) India (1) France (1) Australia (1) | Asia Europe Oceania |
| Salazar de Pablo, G. (2020)[235] | Not reported | Not reported | Not reported | Not reported |
| Salehi, M. (2021)[236] | Multi-country review | 12 | Singapore (1) Spain (1) China (9)  Multi-country (1) | Asia Europe |
| Santomauro, D. F. (2021)[242] | Multi-country review | 48 | Spain (2) Australia (2) New Zealand (3) United Kingdom (6) England (2) United States (13) Japan (5) Germany (2) Netherlands (1) China (1) Hong Kong (1) Ireland (1) Norway (1) France (2) Austria (2) Denmark (2) Czech Republic (1)  Multi-country (1) | Europe Oceania Asia North America |
| Shorey, S. Y. (2021)[252] | Multi-country review | 26 | Canada (3) China (5) Belgium (1) Greece (1) Turkey (3) Iran (1) United States (3) Hong Kong (1) Italy (3) Japan (2) Israel (2) Sri Lanka (1) | Asia Europe North America |
| Slusarska, B. (2022)[259] | Multi-country review | 23 | Saudi Arabia (1) China (13) Philippines (2) Iran (1) Brazil (1) United States (2) Canada (1) United Kingdom (1) Turkey (1) | Asia North America South America |
| Sun, F. (2020)[268] | Multi-country review | 15 | Canada (1) Israel (2) Turkey (1) China (3) Italy (2) United States (1) Japan (1) Belgium (1) Sri Lanka (1) Ireland (1) Not reported (1) | North America Asia Europe |
| Tomfohr-Madsen, L. M. (2021)[274] | Multi-country review | 46 | Iran (2) Singapore (1) China (14) Spain (1) Canada (3) Belgium (1) Greece (1) Turkey (3) Qatar (1) United States (4) United Kingdom (1) Japan (3) Argentina (1) Mexico (1) Italy (4) Pakistan (1) Sri Lanka (1) Poland (1)  Multi-country (2) | North America Europe Asia South America |
| Varghese, A. (2021)[280] | Multi-country review | 27 | Brazil (1) Croatia (1) Germany (2) Poland (1) Russia (1) Italy (1) Jordan (2) China (9) Vietnam (2) Turkey (2) Singapore (1) Philippines (1) Oman (1) Iran (1) India (1)  Multi-country (1) | Asia Europe South America |
| Wang, C. (2021)[285] | Multi-country review | 28 | China (15) United States (3) Vietnam (1) Malaysia (1) Bangladesh (2) Israel (1) Spain (1) Jordan (1) Colombia (1)  Multi-country (3) | Asia North America Europe South America |
| Wu, T. (2021)[293] | Multi-country review | 66 | China (62) Iran (1) Jordan (1) Singapore (1) India (1) | Asia |
| Xiong, N. (2022)[296] | Single-country review | 44 | China (44) | Asia |
| Yan, H. (2020)[299] | Multi-country review | 23 | China (7) Sri Lanka (1) Israel (1) Japan (1) Turkey (3) Colombia (1) United States (3) Canada (2) Italy (3) Belgium (1) | Asia Europe South America North America |
| Yan, H. (2021)[300] | Single-country review | 35 | China (35) | Asia |
| Yan, Y. (2022)[301] | Single-country review | 17 | China (17) | Asia |
| Yunitri, N., et al. (2021)[304] | Multi-country review | 63 | Brazil (1)  Canada (1)  China (30)  France (2)  Greece (1)  Ireland (1)  Israel (1)  Italy (10)  Mexico (1)  Multinational- Asia (2)  Norway (2)  Poland (1)  Saudi Arabia (2)  South Korea (1)  Spain (2)  Tunisia (1)  Turkey (1)  UK (1)  USA (2) | South America  North America  Asia  Europe  Africa |
| Zhang, H. (2021)[329] | Single-country review | 26 | China (26) | Asia |
| Zhang, S. X. (2022)[309] | Single-country review | 28 | Spain (28) | Europe |
| Zhang, S. X. (2022)[338] | Multi-country review | 21 | Albania (2) Bosnia and Herzegovina (1) Bulgaria (1) Croatia (3) Czech Republic (1) Kosovo (1) Poland (4) Russia (3) Serbia (4) Ukraine (1) | Europe |
| Zhang, Y. (2021)[310] | Single-country review | 31 | China (31) | Asia |
| Zhao, Y. J. (2021)[311] | Multi-country review | 36 | China (31) Italy (2) Israel (1) Spain (1)  Multi-country (1) | Asia Europe |
| Zhou, Y. (2021)[312] | Single-country review | 6 | China (6) | Asia |

## S11 Table. Meta-analyses excluded from meta-review

| **K = 75** |  |
| --- | --- |
| **First author et al (year)** | **Reason for exclusion from meta-review** |
| Acuff, S. F., et al. (2022)[4] | Did not provide estimates of prevalence or comparison with pre-pandemic |
| Al Falasi, B., et al. (2021)[8] | Combines COVID and non-COVID data |
| Alimoradi, Z., et al. (2021)[12] | Did not provide estimates of prevalence or comparison with pre-pandemic |
| Alimoradi, Z., et al. (2022)[330] | Did not provide estimates of prevalence or comparison with pre-pandemic |
| Allan, S. M., et al. (2020)[14] | Combines COVID and non-COVID data |
| Alzahrani, F., et al. (2022)[19] | No sample N provided |
| Arora, T., et al. (2020)[23] | No sample N provided |
| Ayubi, E., et al. (2021)[26] | No sample N provided |
| Blasco-Belled, A., et al. (2022)[39] | No sample N provided |
| Boden, M., et al. (2021)[40] | Reported infrequently explored outcome (insufficient reviews to pool results) |
| Bueno-Notivol, J., et al. (2021)[44] | No sample N provided |
| Busch, I. M., et al. (2021)[45] | Combines COVID and non-COVID data |
| Caruso, R., et al. (2021)[53] | Did not provide estimates of prevalence or comparison with pre-pandemic |
| Cheng, C. K. T., et al. (2022)[62] | Did not provide estimates of prevalence or comparison with pre-pandemic |
| da Silva, F. C. T., et al. (2021, Prog. Neuropsychopharmacol. Biol., vol 104)[74] | Did not provide estimates of prevalence or comparison with pre-pandemic |
| da Silva, M. L., et al. (2020)[75] | Combines COVID and non-COVID data |
| Dorri, M., et al. (2021)[85] | No sample N provided |
| Dragioti, E., et al. (2021)[87] | No sample N provided |
| Dube, J. P., et al. (2021)[88] | Reported infrequently explored outcome (insufficient reviews to pool results) |
| Farooq, S., et al. (2021)[95] | Reported infrequently explored outcome (insufficient reviews to pool results) |
| Ghazanfarpour, M., et al. (2021)[99] | No sample N provided |
| Guo, S., et al. (2021)[105] | Reported infrequently explored outcome (insufficient reviews to pool results) |
| Hill, J. E., et al. (2022)[113] | No sample N provided |
| Khraisat, B., et al. (2021)[135] | No sample N provided |
| Kilian, C., et al. (2022)[337] | Did not provide estimates of prevalence or comparison with pre-pandemic |
| Kisely, S., et al. (2020)[138] | Combines COVID and non-COVID data |
| Krishnamoorthy, Y., et al. (2020)[140] | No sample N provided |
| Lasheras, I., et al. (2020)[145] | No sample N provided |
| Li, Y., et al. (2021, PLoS ONE)[151] | No sample N provided |
| Lin, C. Y., et al. (2020)[154] | Did not provide estimates of prevalence or comparison with pre-pandemic |
| Liu, C. (2021 J. Psychosom. Res.)[336] | No sample N provided |
| Liyanage, S., et al. (2021)[156] | No sample N provided |
| Luo, F., et al. (2021)[157] | Did not provide estimates of prevalence or comparison with pre-pandemic |
| Luo, M., et al. (2020)[158] | No sample N provided |
| Luo, Y., et al. (2022)[162] | Did not provide estimates of prevalence or comparison with pre-pandemic |
| Ma, L., et al. (2021)[163] | No sample N provided |
| Mahmud, S., et al. (2022)[165] | No sample N provided |
| Malik, P., et al. (2022)[166] | Did not provide estimates of prevalence or comparison with pre-pandemic |
| Marciano, L., et al. (2022)[167] | Did not provide estimates of prevalence or comparison with pre-pandemic |
| Nam, S. H., et al. (2021)[185] | Did not provide estimates of prevalence or comparison with pre-pandemic |
| Necho, M., et al. (2021)[188] | No sample N provided |
| Neelam, K., et al. (2020)[189] | Combines COVID and non-COVID data |
| Nowrouzi-Kia, B., et al. (2021)[192] | Combines COVID and non-COVID data |
| Olaya, B., et al. (2021)[196] | No sample N provided |
| Oliveira Carvalho, P., et al. (2021)[197] | No sample N provided |
| Ozamiz-Etxebarria, N., et al. (2021)[199] | No sample N provided |
| Ozguc, S., et al. (2021)[200] | Did not provide estimates of prevalence or comparison with pre-pandemic |
| Pappa, S., et al. (2020)[204] | No sample N provided |
| Pashazadeh Kan, F., et al. (2021)[206] | No sample N provided |
| Phiri, P., et al. (2021)[207] | No sample N provided |
| Qiu, D., et al. (2021, Front. Psychol.)[214] | No sample N provided |
| Racine, N., et al. (2021)[215] | No sample N provided |
| Racine, N., et al. (2022)[332] | No sample N provided |
| Raoofi, S., et al. (2021)[216] | No sample N provided |
| Rogers, J. P., et al. (2020)[225] | Combines COVID and non-COVID data |
| Runacres, A., et al. (2021)[228] | Did not provide estimates of prevalence or comparison with pre-pandemic |
| Santabarbara, J., et al. (2020, July. Prog. Neuropsychopharmacol. Biol. Psychiatry)[239] | No sample N provided |
| Santabarbara, J., et al. (2021, Medicina [Kaunas])[240] | No sample N provided |
| Santabarbara, J., et al. (2021, Prog. Neuropsychopharmacol. Biol. Psychiatry)[241] | No sample N provided |
| Saragih, I. D., et al. (2021)[243] | No sample N provided |
| Sheraton, M., et al. (2020)[251] | Did not provide estimates of prevalence or comparison with pre-pandemic |
| Schubert, M., et al. (2021)[246] | Did not provide estimates of prevalence or comparison with pre-pandemic |
| Serrano-Ripoll, M. J., et al. (2020)[248] | Combines COVID and non-COVID data |
| Sideli, L., et al. (2021)[254] | No sample N provided |
| Simsir, Z., et al. (2021)[257] | Did not provide estimates of prevalence or comparison with pre-pandemic |
| Singh, R. K., et al. (2021)[258] | No sample N provided |
| Soltani, S., et al. (2021)[263] | No sample N provided |
| Soysal, P., et al. (2022)[264] | Reported infrequently explored outcome (insufficient reviews to pool results) |
| Sun, P., et al. (2021)[269] | No sample N provided |
| Thakur, B., et al. (2021)[271] | No sample N provided |
| Wang, F., et al. (2022)[286] | Did not provide estimates of prevalence or comparison with pre-pandemic |
| Wang, Y., et al. (2020)[287] | No sample N provided |
| Yuan, Kai, et al. (2021)[303] | No sample N provided |
| Zhang, L., et al. (2021)[307] | No sample N provided |
| Zhu, J., et al. (2021)[313] | No sample N provided |

## S12 Table. Heterogeneity and risk of bias information for pooled estimates included in meta-review of pooled prevalence

| **Review** | **I^2^** | **p value** | **Heterogeneity level** | **Other heterogeneity statistics** | **Risk of Bias / Quality Appraisal** | **Publication bias** |
| --- | --- | --- | --- | --- | --- | --- |
| Depression – COVID-19 patients | | | | | | |
| Premraj, L. (2022)[211] | 97.63 | <0.001 | Considerable heterogeneity |  | Low risk of bias (NOS). | NR |
| Rogers, J. P. (2021)[226] | NR | NR | NA |  | NR - Authors used NOS but did not report overall risk of bias. | NR |
| Dong, F. (2021, J. Affect. Disord)[84] | 98.3 | <0.001 | Considerable heterogeneity |  | NR | Egger's test (p = 0.015) indicated plot asymmetry. Begg's test (p = 0.210) indicated no publication bias. |
| Wu, T. (2021)[293] | 90.3 | NR | Considerable heterogeneity |  | Low to moderate risk of bias (STROBE). | Egger's test and funnel plot inspection indicated no publication bias. |
| Deng, J. (2020)[314] | 96 | <0.01 | Considerable heterogeneity |  | Low to high risk of bias (NOS). | Egger's test (p = 0.22) and funnel plot inspection indicated no publication bias. |
| Depression – General population | | | | | | |
| Pappa, S. (2021)[205] | 98.88 | <0.001 | Considerable heterogeneity |  | NR - Authors used MMAT but did not report overall risk of bias. | LFK index indicated no publication bias. |
| Han, Q. (2022)[107] | 89.6 | <0.001 | Considerable heterogeneity |  | Low to moderate risk of bias (JBI Checklist). | Egger's test indicated no publication bias. |
| Dettmann, L. M. (2022)[81] | 97.7 | <0.001 | Considerable heterogeneity |  | Low risk of bias (NOS). | Egger's test (p = 0.05) indicated publication bias. |
| Balakrishnan, V. (2022)[28] | 99.742 | NR | Considerable heterogeneity |  | Low risk of bias (STROBE). | Funnel plot inspection and Egger's test (p = 0.249) indicated low publication bias. |
| Badenoch, J. B. (2022)[27] | 98.6 | NR | Considerable heterogeneity |  | NR | NR |
| Nochaiwong, S. (2021)[325] | 99.7 | <0.001 | Considerable heterogeneity | Chi2 = 24.55 | NR- Authors used Hoy's Risk of Bias Tool but did not report overall risk of bias. | Egger's test indicated no publication bias. |
| Li, W. (2021)[149] | 99.99 | <0.001 | Considerable heterogeneity |  | NR - Authors used STROBE but did not report overall risk of bias. | NR |
| Lee, Yena; Lui, Leanna M. (2021)[322] | 100 | <0.001 | Considerable heterogeneity | Tau2 = 0.0195 Chi2 = 185.88 | Moderate to serious risk of bias (ROBINS-I). | NR |
| Santomauro, D. F. (2021)[242] | NR | NR | NR |  | NR | NR |
| Fan, F. C. (2021)[93] | 99.611 | <0.001 | Considerable heterogeneity | Q = 27525.37 | NR | NR |
| Zhao, Y. J. (2021)[311] | 99.38 | <0.001 | Considerable heterogeneity |  | Majority moderate quality (Loney's 8-item scale). | Egger’s test indicated marginal publication bias. |
| Wu, T. (2021)[293] | 99.8 | <0.01 | Considerable heterogeneity |  | Low to moderate risk of bias (STROBE). | Egger's test and funnel plot inspection indicated no publication bias. |
| Panda, P.K. (2020)[203] | 34 | 0.037 | Low heterogeneity |  | Low to moderate risk of bias (ROBINS-I). | Egger's test indicated no publication bias. |
| Bareeqa, S.B. (2020)[30] | 99.68 | <0.001 | Considerable heterogeneity |  | Low to high risk of bias (NOS). | NR |
| Salari, N. (2020, Glob. Health)[233] | 99.4 | <0.001 | Considerable heterogeneity |  | Medium to high quality (STROBE). | Egger's test (p = 0.073) indicated no publication bias. |
| Depression – HCW | | | | | | |
| Xiong, N. (2022)[296] | 94.34 | <0.001 | Considerable heterogeneity |  | NR | Egger's test (p < 0.001) indicated publication bias. |
| Slusarska, B. (2022)[259] | 99.71 | NR | Considerable heterogeneity |  | NR - Authors used AHRQ but did not report overall risk of bias. | Egger's test indicated (p = 0.32) no publication bias. |
| Rezaei-Hachesu, V. (2022)[219] | 99.01 | <0.001 | Considerable heterogeneity |  | NR | NR |
| Rezaei, S. (2022)[220] | NR | NR | NA |  | NR - Authors used NOS but did not report overall risk of bias. | Egger's test (p = 0.67) indicated no publication bias. |
| Johns, G. (2022)[126] | 98.931 | <0.001 | Considerable heterogeneity |  | Low to medium risk of bias (JBI Checklist). | Egger's test (p = 0.676) indicated no publication bias. |
| Hu, N. (2022)[117] | 100 | <0.001 | Considerable heterogeneity | Tau2 = 0.0633 | NR | Egger's test indicated no publication bias. |
| Aymerich, C. (2022)[24] | 99.95 | <0.001 | Considerable heterogeneity |  | NR - Authors used NOS but did not report overall risk of bias. | Egger's test - Results NR. |
| Zhang, H. (2021)[329] | 98.6 | NR | Considerable heterogeneity |  | "Poor to good quality" (NIH). | Egger's test (p = 0.52) indicated no publication bias. |
| Norhayati, M. N. (2021)[191] | 100 | <0.001 | Considerable heterogeneity |  | NR - Authors used JBI Checklist but did not report overall risk of bias. | Funnel plots - Results NR. |
| Mulyadi, M. (2021)[181] | 98 | <0.001 | Considerable heterogeneity | Q = 303.2 | Low risk of bias (JBI Checklist). | Egger's test (p = 0.16) indicated no publication bias. |
| Mahmud, S. (2021)[164] | 99.84 | <0.001 | Considerable heterogeneity | Tau2 = 502.14 Q = 34575.82 H2 = 609.74 | NR | Egger's test (Z = 2.77, p = 0.005) indicated publication bias. |
| Hao, Q. (2021)[109] | 99 | <0.01 | Considerable heterogeneity | Tau2 = 0.0066 Chi2 = 31.07 | NR | Egger's test (p = 0.3001) indicated plot asymmetry. |
| Dutta, A. (2021)[90] | 99 | <0.001 | Considerable heterogeneity | Q = 4791.18 | Low risk of bias (NOS). | NR |
| Dong, F. (2021, Front. Psychol.)[83] | 99.2 | <0.001 | Considerable heterogeneity |  | NR | Egger's test indicated plot asymmetry (p = 0.000). Begg's test reported (p = 0.198). |
| Crocamo, C. (2021)[69] | NR | NR | Considerable heterogeneity |  | NR | Egger's test - Results NR. |
| Ching, S. M. (2021)[64] | 99.49 | <0.001 | Considerable heterogeneity |  | NR - Authors used STROBE but did not report overall risk of bias. | Egger's test indicated plot asymmetry. |
| Abdulla, E. K. (2021)[2] | 99 | <0.001 | Considerable heterogeneity | Tau2 = 495.66 Chi2 = 1190.98 | NR - Authors used Downs and Black Checklist but did not report overall risk of bias. | Funnel plot inspection indicated plot asymmetry. |
| Varghese, A. (2021)[280] | 99.4 | <0.01 | Considerable heterogeneity | Q = 2659.04 | Moderate to high quality (Loney's 8-item Scale). | Egger's test (p = 0.61) indicated no publication bias. |
| Yan, H. (2021)[300] | 99 | <0.001 | Considerable heterogeneity |  | NR - Authors used STROBE but did not report overall risk of bias. | Funnel plot inspection and Begg’s rank test (p = 0.080) indicated no publication bias. |
| Luo, W. (2021)[160] | 99.5 | <0.001 | Considerable heterogeneity |  | Low to high risk of bias (JBI Checklist). | Funnel plot inspection and Egger's test (p = 0.169) indicated no publication bias. |
| Marvaldi, M. (2021)[169] | 99.55 | <0.001 | Considerable heterogeneity |  | NR - Authors used AHRQ, NIH and Crombie’s Items but did not report overall risk of bias. | Egger's test (p > 0.1) and funnel plot inspection indicated no publication bias. |
| Zhao, Y. J. (2021)[311] | 99.32 | <0.001 | Considerable heterogeneity |  | Majority moderate quality (Loney's 8-item scale). | Egger’s test indicated marginal publication bias. |
| Al Maqbali, M. (2021)[10] | 99 | <0.000 | Considerable heterogeneity |  | Low to moderate risk of bias (NOS). | Funnel plot inspection indicated publication bias. Egger’s test (p = 0.35) indicated no publication bias. |
| Wu, T. (2021)[293] | 99.5 | NR | Considerable heterogeneity |  | Low to moderate risk of bias (STROBE). | Egger's test and funnel plot inspection indicated no publication bias. |
| Salari, N. (2020, Hum. Resour. Health)[234] | 98.9 | NR | Considerable heterogeneity |  | Medium to high quality (STROBE). | Egger's test (p = 0.349) and funnel plot inspection indicated no publication bias. |
| Bareeqa, S.B. (2020)[30] | 99.3 | <0.001 | Considerable heterogeneity |  | Low to high risk of bias (NOS). | NR |
| Salazar de Pablo, G. (2020)[235] | 99.621 | <0.001 | Considerable heterogeneity | Q = 791.154 | NR - Authors used MMAT but don't report overall risk of bias. | NR |
| Depression – Pregnant and postpartum people | | | | | | |
| Safi-Keykaleh, M. (2022)[231] | 98.5 | <0.001 | Considerable heterogeneity |  | NR | Begg's test (p = 0.084) indicated no publication bias. |
| Demissie, D. B. (2021)[79] | 99.29 | 0.001 | Considerable heterogeneity |  | NR | Egger's test (p = 0.208) indicated no publication bias. |
| Shorey, S. Y. (2021)[252] | 97 | <0.001 | Considerable heterogeneity | Tau2 = 0.02  Chi2 = 98.96, df = 3, p < 0.00001, I2 = 97%. Test for overall effect Z = 4.03, p < 0.0001 | NR - Authors used JBI Checklist but did not report overall risk of bias. | Forest plot inspection indicated asymmetry. |
| Shorey, S. Y. (2021)[252] | 96 | <0.001 | Considerable heterogeneity | Tau2 = 0.01  Chi2 = 113.57, df = 4, p < 0.00001, I2 = 96%. Test for overall effect Z = 4.58, p < 0.00001 | NR - Authors used JBI Checklist but did not report overall risk of bias. | Forest plot inspection indicated asymmetry. |
| Shorey, S. Y. (2021)[252] | 99 | <0.001 | Considerable heterogeneity | Tau2 = 0.01  Chi2 = 1068.84, df = 12, p < 0.00001, I2 = 99%. Test for overall effect Z = 8.44, p < 0.00001 | NR - Authors used JBI Checklist but did not report overall risk of bias. | Forest plot inspection indicated asymmetry. |
| Tomfohr-Madsen, L. M. (2021)[274] | 98.62 | <0.001 | Considerable heterogeneity | Q = 2616.09 | NR - Used NIH but did not report overall risk of bias. | Funnel plot inspection indicated no publication bias. Egger's test (p < .06) indicated publication bias. |
| Yan, H. (2020)[299] | 99.4 | <0.001 | Considerable heterogeneity |  | NR - Authors used NOS but did not report overall risk of bias. | NR |
| Fan, S. (2020)[94] | 97.9 | <0.001 | Considerable heterogeneity |  | Majority high quality (JBI PACES). | Egger and Begg's tests indicated no publication bias. |
| Sun, F. (2020)[268] | 96 | <0.01 | Considerable heterogeneity | Tau2 = 0.0158 | High quality (AHRQ). | Forest plot inspection indicated asymmetry. |
| Depression – Vulnerable populations | | | | | | |
| Lee, K. W. (2022)[147] | 97.7 | <0.001 | Considerable heterogeneity |  | NR - Authors used STROBE but did not report overall risk of bias. | NR |
| Khraisat, B. R. (2022)[134] | 99 | <0.001 | Considerable heterogeneity | Q = 289.26 | NR | Funnel plot inspection indicated asymmetry. |
| Kuroda, N. (2021)[143] | 97 | <0.01 | Considerable heterogeneity | Tau2 = 0.0307 Chi2 = 657.19 | NR - Authors used CLARITY and JBI Checklist but did not report overall risk of bias. | Egger's test (p = 0.87) revealed significant publication bias. |
| Depression – Young people | | | | | | |
| Li, W. (2022)[150] | 99.6 | <0.001 | Considerable heterogeneity |  | NR | Egger's test (p = 0.007) and Begg's test (p = 0.014) indicated asymmetry. |
| Zhang, Y. (2021)[310] | 100 | <0.001 | Considerable heterogeneity | Tau2 = 0.281 | NR | Egger's test (p < 0.05) indicated publication bias. |
| Wang, C. (2021)[285] | 99.9 | <0.001 | Considerable heterogeneity |  | NR | Egger's test (p = 0.000) indicated publication bias. |
| Li, Y. (2021, Front. Psychol.)[323] | 99.9 | <0.001 | Considerable heterogeneity |  | NR - Authors used JBI but did not report overall risk of bias. | Egger's test (0.47) and Begg's test (p = 0.705) indicated no publication bias. |
| Deng, J. (2021)[80] | 100 | <0.01 | Considerable heterogeneity |  | Low risk of bias (NOS). | Egger's test (p = 0.42) indicated no publication bias. |
| Chang, J. J. (2021)[58] | 99.9 | <0.001 | Considerable heterogeneity |  | Low risk of bias (NOS). | Egger's test (p = 0.324) indicated no publication bias. |
| Chai, J. (2021)[57] | 99 | <0.01 | Considerable heterogeneity | Tau2 = 0.0165 | NR | Egger's test (p = 0.26) indicated no publication bias. |
| Batra, K. (2021)[33] | 99.8 | <0.001 | Considerable heterogeneity |  | Fair to good quality (NIH). | Funnel plot inspection and Egger's test (p = 0.17) indicated no publication bias. |
| Luo, W. (2021)[160] | 99.7 | <0.001 | Considerable heterogeneity |  | Low to high risk of bias (JBI). | Funnel plot inspection and Egger's test (p = 0.169) indicated no publication bias. |
| Wu, T. (2021)[293] | 99.8 | NR | Considerable heterogeneity |  | Low to moderate risk of bias (STROBE). | Egger's test and funnel plot inspection indicated no publication bias. |
| Panda, P.K. (2020)[203] | 36 | 0.02 | Low heterogeneity |  | Low to moderate risk of bias (ROBINS-I). | Egger's test indicated no publication bias. |

| **Review** | **I2** | **p value** | **Heterogeneity level** | **Other heterogeneity statistics** | **Risk of Bias / Quality Appraisal** | **Publication bias** |
| --- | --- | --- | --- | --- | --- | --- |
| Anxiety – COVID-19 patients | | | | | | |
| Premraj, L. (2022)[211] | 98.01 | <0.001 | Considerable heterogeneity |  | Low risk of bias (NOS). | NR |
| Rogers, J. P. (2021)[226] | NR | NR | NA |  | NR - Authors used NOS but did not report overall risk of bias. | NR |
| Dong, F. (2021, J. Affect. Disord)[84] | 98.2 | <0.001 | Considerable heterogeneity |  | NR | Egger's test (p = 0.000) indicated publication bias. Begg's test (p = 0.532) indicated no publication bias. |
| Wu, T. (2021)[293] | 91.5 | NR | Considerable heterogeneity |  | Low to moderate risk of bias (STROBE). | Egger's test and funnel plot inspection indicated no publication bias. |
| Deng, J. (2020)[314] | 97 | <0.01 | Considerable heterogeneity |  | Low to high risk of bias (NOS). | Egger's test (p = 0.06) and funnel plot inspection indicated no publication bias. |
| Anxiety – General population | | | | | | |
| Pappa, S. (2021)[205] | 99.3 | <0.001 | Considerable heterogeneity |  | NR - Authors used MMAT but did not report overall risk of bias. | LFK index indicated no publication bias. |
| Han, Q. (2022)[107] | 95.4 | <0.001 | Considerable heterogeneity |  | Low to moderate risk of bias (JBI Checklist). | Egger's test indicated no publication bias. |
| Dettmann, L. M. (2022)[81] | 98.71 | <0.001 | Considerable heterogeneity |  | Low risk of bias (NOS). | Egger's test (p = 0.05) indicated publication bias. |
| Badenoch, J. B. (2022)[27] | 95.8 | NR | Considerable heterogeneity |  | NR | NR |
| Nochaiwong, S. (2021)[325] | 99.7 | <0.001 | Considerable heterogeneity | Chi2 = 103.66 | NR- Authors used Hoy's Risk of Bias tool but did not report overall risk of bias. | Egger's test indicated no publication bias. |
| Li, W. (2021)[149] | 99.99 | <0.001 | Considerable heterogeneity |  | NR - Authors used STROBE but did not report overall risk of bias. | NR |
| Santomauro, D. F. (2021)[242] | NR | NR | NR |  | NR | NR |
| Fan, F. C. (2021)[93] | 99.824 | 0 | Considerable heterogeneity | Q = 61270.18 | NR | NR |
| Zhao, Y. J. (2021)[311] | 98.74 | <0.001 | Considerable heterogeneity |  | Majority moderate quality (Loney's 8-item scale). | Egger’s test indicated marginal publication bias. |
| Wu, T. (2021)[293] | 99.8 | <0.01 | Considerable heterogeneity |  | Low to moderate risk of bias (STROBE). | Egger's test and funnel plot inspection indicated no publication bias. |
| Panda, P.K. (2020)[203] | 61 | 0.001 | Substantial heterogeneity |  | Low to moderate risk of bias (ROBINS-I). | Egger's test indicated no publication bias. |
| Bareeqa, S.B. (2020)[30] | 99.52 | <0.001 | Considerable heterogeneity |  | Low to high risk of bias (NOS). | NR |
| Salari, N. (2020, Glob. Health)[233] | 99.3 | <0.001 | Considerable heterogeneity |  | Medium to high quality (STROBE). | Egger's test (p = 0.064) indicated no publication bias. |
| Anxiety – HCW | | | | | | |
| Xiong, N. (2022)[296] | 99.49 | <0.001 | Considerable heterogeneity |  | NR | Egger's test (p = 0.003) indicated publication bias. |
| Slusarska, B. (2022)[259] | 99.92 | NR | Considerable heterogeneity |  | NR - Authors used AHRQ but did not report overall risk of bias. | Egger's test (p = 0.32) indicated no publication bias. |
| Rezaei-Hachesu, V. (2022)[219] | 98.93 | <0.001 | Considerable heterogeneity |  | NR | NR |
| Johns, G. (2022)[126] | 99.19 | <0.001 | Considerable heterogeneity |  | Low to medium risk of bias (JBI). | Egger's test (p = 0.8973) indicated no publication bias. |
| Hu, N. (2022)[117] | 100 | <0.001 | Considerable heterogeneity | Tau2 = 0.1182 | NR | Egger's test indicated no publication bias. |
| Aymerich, C. (2022)[24] | 99.94 | <0.001 | Considerable heterogeneity |  | NR - Authors used NOS but did not report overall risk of bias. | Egger's test - Results NR. |
| Zhang, H. (2021)[329] | 99 | NR | Considerable heterogeneity |  | "Poor to good quality" (NIH). | Egger's test (p = 0.58) indicated no publication bias. |
| Norhayati, M. N. (2021)[191] | 100 | <0.001 | Considerable heterogeneity |  | NR - Authors used JBI but did not report overall risk of bias. | Funnel plots - Results NR. |
| Mulyadi, M. (2021)[181] | 98 | <0.001 | Considerable heterogeneity | Q = 409.68 | Low risk of bias (JBI Checklist). | Egger's test (p = 0.79) indicated no publication bias. |
| Mahmud, S. (2021)[164] | 99.79 | <0.001 | Considerable heterogeneity | Tau2 = 534.31 Q = 47974.95 H2 = 481.82 | NR | Egger's test (Z = 2.22, p = 0.02) indicated publication bias. |
| Hao, Q. (2021)[109] | 99 | <0.01 | Considerable heterogeneity | Tau2 = 0.23333 | NR | Egger's test (p = 0.1045) indicated plot asymmetry. |
| Dutta, A. (2021)[90] | 99 | <0.001 | Considerable heterogeneity | Q = 3102.38 | Low risk of bias (NOS). | NR |
| Dong, F. (2021, Front. Psychol.)[83] | 98.8 | <0.001 | Considerable heterogeneity |  | NR | Egger's test (p = 0.000) indicated plot asymmetry. Begg's test (p = 0.537) indicated no publication bias. |
| Ching, S. M. (2021)[64] | 99.78 | <0.001 | Considerable heterogeneity |  | NR - Authors used STROBE but did not report overall risk of bias. | Egger's test indicated plot asymmetry. |
| Adibi, A. (2021)[5] | 98.4 | <0.001 | Considerable heterogeneity |  | NR | Egger's test (p = 0.519) and Begg's test (p = 0.972) indicated "not considerable" publication bias. |
| Abdulla, E. K. (2021)[2] | 98 | <0.001 | Considerable heterogeneity | Tau2 = 402.44 Chi2 = 490.05 | NR - Authors use Downs and Black Checklist but do not report overall risk of bias. | Funnel plot inspection indicated asymmetry. |
| Varghese, A. (2021)[280] | 99.17 | <0.01 | Considerable heterogeneity | Q = 2467.18 | Moderate to high quality (Loney's 8-item Scale). | Egger's test (p = 0.13) indicated no publication bias. |
| Yan, H. (2021)[300] | 98 | <0.001 | Considerable heterogeneity |  | NR - Authors used STROBE but did not report overall risk of bias. | Funnel plot inspection and Begg’s rank test (p = 0.29) indicated no publication bias. |
| Marvaldi, M. (2021)[169] | 99.55 | <0.001 | Considerable heterogeneity |  | NR - Authors used AHRQ, NIH and Crombie’s Items but did not report overall risk of bias. | Egger's test (p > 0.1) and funnel plot inspection indicated no publication bias. |
| Zhao, Y. J. (2021)[311] | 98.77 | <0.001 | Considerable heterogeneity |  | Majority moderate quality (Loney's 8-item scale). | Egger’s test indicated marginal publication bias. |
| Al Maqbali, M. (2021)[10] | 99 | <0.000 | Considerable heterogeneity |  | Low to moderate risk of bias (NOS). | Funnel plot inspection indicated publication bias. Egger’s test (p = 0.29) indicated no publication bias. |
| Wu, T. (2021)[293] | 99.4 | NR | Considerable heterogeneity |  | Low to moderate risk of bias (STROBE). | Egger's test and funnel plot inspection indicated no publication bias. |
| Bareeqa, S.B. (2020)[30] | 98.53 | <0.001 | Considerable heterogeneity |  | Low to high risk of bias (NOS). | NR |
| Salazar de Pablo, G. (2020)[235] | 99.09 | <0.001 | Considerable heterogeneity | Q = 329.839 | NR - Authors used MMAT but did not report overall risk of bias. | NR |
| Anxiety – Pregnant and postpartum people | | | | | | |
| Demissie, D. B. (2021)[79] | 99.68 | 0.001 | Considerable heterogeneity |  | NR | Egger's test (p = 0.098) indicated plot asymmetry. |
| Shorey, S. Y. (2021)[252] | 100 | <0.001 | Considerable heterogeneity | Tau2 = 0.05  Chi squared = 2302.98, df = 11, p < 0.001, I2 = 100%. Test for overall effect Z = 6.22, p < 0.00001 | NR - Authors used JBI Checklist but did not report overall risk of bias. | Forest plot inspection indicated asymmetry. |
| Tomfohr-Madsen, L. M. (2021)[274] | 98.43 | <0.001 | Considerable heterogeneity | Q = 5780.54 | NR - Used NIH but did not report overall risk of bias. | Funnel plot inspection indicated symmetry. Egger's test (p = 0.01) indicated publication bias. |
| Yan, H. (2020)[299] | 99.4 | <0.001 | Considerable heterogeneity |  | NR - Authors used NOS but did not report overall risk of bias. | NR |
| Fan, S. (2020)[94] | 99.6 | <0.001 | Considerable heterogeneity |  | Majority high quality (JBI PACES). | Egger and Begg's tests indicated "no obvious publication bias". |
| Sun, F. (2020)[268] | 96 | <0.01 | Considerable heterogeneity | Tau2 = 0.0153 | High quality (AHRQ). | Forest plot inspection indicated asymmetry. |
| Anxiety – Vulnerable populations | | | | | | |
| Lee, K. W. (2022)[147] | 94.89 | <0.001 | Considerable heterogeneity |  | NR - Authors used STROBE but did not report overall risk of bias. | NR |
| Khraisat, B. R. (2022)[134] | 98 | <0.001 | Considerable heterogeneity | Q = 390.5 | NR | Funnel plot inspection indicated asymmetry. |
| Kuroda, N. (2021)[143] | 97 | <0.01 | Considerable heterogeneity | Tau2 = 0.0307 Chi2 = 604.22 | NR - Authors used CLARITY and JBI Checklist but did not report overall risk of bias. | Egger's test (0.48) revealed significant publication bias. |
| Anxiety – Young people | | | | | | |
| Li, W. (2022)[150] | 99.5 | <0.001 | Considerable heterogeneity |  | NR | Egger's test (p = 0.646) and Begg's test (p = 0.012) indicated plot asymmetry. |
| Zhang, Y. (2021)[310] | 100 | <0.001 | Considerable heterogeneity | Tau2 = 0.2526 | NR | Egger's test (p > 0.05) indicated publication bias. |
| Wang, C. (2021)[285] | 99.8 | <0.001 | Considerable heterogeneity |  | NR | Egger's test (p = 0.001) indicated publication bias. |
| Li, Y. (2021, Front. Psychol.)[323] | 99.9 | <0.001 | Considerable heterogeneity |  | NR - Authors used JBI but did not report overall risk of bias. | Egger's test (p = 0.308) and Begg's test (p = 0.871) indicated no publication bias. |
| Deng, J. (2021)[80] | 100 | <0.01 | Considerable heterogeneity |  | Low risk of bias (NOS). | Egger's test (p = 0.45) indicated no publication bias. |
| Chang, J. J. (2021)[58] | 99.9 | <0.001 | Considerable heterogeneity |  | Low risk of bias (NOS). | Egger's test (p = 0.620) indicated no publication bias. |
| Chai, J. (2021)[57] | 99 | <0.01 | Considerable heterogeneity | Tau2 = 0.0113 | NR | Egger's test (p = 0.26) indicated no publication bias. |
| Batra, K. (2021)[33] | 99.8 | <0.001 | Considerable heterogeneity |  | Fair to good quality (NIH). | Egger's test (p = 0.11) and funnel plot inspection indicated no publication bias. |
| Wu, T. (2021)[293] | 99.7 | NR | Considerable heterogeneity |  | Low to moderate risk of bias (STROBE). | Egger's test and funnel plot inspection indicated no publication bias. |
| Panda, P.K. (2020)[203] | 45 | 0.018 | Moderate heterogeneity |  | Low to moderate risk of bias (ROBINS-I). | Egger's test indicated no publication bias. |

| **Review** | **I2** | **p value** | **Heterogeneity level** | **Other heterogeneity statistics** | **Risk of Bias / Quality Appraisal** | **Publication bias** |
| --- | --- | --- | --- | --- | --- | --- |
| PTSD/PTSS – COVID-19 patients | | | | | | |
| Yunitri, N. (2021)[304] | 94 | NR | Considerable heterogeneity | Tau2 = 0.4695 Chi2 = 151.32 | Low to moderate risk of bias (Hoy's Risk of Bias Tool). | Peter's method indicated no publication bias (t = 0.22, p = 0.83). |
| Nagarajan, R. (2022)[183] | 87.95 | <0.001 | Considerable heterogeneity |  | NR - Authors used NOS but did not report overall risk of bias. | Egger's test revealed plot asymmetry (p = 0.07). |
| Dong, F. (2021, J. Affect. Disord)[84] | 99.8 | <0.001 | Considerable heterogeneity |  | NR | NR |
| Zhou, Y. (2021)[312] | NR | NR | NA |  | Low to high quality (Hoy's Risk of Bias Tool). | Funnel plot inspection revealed asymmetry. Egger's test (p = 0.41) indicated no publication bias. |
| PTSD/PTSS – General population | | | | | | |
| Yunitri, N. (2021)[304] | 99.8 | NR | Considerable heterogeneity | Tau2 = 1.2822 Chi2 = 12356.55 | Low to moderate risk of bias (Hoy's Risk of Bias Tool). | Peter's method indicated no publication bias (t = 0.22, p = 0.83). |
| Badenoch, J. B. (2022)[27] | 95.3 | NR | Considerable heterogeneity |  | NR | NR |
| Nochaiwong, S. (2021)[325] | 99.8 | <0.001 | Considerable heterogeneity | Chi2 = 96.87 | NR - Authors used Hoy's Risk of Bias Tool but did not report overall risk of bias. | Egger's test indicated no significant bias. |
| Fan, F. C. (2021)[93] | 99.676 | <0.001 | Considerable heterogeneity | Q = 5246.998 | NR | NR |
| Zhao, Y. J. (2021)[311] | 99.57 | <0.001 | Considerable heterogeneity |  | Majority moderate quality (Loney's 8-item scale). | Egger’s test indicated marginal publication bias. |
| Zhou, Y. (2021)[312] | NR | 0.115 | NA | Q = 4.334 | Low to high quality (Hoy's Risk of Bias Tool). | Funnel plot inspection revealed asymmetry. Egger's test (p = 0.41) indicated no publication bias. |
| Salehi, M. (2021)[236] | 98.53 | <0.001 | Considerable heterogeneity |  | Half low quality (STROBE). | Egger's test (p = 0.006) indicated publication bias. Begg's test indicated no publication bias (p = 0.13). |
| PTSD/PTSS – HCW | | | | | | |
| Yunitri, N. (2021)[304] | 99 | NR | Considerable heterogeneity | Tau2 = 1.1942 Chi2 = 3744.75 | Low to moderate risk of bias (Hoy's Risk of Bias Tool). | Peter's method indicated no publication bias (t = 0.22, p = 0.83). |
| Xiong, N. (2022)[296] | 99.97 | 0 | Considerable heterogeneity |  | NR | Egger's test (p = 0.23) indicated no publication bias. |
| Aymerich, C. (2022)[24] | 99.69 | <0.001 | Considerable heterogeneity |  | NR - Authors used NOS but did not report overall risk of bias. | Egger's test - Results NR. |
| Norhayati, M. N. (2021)[191] | 99 | <0.001 | Considerable heterogeneity |  | NR - Authors used JBI Checklist but did not report overall risk of bias. | Funnel plots - Results NR. |
| Mulyadi, M. (2021)[181] | NR | NR | NA |  | Low risk of bias (JBI Checklist). | NR |
| Hao, Q. (2021)[109] | 99 | <0.01 | Considerable heterogeneity |  | NR | NR |
| Varghese, A. (2021)[280] | 96.43 | <0.001 | Considerable heterogeneity | Tau2 = 0.04 Q = 56.02 | Moderate to high quality (Loney's 8-item Scale). | Egger's test (p = 0.47) indicated no publication bias. |
| Marvaldi, M. (2021)[169] | 99.62 | NR | Considerable heterogeneity |  | NR - Authors used AHRQ, NIH and Crombie’s Items but did not report overall risk of bias. | Egger's test (p > 0.1) and funnel plot inspection indicated no publication bias. |
| Zhao, Y. J. (2021)[311] | 99.59 | <0.001 | Considerable heterogeneity |  | Majority moderate quality (Loney's 8-item scale). | Egger’s test indicated marginal publication bias. |
| Zhou, Y. (2021)[312] | NR | <0.001 | NA | Q = 24.368 | Low to high quality (Hoy's Risk of Bias Tool). | Funnel plot inspection indicated asymmetry. Egger's test (p = 0.41) indicated no publication bias. |
| Salehi, M. (2021)[236] | 94.93 | <0.001 | Considerable heterogeneity |  | Half low quality (STROBE). | Begg`s test (z= 1.73, p= 0.083) indicated no publication bias. Egger's test (p = 0.011) indicated publication bias. |
| Salazar de Pablo, G. (2020)[235] | NR | NR | NA |  | NR - Authors used MMAT but did not report overall risk of bias. | NR |
| PTSD/PTSS – Young people | | | | | | |
| Batra, K. (2021)[33] | 99.8 | <0.001 | Considerable heterogeneity |  | Fair to good quality (NIH). | Funnel plot inspection and Egger's test (p = 0.78) indicated no publication bias. |

| **Review** | **I2** | **p value** | **Heterogeneity level** | **Other heterogeneity statistics** | **Risk of Bias / Quality Appraisal** | **Publication bias** |
| --- | --- | --- | --- | --- | --- | --- |
| Stress – General population | | | | | | |
| Nochaiwong, S. (2021)[325] | 99.8 | <0.001 | Considerable heterogeneity | Chi2 = 72.93 | NR - Authors used Hoy's Risk of Bias Tool but did not report overall risk of bias. | Egger's test indicated no publication bias. |
| Li, W. (2021)[149] | 99.81 | <0.001 | Considerable heterogeneity |  | NR - Authors used STROBE but did not report overall risk of bias. | NR |
| Bareeqa, S.B. (2020)[30] | 99.86 | <0.001 | Considerable heterogeneity |  | Low to high risk of bias (NOS). | NR |
| Salari, N. (2020, Glob. Health)[233] | 96.8 | <0.001 | Considerable heterogeneity |  | Medium to high quality (STROBE). | Egger's test (p = 0.304) indicated no publication bias. |
| Stress – HCW | | | | | | |
| Aymerich, C. (2022)[24] | 99.87 | <0.001 | Considerable heterogeneity |  | NR - Authors used NOS but did not report overall risk of bias. | Egger's test - Results NR. |
| Zhang, H. (2021)[329] | 99.5 | NR | Considerable heterogeneity |  | "Poor to good quality" (NIH). | NR - Study numbers too low for Egger's test. |
| Norhayati, M. N. (2021)[191] | 100 | <0.001 | Considerable heterogeneity |  | NR - Authors used JBI Checklist but did not report overall risk of bias. | Funnel plots - Results NR. |
| Mulyadi, M. (2021)[181] | 98 | <0.001 | Considerable heterogeneity | Q = 123.54 | Low risk of bias (JBI). | Egger's test (p = 0.16) indicated no publication bias. |
| Mahmud, S. (2021)[164] | 99.78 | <0.001 | Considerable heterogeneity | Tau2 = 658.32 Q = 40900.83 H2 = 448.13 | NR | Egger's test (Z = 0.33, p = 0.74) indicated no publication bias. |
| Dong, F. (2021, Front. Psychol.)[83] | 96.9 | <0.001 | Considerable heterogeneity |  | NR | Egger's test (p = 0.000) indicated plot asymmetry. Begg's test (p = 0.602) indicated no publication bias. |
| Ching, S. M. (2021)[64] | 99.95 | <0.001 | Considerable heterogeneity |  | NR - Authors used STROBE but did not report overall risk of bias. | Egger's test revealed plot asymmetry. |
| Abdulla, E. K. (2021)[2] | 99 | <0.001 | Considerable heterogeneity | Tau2 = 535.92 Chi2 = 1197.50 | NR - Authors used Downs and Black Checklist but did not report overall risk of bias. | Funnel plot revealed plot asymmetry. |
| Varghese, A. (2021)[280] | 98.59 | <0.001 | Considerable heterogeneity | Q = 636.58 | Moderate to high quality (Loney's 8-item Scale). | Egger's test (p = 0.52) indicated no publication bias. |
| Marvaldi, M. (2021)[169] | 99.56 | <0.001 | Considerable heterogeneity |  | NR - Authors used AHRQ, NIH and Crombie’s Items but did not report overall risk of bias. | Egger's test (p > 0.1) and funnel plot inspection indicated no publication bias. |
| Al Maqbali, M. (2021)[10] | 98 | <0.000 | Considerable heterogeneity |  | Low to moderate risk of bias (NOS). | Funnel plot inspection indicated publication bias. Egger’s test (p = 0.42) indicated no publication bias. |
| Stress – Pregnant and postpartum people | | | | | | |
| Demissie, D. B. (2021)[79] | 98.8 | <0.001 | Considerable heterogeneity |  | NR | NR |
| Stress – Young people | | | | | | |
| Wang, C. (2021)[285] | 99.1 | <0.001 | Considerable heterogeneity |  | NR | Egger's test (p = 0.507) indicated no publication bias. |
| Batra, K. (2021)[33] | 98.9 | <0.001 | Considerable heterogeneity |  | Fair to good quality (NIH). | Funnel plot inspection and Egger's test (p = 0.68) indicated no publication bias. |

| **Review** | **I2** | **p value** | **Heterogeneity level** | **Other heterogeneity statistics** | **Risk of Bias / Quality Appraisal** | **Publication bias** |
| --- | --- | --- | --- | --- | --- | --- |
| Psychological distress – General population | | | | | | |
| Wu, T. (2021)[293] | 97.2 | <0.01 | Considerable heterogeneity |  | Low to moderate risk of bias (STROBE). | Egger's test and funnel plot inspection indicated no publication bias. |
| Nochaiwong, S. (2021)[325] | 99.7 | <0.001 | Considerable heterogeneity | Chi2 = 82.14 | NR - Authors used Hoy's Risk of Bias tool but did not report overall risk of bias. | Egger's test indicated no significant bias. |
| Psychological distress – HCW | | | | | | |
| Salazar de Pablo, G. (2020)[235] | 99.744 | <0.001 | Considerable heterogeneity | Q = 780.733 | NR - Authors used MMAT but did not report overall risk of bias. | NR |
| Wu, T. (2021)[293] | 99.8 | NR | Considerable heterogeneity |  | Low to moderate risk of bias (STROBE). | Egger's test and funnel plot inspection indicated no publication bias. |
| Psychological distress – Pregnant and postpartum people | | | | | | |
| Yan, H. (2020)[299] | 76.7 | 0.01 | Considerable heterogeneity |  | NR - Authors used NOS but did not report overall risk of bias. | NR |

## S13 Table. Model results including mixed and quarantined populations

|  | **k** | **Pooled prevalence** | **95% CI** | **Prediction interval** | **Tau^2^** | **I^2^** | **QE** |
| --- | --- | --- | --- | --- | --- | --- | --- |
| **Master models** | | | | | | | |
| Depression | 81 | 0.2838 | 0.26, 0.31 | 0.13, 0.52 | 0.25 | 99.97% | 111080.04(<.01) |
| Anxiety | 70 | 0.3089 | 0.28, 0.34 | 0.13, 0.58 | 0.33 | 99.98% | 101490.18 (<.01) |
| Stress | 18 | 0.3910 | 0.34, 0.44 | 0.21, 0.60 | 0.19 | 99.91% | 4970.25 (<.01) |
| Psych Distress | 6 | 0.3759 | 0.24, 0.54 | 0.09, 0.78 | 0.69 | 99.98% | 21149.74 (<.01) |
| PTSD/PTSS | 27 | 0.1969 | 0.16, 0.23 | 0.07, 0.44 | 0.35 | 99.91% | 9624.56 (<.01) |

## S14 Table. Heterogeneity and risk of bias information for pooled estimates included in meta-review of standardised mean difference

| **Review** | **Population** | **I2** | **p value** | **Heterogeneity level** | **Other heterogeneity statistics** | **Risk of Bias / Quality Appraisal** | **Publication bias** |
| --- | --- | --- | --- | --- | --- | --- | --- |
| **Depression** | | | | | | | |
| Min, S. (2021)[173] | General population | 97.6 | <0.001 | Considerable heterogeneity | Q = 319.3 | NR | Egger's test (p = 0.20) indicated no publication bias. |
| Kunzler, A. (2021)[142] | General population | 100 | 0.03 | Considerable heterogeneity | Tau2 = 1.79 Chi2 = 16430.33 | Fair to poor quality (NIH). | NR |
| Kunzler, A. (2021)[142] | HCWs | 97 | NR | Considerable heterogeneity |  | Fair to poor quality (NIH). | NR |
| Robinson, E. (2022)[222] | Mixed | 95 | <0.001 | Considerable heterogeneity |  | NR | Egger's test indicated no publication bias. |
| Kunzler, A. (2021)[142] | Patients - COVID-19 and other | 98 | NR | Considerable heterogeneity |  | Fair to poor quality (NIH). | NR |
| Chmielewska, B. (2021)[318] | Pregnant and postpartum people | 79 | <0.01 | Considerable heterogeneity | Tau2 = 0.0792 Chi2 = 9.73 | Moderate quality (NOS). | Funnel plot inspection indicated no publication bias (p = 0.12). |
| Hessami, K. (2020)[112] | Pregnant and postpartum people | 98 | 0 | Considerable heterogeneity |  | NR | Egger and Begg's statistics indicated no publication bias (p > 0.05). |
| Yan, H. (2020)[299] | Pregnant and postpartum people | 56.8 | 0.128 | Substantial Heterogeneity |  | Low risk of bias (NOS). | NR |
| Altieri, M. (2022)[18] | Vulnerable populations | 94.62 | <0.001 | Considerable heterogeneity | Q = 111.6 | NR | Egger's test (p = 0.080) indicated no publication bias. |
| **Anxiety** | | | | | | | |
| Min, S. (2021)[173] | General population | 97.7 | <0.001 | Considerable heterogeneity | Q = 317.6 | NR | Egger's regression test showed no significant publication bias (p = 0.71). |
| Kunzler, A. (2021)[142] | General population | 99 | <0.01 | Considerable heterogeneity | Tau2 = 0.4 Chi2 = 4995.25 | Fair to poor quality (NIH). | NR |
| Kunzler, A. (2021)[142] | HCWs | 99 | NR | Considerable heterogeneity |  | Fair to poor quality (NIH). | NR |
| Robinson, E. (2022)[222] | Mixed | 96.2 | 0.021 | Considerable heterogeneity |  | NR | Egger's test revealed no publication bias. |
| Kunzler, A. (2021)[142] | Patients - COVID-19 and other | 93 | NR | Considerable heterogeneity |  | Fair to poor quality (NIH). | NR |
| Hessami, K. (2020)[112] | Pregnant and postpartum people | 90.2 | 0 | Considerable heterogeneity |  | NR | Egger and Begg's statistics reported no publication bias (p > 0.05). |
| Yan, H. (2020)[299] | Pregnant and postpartum people | 0 | 0.506 | Low heterogeneity |  | Low risk of bias (NOS). | NR |
| Altieri, M. (2022)[18] | Vulnerable populations | 95.67 | <0.001 | Considerable heterogeneity | Q = 115.61 | NR | Egger's test reported no significant publication bias (p = 0.265). |

| **Review** | **Outcome** | **Population** | **I2** | **p value** | **Heterogeneity level** | **Other heterogeneity statistics** | **Risk of Bias / Quality Appraisal** | **Publication bias** |
| --- | --- | --- | --- | --- | --- | --- | --- | --- |
| **Other mental health conditions** | | | | | | | | |
| Min, S. (2021)[173] | Mental ill-health | General population | 99.2 | <0.001 | Considerable heterogeneity | Q = 648 | NR | Egger's test (p = 0.47) indicated no publication bias. |
| Kunzler, A. (2021)[142] | Stress | General population | 100 | NR | Considerable heterogeneity |  | Fair to poor quality (NIH). | NR |
| Kunzler, A. (2021)[142] | Stress | HCWs | 99 | NR | Considerable heterogeneity |  | Fair to poor quality (NIH). | NR |
| Robinson, E. (2022)[222] | Psychosis | Mixed | 73.1 | 0.12 | Considerable heterogeneity |  | NR | Egger's test indicated no publication bias. |
| Robinson, E. (2022)[222] | Mental ill-health | Mixed | 97.8 | 0.002 | Considerable heterogeneity |  | NR | Egger's test indicated no publication bias. |
| Kunzler, A. (2021)[142] | Stress | Patients - COVID-19 and other | 98 | NR | Considerable heterogeneity |  | Fair to poor quality (NIH). | NR |
| BussiËres, E. (2021)[46] | Other | Young people | 98.02 | < 0.001 | Considerable heterogeneity | Q = 1008807 | NR | Forest plot inspection indicated no publication bias. |

## S1 Figure. Forest plot of supplementary model of probable depression pooled prevalence


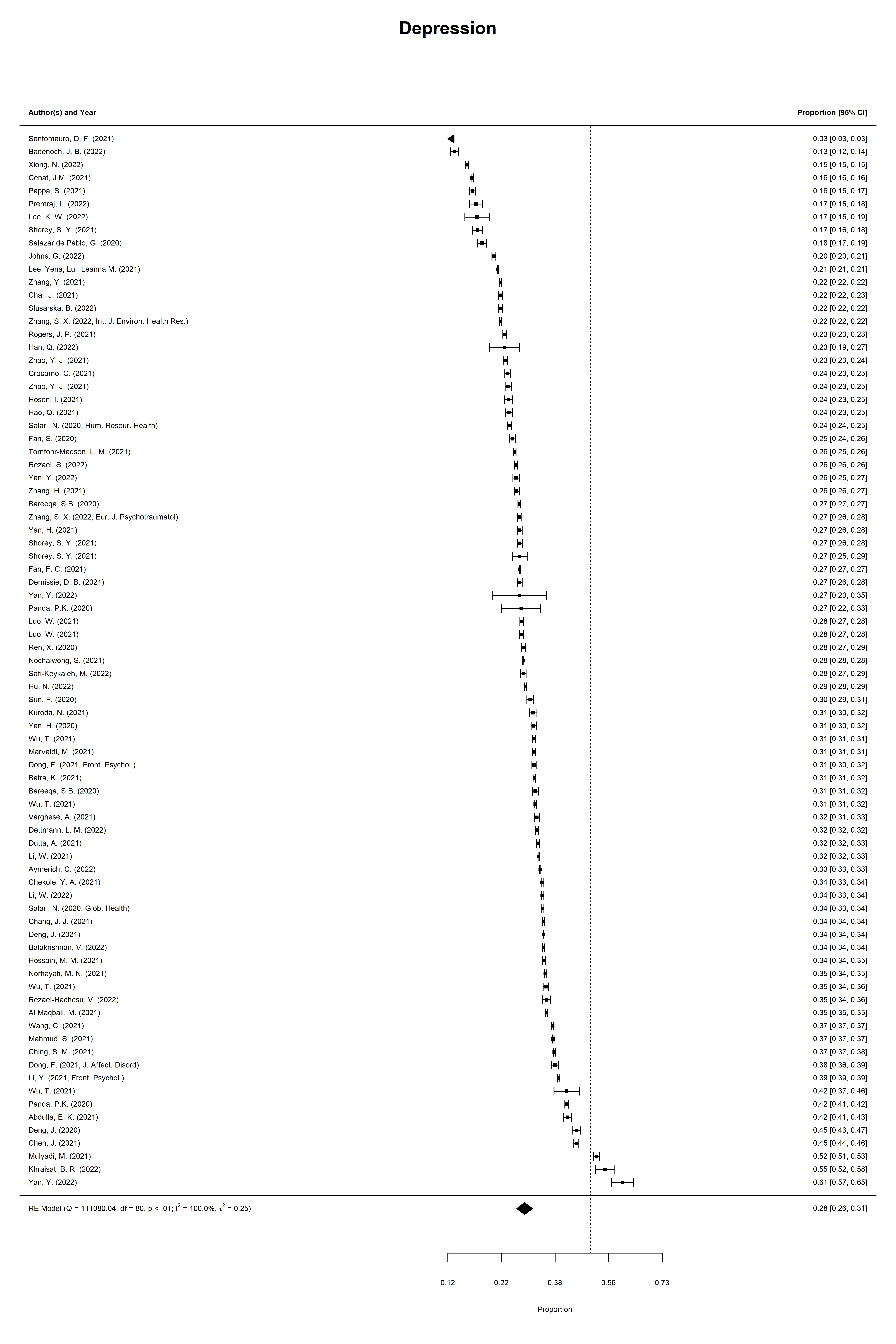


## S2 Figure. Forest plot of supplementary model of probable anxiety pooled prevalence


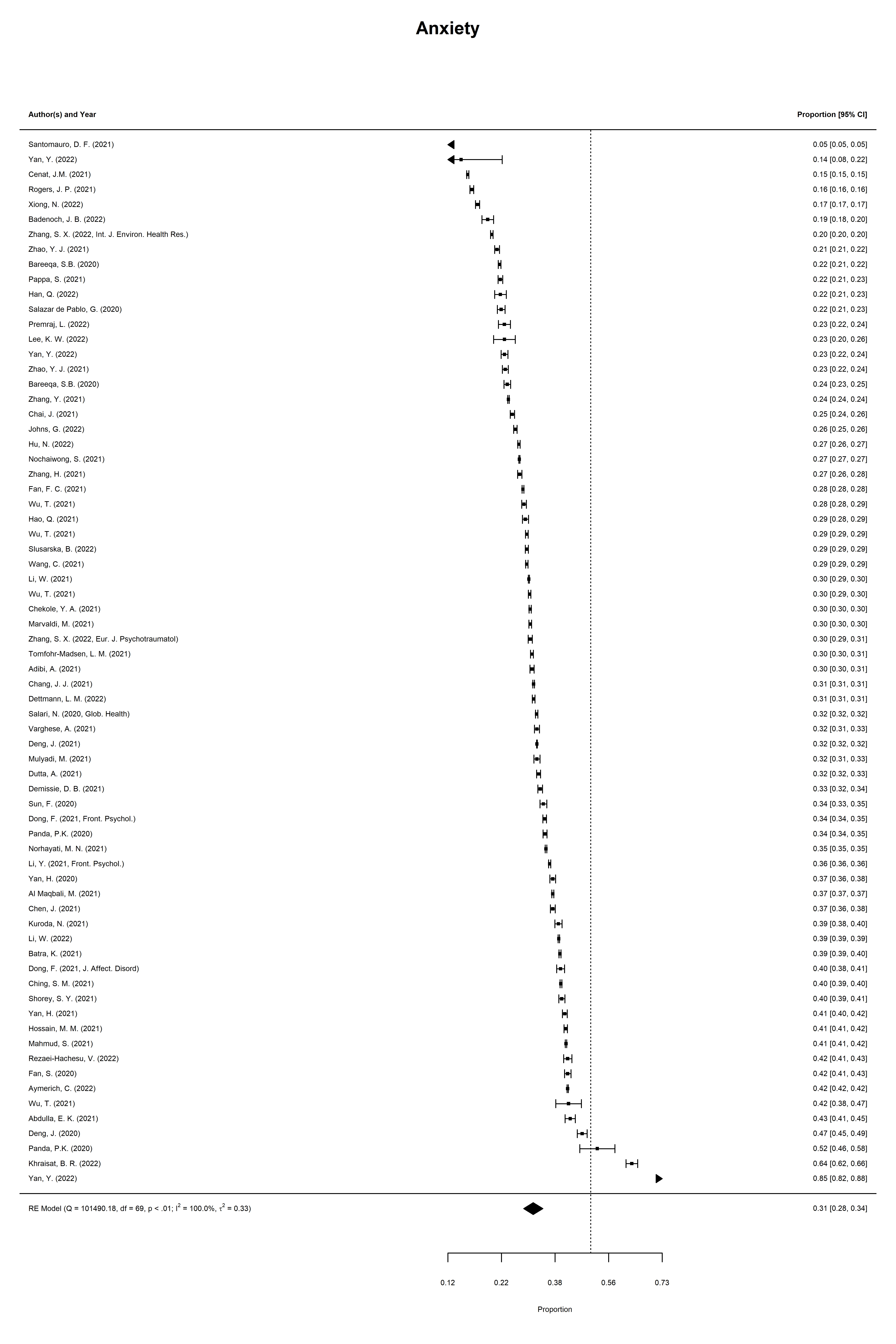


S3 Figure. Forest plot of supplementary model of stress pooled prevalence (no change to main model due to no additional reviews.
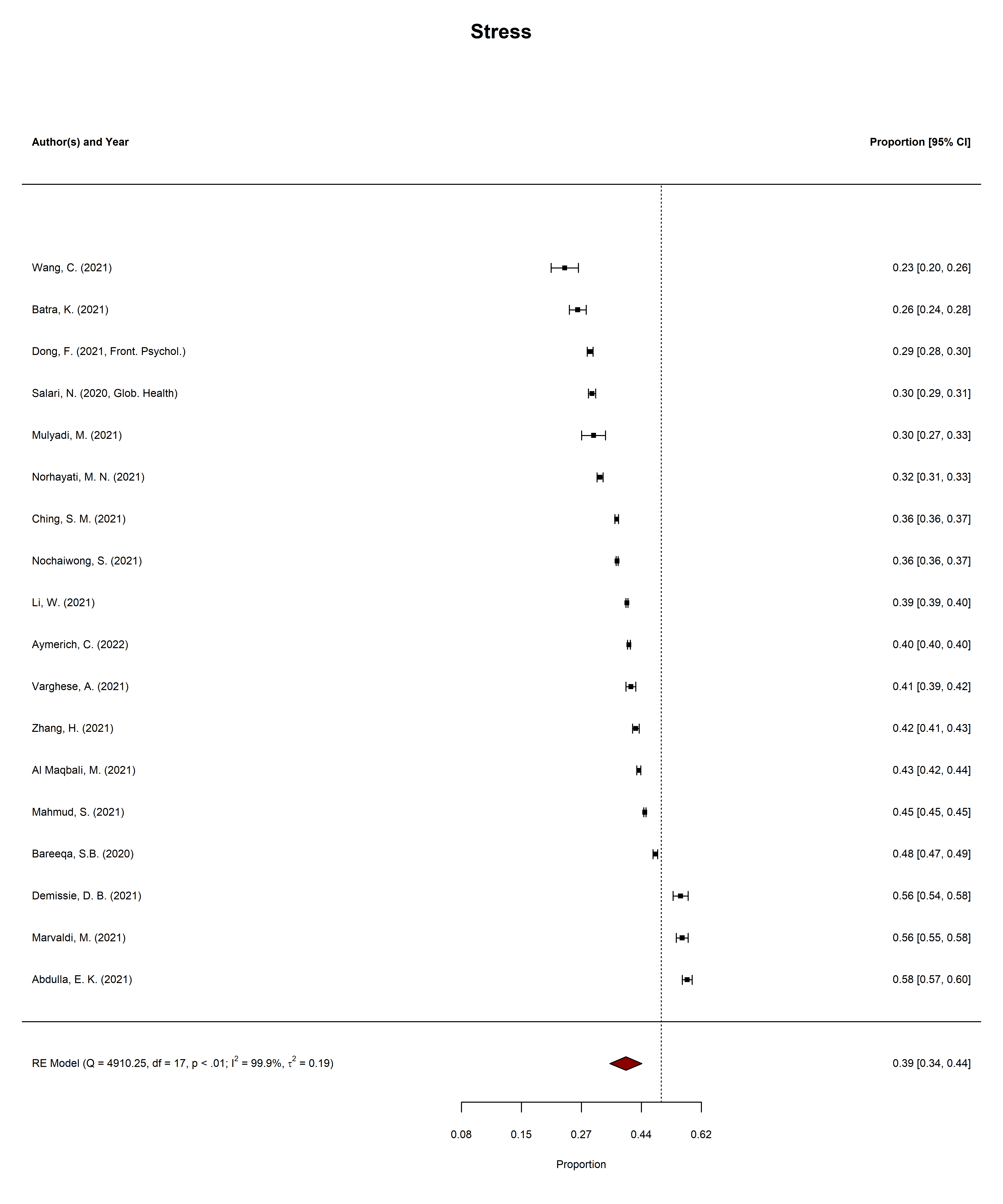


## S4 Figure. Forest plot of supplementary model of PTSD pooled prevalence


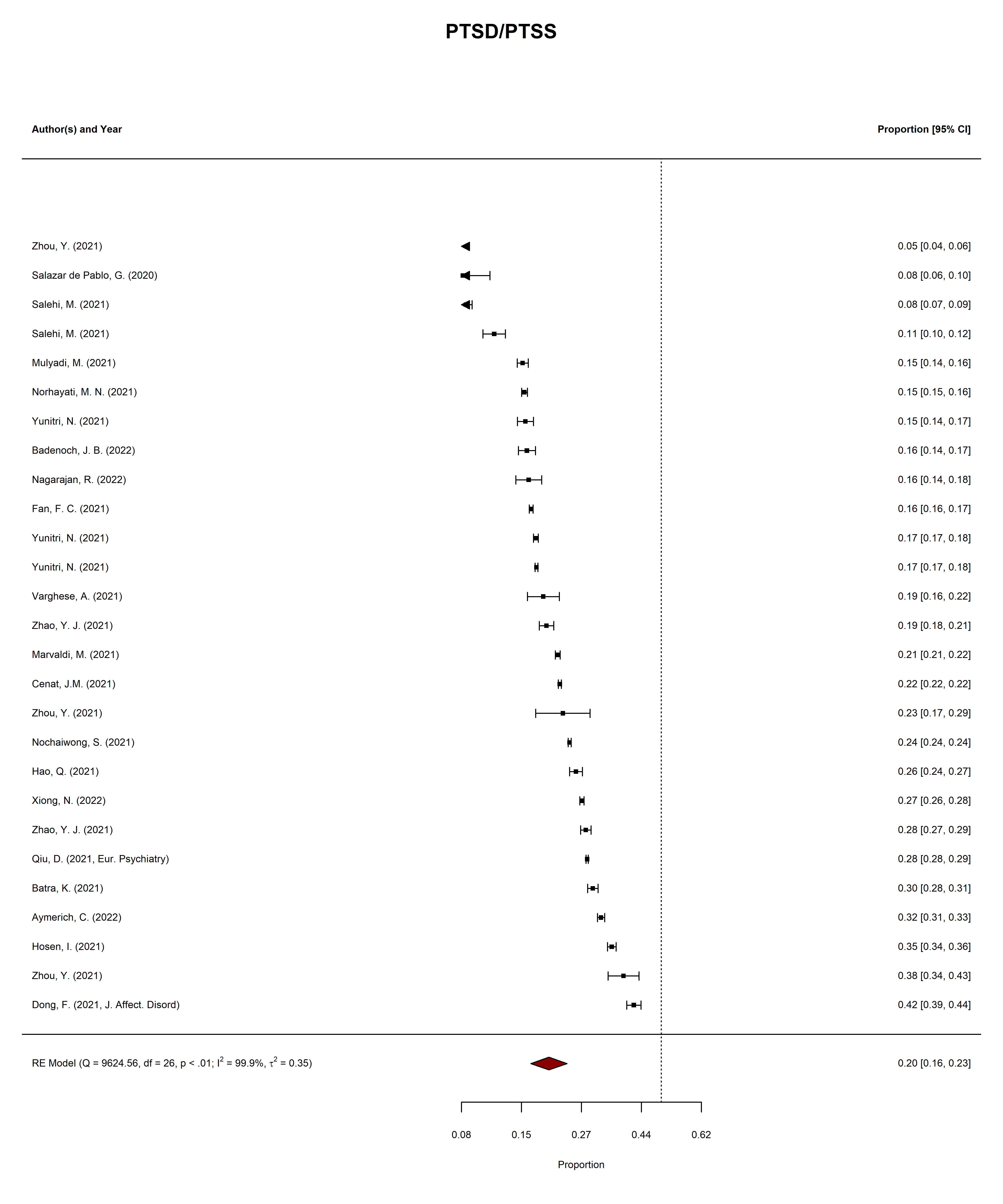


## S5 Figure. Forest plot of supplementary model of psychological distress pooled prevalence


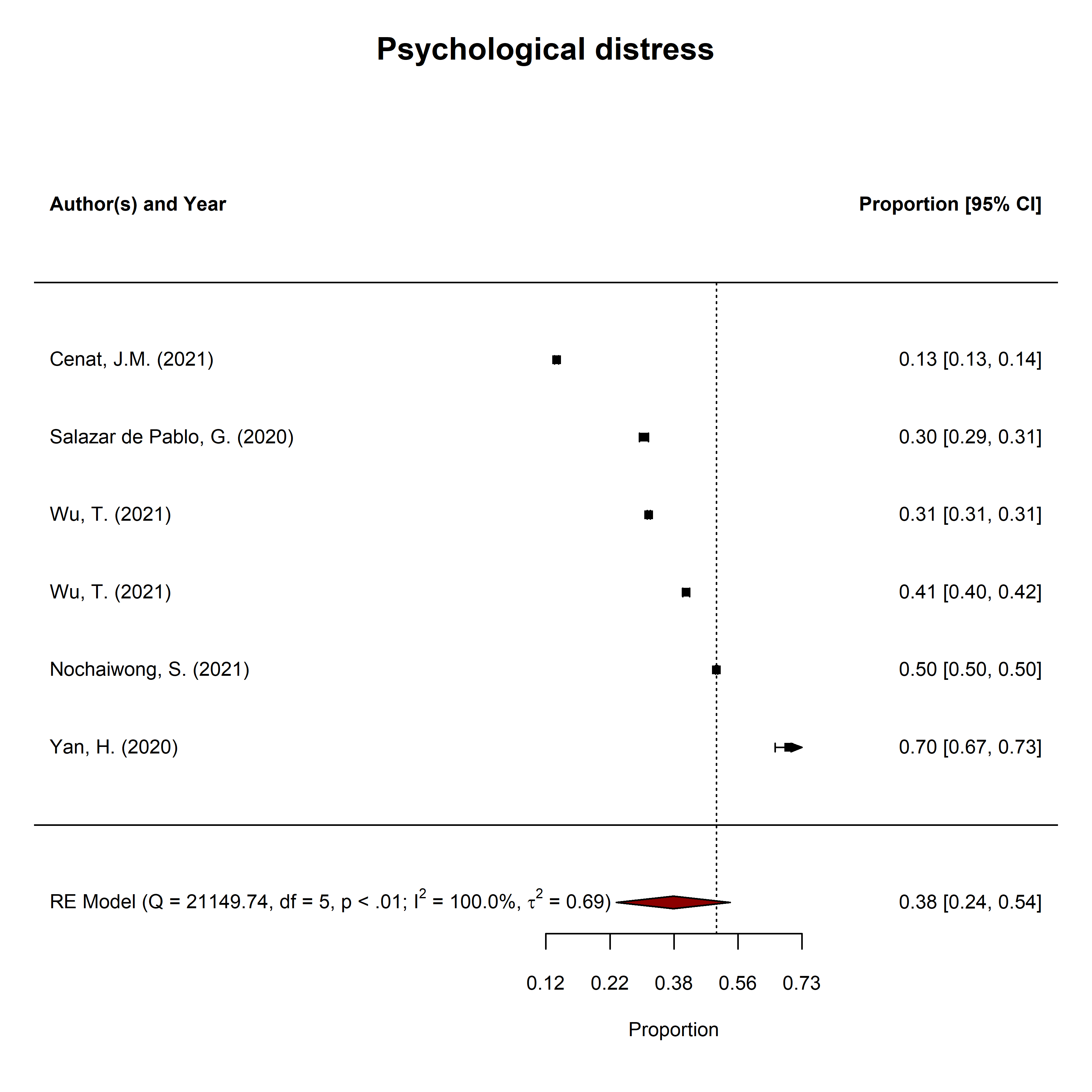


## S1 Results. Further narrative synthesis detail on healthcare workers

Other HCW occupational factors found to be associated with mental ill-health included having poor cohesion across health sectors[331]; perceiving stigma around disclosing about one’s work and negative public attitudes[319]; and lacking appropriate training, PPE, and information[319, 331]. There were mixed effects reported about the impact of clinician type (e.g., technician and physician)[319] and level of work experience or seniority on mental health[316, 317, 321, 331]. Hours worked or part-time work status was not associated with poorer mental health[317].

Demographic characteristics associated with poorer mental health amongst HCW included being female[317], younger[319], older[319, 328, 331], single[317, 319], married [319], married with children[319], having a lower educational level[317] and existing physical illness[319].

Psychological characteristics associated with poor mental health amongst HCW included having a history of mental illness[317], PTSD symptoms[317] and use of negative coping[319]. Protective psychological factors included self-efficacy[317], resilience[319], coping ability[317], altruism[317] and receiving psychiatric telehealth support[328] or other mental health services[317]. Problem-focused adaptive strategy was not associated with mental health[328]. A meta-analysis of global studies assessing found that HCW showed a significantly higher association between fear of COVID-19 and stress (.41 vs .76, *I^2^*= NR) and depression (.37 vs .68, *I^2^*=95.2) than the general population[330].

Having COVID-19 symptoms[317], being hospitalised with COVID-19[321] and spending time in quarantine[319] were associated with poorer mental health outcomes amongst HCW.

## S1 References. Complete list of references

1. Abasıyanık Z, Kurt M, Kahraman T. COVID-19 and Physical Activity Behaviour in People with Neurological Diseases: A Systematic Review. Journal of Developmental and Physical Disabilities. 2022.

2. Abdulla EK, Velladath SU, Varghese A, Anju M. Depression and anxiety associated with COVID- 19 pandemic among healthcare professionals in India- A systematic review and meta-analysis. Clinical Epidemiology and Global Health. 2021;12.

3. Al Moaleem MM. Anxiety during COVID-19 among Saudi Arabian Population: A Systematic Review. World Journal of Dentistry. 2021;13(1):87-94.

4. Acuff SF, Strickland JC, Tucker JA, Murphy JG. Changes in alcohol use during COVID-19 and associations with contextual and individual difference variables: A systematic review and meta-analysis. Psychology of Addictive Behaviors. 2022;36:1-19.

5. Adibi A, Golitaleb M, Farrahi-Ashtiani I, Pirani D, Yousefi K, Jamshidbeigi Y, et al. The Prevalence of Generalized Anxiety Disorder Among Health Care Workers During the COVID-19 Pandemic: A Systematic Review and Meta-Analysis. Frontiers in Psychiatry. 2021;12.

6. Ahmad M, Shaik R, Ahmad R, Yusuf M, Khan M, Almutairi A, et al. "Long Covid”: An Insight. Eur Rev Med Pharmacol Sci. 2021;25:5561-77.

7. Ahmadi Hekmatikar AH, Ferreira Júnior JB, Shahrbanian S, Suzuki K. Functional and Psychological Changes after Exercise Training in Post-COVID-19 Patients Discharged from the Hospital: A PRISMA-Compliant Systematic Review. International Journal of Environmental Research and Public Health. 2022;19(4):2290.

8. Al Falasi B, Al Mazrouei M, Al Ali M, Al Dhamani M, Al Ali A, Al Kindi M, et al. Prevalence and Determinants of Immediate and Long-Term PTSD Consequences of Coronavirus-Related (CoV-1 and CoV-2) Pandemics among Healthcare Professionals: A Systematic Review and Meta-Analysis. International Journal of Environmental Research & Public Health [Electronic Resource]. 2021;18(4):23.

9. Al Mamun F, Hosen I, Misti JM, Kaggwa MM, Mamun MA. Mental disorders of Bangladeshi students during the COVID-19 pandemic: a systematic review. Psychology Research and Behavior Management. 2021;14:645.

10. Al Maqbali M, Al Sinani M, Al-Lenjawi B. Prevalence of stress, depression, anxiety and sleep disturbance among nurses during the COVID-19 pandemic: A systematic review and meta-analysis. Journal of Psychosomatic Research. 2021;141:110343.

11. Alaradi A, Irum S, Ebrahim N, Mohamed FMJ, Hazeem FMJ, Ashfaq M. The Mental Health Impact of COVID-19 Pandemic on Health Care Workers and Coping Strategies: A Systematic Literature Review. International Journal of Online & Biomedical Engineering. 2021;17(9).

12. Alimoradi Z, Broström A, Tsang HWH, Griffiths MD, Haghayegh S, Ohayon MM, et al. Sleep problems during COVID-19 pandemic and its&#x2019; association to psychological distress: A systematic review and meta-analysis. eClinicalMedicine. 2021;36.

13. Abedi N. Psychological effects of the COVID-19 pandemic on dentistry: A systematic review study. Journal of Education and Health Promotion. 2021;10.

14. Allan SM, Bealey R, Birch J, Cushing T, Parke S, Sergi G, et al. The prevalence of common and stress-related mental health disorders in healthcare workers based in pandemic-affected hospitals: a rapid systematic review and meta-analysis. European Journal of Psychotraumatology. 2020;11(1):1810903.

15. Almeida ILdL, Rego JF, Teixeira ACG, Moreira MR. Social isolation and its impact on child and adolescent development: a systematic review. Revista Paulista de Pediatria. 2021;40.

16. Almqvist J, Granberg T, Tzortzakakis A, Klironomos S, Kollia E, Ohberg C, et al. Neurological manifestations of coronavirus infections - a systematic review. Annals of Clinical & Translational Neurology. 2020;7(10):2057-71.

17. Alonso-Esteban Y, López-Ramón MF, Moreno-Campos V, Navarro-Pardo E, Alcantud-Marín F. A Systematic Review on the Impact of the Social Confinement on People with Autism Spectrum Disorder and Their Caregivers during the COVID-19 Pandemic. Brain Sciences. 2021;11(11):1389.

18. Altieri M, Capuano R, Bisecco A, D'Ambrosio A, Buonanno D, Tedeschi G, et al. Psychological consequences of COVID-19 pandemic on people with multiple sclerosis: A meta-analytic study. Journal of the Neurological Sciences. 2021;429.

19. Alzahrani F, Alshahrani NZ, Abu Sabah A, Zarbah A, Abu Sabah S, Mamun MA. Prevalence and factors associated with mental health problems in Saudi general population during the coronavirus disease 2019 pandemic: A systematic review and meta-analysis. PsyCh Journal. 2022;11(1):18-29.

20. Amanullah S, Ramesh Shankar R. The Impact of COVID-19 on Physician Burnout Globally: A Review. Healthcare. 2020;8(4):22.

21. Amorós-Reche V, Belzunegui-Pastor À, Hurtado G, Espada JP. Emotional Problems in Spanish Children and Adolescents during the COVID-19 Pandemic: A Systematic Review. Clinical and Health. 2022;33(1):19-28.

22. Araujo LAD, Veloso CF, Souza MDC, Azevedo JMCD, Tarro G. The potential impact of the COVID-19 pandemic on child growth and development: a systematic review. Jornal de Pediatria. 2020.

23. Arora T, Grey I, Ostlundh L, Lam KBH, Omar OM, Arnone D. The prevalence of psychological consequences of COVID-19: A systematic review and meta-analysis of observational studies. Journal of Health Psychology. 2020:1359105320966639.

24. Aymerich C, Pedruzo B, Pérez JL, Laborda M, Herrero J, Blanco J, et al. COVID-19 pandemic effects on health worker’s mental health: Systematic review and meta-analysis. European Psychiatry. 2022;65(1):e10.

25. Sulistia Ayu NM, Novieastari E, Gayatri D, Handiyani H, Arruum D. Personal Protective Equipment and Nurse Self-efficacy due to Coronavirus Disease-19 Pandemic: A Systematic Review. Open Access Macedonian Journal of Medical Sciences. 2021;9(G):195-202.

26. Ayubi E, Bashirian S, Khazaei S. Depression and Anxiety Among Patients with Cancer During COVID-19 Pandemic: A Systematic Review and Meta-analysis. Journal of Gastrointestinal Cancer. 2021;05:05.

27. Badenoch JB, Rengasamy ER, Watson C, Jansen K, Chakraborty S, Sundaram RD, et al. Persistent neuropsychiatric symptoms after COVID-19: a systematic review and meta-analysis. Brain Communications. 2021;4(1).

28. Balakrishnan V, Ng KS, Kaur W, Govaichelvan K, Lee ZL. COVID-19 depression and its risk factors in Asia Pacific – A systematic review and meta-analysis. Journal of Affective Disorders. 2022;298:47-56.

29. Banerjee D, Vaishnav M, Sathyanarayana Rao TS, Raju MSVK, Dalal PK, Javed A, et al. Impact of the COVID-19 pandemic on psychosocial health and well-being in South-Asian (World Psychiatric Association zone 16) countries: A systematic and advocacy review from the Indian Psychiatric Society. Indian Journal of Psychiatry. 2020;62(9 Supplement 3):S343-S53.

30. Bareeqa SB, Ahmed SI, Samar SS, Yasin W, Zehra S, Monese GM, et al. Prevalence of depression, anxiety and stress in china during COVID-19 pandemic: A systematic review with meta-analysis. International Journal of Psychiatry in Medicine. 2020:91217420978005.

31. Basnayake O, Jayarajah U, Gunawardena K, Samarasekera DN. Impact of COVID-19 on postgraduate education and mental wellbeing of surgical trainees: a systematic review. Sri Lanka Journal of Surgery. 2021;39(3).

32. Batista P, Duque V, Luzio-Vaz A, Pereira A. Anxiety impact during COVID-19: a systematic review. Journal of Infection in Developing Countries. 2021;15(3):320-5.

33. Batra K, Sharma M, Batra R, Singh TP, Schvaneveldt N. Assessing the Psychological Impact of COVID-19 among College Students: An Evidence of 15 Countries. Healthcare. 2021;9(2):17.

34. Behrmann JT, Blaabjerg J, Jordansen J, Jensen de López KM. Systematic Review: Investigating the Impact of COVID-19 on Mental Health Outcomes of Individuals With ADHD. Journal of Attention Disorders. 2021;26(7):959-75.

35. Bekele F, Hajure M. Magnitude and determinants of the psychological impact of COVID-19 among health care workers: A systematic review. SAGE Open Medicine. 2021;9:20503121211012512.

36. Bekele F, Mechessa DF, Sefera B. Prevalence and associated factors of the psychological impact of COVID-19 among communities, health care workers and patients in Ethiopia: A systematic review. Annals of Medicine and Surgery. 2021;66:102403.

37. Berger E, Jamshidi N, Reupert A, Jobson L, Miko A. Review: The mental health implications for children and adolescents impacted by infectious outbreaks - a systematic review. Child & Adolescent Mental Health. 2021;26(2):157-66.

38. Billings J, Ching BCF, Gkofa V, Greene T, Bloomfield M. Experiences of frontline healthcare workers and their views about support during COVID-19 and previous pandemics: a systematic review and qualitative meta-synthesis. BMC Health Services Research. 2021;21(1):923.

39. Blasco-Belled A, Tejada-Gallardo C, Fatsini-Prats M, Alsinet C. Mental health among the general population and healthcare workers during the COVID-19 pandemic: A meta-analysis of well-being and psychological distress prevalence. Current Psychology. 2022.

40. Boden M, Cohen N, Froelich JM, Hoggatt KJ, Abdel Magid HS, Mushiana SS. Mental disorder prevalence among populations impacted by coronavirus pandemics: A multilevel meta-analytic study of COVID-19, MERS & SARS. General Hospital Psychiatry. 2021;70:124-33.

41. Bourmistrova NW, Solomon T, Braude P, Strawbridge R, Carter B. Long-term effects of COVID-19 on mental health: A systematic review. Journal of Affective Disorders. 2022;299:118-25.

42. Budiman AA, Lestari R, Yuliatun L. What is factor contributing to medical student learning anxiety during COVID-19 pandemic? A systematic review. International Journal of Public Health. 2021;10(4):898-905.

43. Buecker S, Horstmann KT. Loneliness and Social Isolation During the COVID-19: A systematic review enriched with empirical evidence from a large-scale diary study. European Psychologist. 2021;26(4):272-84.

44. Bueno-Notivol J, Gracia-Garcia P, Olaya B, Lasheras I, Lopez-Anton R, Santabarbara J. Prevalence of depression during the COVID-19 outbreak: A meta-analysis of community-based studies. International Journal of Clinical & Health Psychology. 2021;21(1):100196.

45. Busch IM, Moretti F, Mazzi M, Wu AW, Rimondini M. What We Have Learned from Two Decades of Epidemics and Pandemics: A Systematic Review and Meta-Analysis of the Psychological Burden of Frontline Healthcare Workers. Psychotherapy & Psychosomatics. 2021;90(3):178-90.

46. Bussières E-L, Malboeuf-Hurtubise C, Meilleur A, Mastine T, Hérault E, Chadi N, et al. Consequences of the COVID-19 Pandemic on Children's Mental Health: A Meta-Analysis. Frontiers in Psychiatry. 2021;12.

47. V CF, Iarocci G. Child and Family Outcomes Following Pandemics: A Systematic Review and Recommendations on COVID-19 Policies. Journal of Pediatric Psychology. 2020;45(10):1124-43.

48. Cabarkapa S, Nadjidai SE, Murgier J, Ng CH. The psychological impact of COVID-19 and other viral epidemics on frontline healthcare workers and ways to address it: A rapid systematic review. Brain, Behavior, & Immunity Health. 2020;8:100144.

49. Caffieri A, Margherita G. The psychological impact of COVID-19 on women’s wellbeing during pregnancy and postpartum one year after pandemic outbreak in Italy. A Systematic review. Mediterranean Journal of Clinical Psychology. 2021;9(2).

50. Caponnetto P, Benenati A, Maglia MG. Psychopathological Impact and Resilient Scenarios in Inpatient with Schizophrenia Spectrum Disorders Related to Covid Physical Distancing Policies: A Systematic Review. Behavioral sciences. 2021;11(4):13.

51. Carbone EA, de Filippis R, Roberti R, Rania M, Destefano L, Russo E, et al. The Mental Health of Caregivers and Their Patients With Dementia During the COVID-19 Pandemic: A Systematic Review. Frontiers in Psychology. 2021;12.

52. Carmassi C, Foghi C, Dell'Oste V, Cordone A, Bertelloni CA, Bui E, et al. PTSD symptoms in healthcare workers facing the three coronavirus outbreaks: What can we expect after the COVID-19 pandemic. Psychiatry Research. 2020;292:113312.

53. Caruso R, Annaloro C, Arrigoni C, Ghizzardi G, Dellafiore F, Magon A, et al. Burnout and post-traumatic stress disorder in frontline nurses during the COVID-19 pandemic: a systematic literature review and meta-analysis of studies published in 2020: COVID-19, burnout, and PTSD in nurses. Acta Biomedica Atenei Parmensis. 2021;92(S2):e2021428.

54. Cavicchioli M, Ferrucci R, Guidetti M, Canevini MP, Pravettoni G, Galli F. What Will Be the Impact of the Covid-19 Quarantine on Psychological Distress? Considerations Based on a Systematic Review of Pandemic Outbreaks. Healthcare. 2021;9(1):19.

55. Cenat JM, Blais-Rochette C, Kokou-Kpolou CK, Noorishad PG, Mukunzi JN, McIntee SE, et al. Prevalence of symptoms of depression, anxiety, insomnia, posttraumatic stress disorder, and psychological distress among populations affected by the COVID-19 pandemic: A systematic review and meta-analysis. Psychiatry Research. 2021;295:113599.

56. Chaabane S, Doraiswamy S, Chaabna K, Mamtani R, Cheema S. The Impact of COVID-19 School Closure on Child and Adolescent Health: A Rapid Systematic Review. Children. 2021;8(5):415.

57. Chai J, Xu H, An N, Zhang P, Liu F, He S, et al. The Prevalence of Mental Problems for Chinese Children and Adolescents During COVID-19 in China: A Systematic Review and Meta-Analysis. Frontiers in Pediatrics. 2021;9.

58. Chang J-J, Ji Y, Li Y-H, Pan H-F, Su P-Y. Prevalence of anxiety symptom and depressive symptom among college students during COVID-19 pandemic: A meta-analysis. Journal of Affective Disorders. 2021;292:242-54.

59. Chawla N, Tom A, Sen MS, Sagar R. Psychological Impact of COVID-19 on Children and Adolescents: A Systematic Review. Indian Journal of Psychological Medicine. 2021;43(4):294-9.

60. Chekole YA, Abate SM. Global prevalence and determinants of mental health disorders during the COVID-19 pandemic: A systematic review and meta-analysis. Annals of Medicine and Surgery. 2021;68:102634.

61. Chen J, Farah N, Dong RK, Chen RZ, Xu W, Yin J, et al. Mental Health during the COVID-19 Crisis in Africa: A Systematic Review and Meta-Analysis. International Journal of Environmental Research and Public Health. 2021;18(20):10604.

62. Cheng CKT, Chua JH, Cheng LJ, Ang WHD, Lau Y. Global prevalence of resilience in health care professionals: A systematic review, meta-analysis and meta-regression. Journal of Nursing Management. 2022;30(3):795-816.

63. Chigwedere OC, Sadath A, Kabir Z, Arensman E. The Impact of Epidemics and Pandemics on the Mental Health of Healthcare Workers: A Systematic Review. International Journal of Environmental Research and Public Health. 2021;18(13):6695.

64. Ching SM, Ng KY, Lee KW, Yee A, Lim PY, Ranita H, et al. Psychological distress among healthcare providers during COVID-19 in Asia: Systematic review and meta-analysis. PLOS ONE. 2021;16(10):e0257983.

65. Chirico F, Zaffina S, Di Prinzio RR, Giorgi G, Ferrari G, Capitanelli I, et al. Working from home in the context of COVID-19: A systematic review of physical and mental health effects on teleworkers. Journal of Health and Social Sciences. 2021;6:319-32.

66. Chtourou H, Trabelsi K, H'Mida C, Boukhris O, Glenn JM, Brach M, et al. Staying Physically Active During the Quarantine and Self-Isolation Period for Controlling and Mitigating the COVID-19 Pandemic: A Systematic Overview of the Literature. Frontiers in Psychology. 2020;11:1708.

67. Chu IY, Alam P, Larson HJ, Lin L. Social consequences of mass quarantine during epidemics: a systematic review with implications for the COVID-19 response. Journal of Travel Medicine. 2020;27(7):09.

68. Ciuffreda G, Cabanillas-Barea S, Carrasco-Uribarren A, Albarova-Corral MI, Argüello-Espinosa MI, Marcén-Román Y. Factors Associated with Depression and Anxiety in Adults ≥60 Years Old during the COVID-19 Pandemic: A Systematic Review. International Journal of Environmental Research and Public Health. 2021;18(22):11859.

69. Crocamo C, Bachi B, Calabrese A, Callovini T, Cavaleri D, Cioni RM, et al. Some of us are most at risk: Systematic review and meta-analysis of correlates of depressive symptoms among healthcare workers during the SARS-CoV-2 outbreak. Neuroscience & Biobehavioral Reviews. 2021;131:912-22.

70. Cunning C, Hodes M. The COVID-19 pandemic and obsessive–compulsive disorder in young people: Systematic review. Clinical Child Psychology and Psychiatry. 2021;27(1):18-34.

71. d'Ettorre G, Ceccarelli G, Santinelli L, Vassalini P, Innocenti GP, Alessandri F, et al. Post-Traumatic Stress Symptoms in Healthcare Workers Dealing with the COVID-19 Pandemic: A Systematic Review. International Journal of Environmental Research & Public Health [Electronic Resource]. 2021;18(2):12.

72. D'Ettorre G, Pellicani V, Ceccarelli G. Post-traumatic stress disorder symptoms in healthcare workers: A ten-year systematic review. Acta Biomedica. 2020;91(12-S):1-10.

73. da Silva FCT, Barbosa CP. The impact of the COVID-19 pandemic in an intensive care unit (ICU): Psychiatric symptoms in healthcare professionals. Progress in Neuro-Psychopharmacology & Biological Psychiatry. 2021;110:110299.

74. da Silva FCT, Neto MLR. Psychiatric symptomatology associated with depression, anxiety, distress, and insomnia in health professionals working in patients affected by COVID-19: A systematic review with meta-analysis. Progress in Neuro-Psychopharmacology and Biological Psychiatry. 2021;104.

75. da Silva ML, Rocha RSB, Buheji M, Jahrami H, Cunha KDC. A systematic review of the prevalence of anxiety symptoms during coronavirus epidemics. Journal of Health Psychology. 2021;26(1):115-25.

76. De Brier N, Stroobants S, Vandekerckhove P, De Buck E. Factors affecting mental health of health care workers during coronavirus disease outbreaks (SARS, MERS & COVID-19): A rapid systematic review. PLoS ONE. 2020;15(12 December).

77. Della Monica A, Ferrara P, Dal Mas F, Cobianchi L, Scannapieco F, FR FR. The impact of Covid-19 healthcare emergency on the psychological well-being of health professionals: a review of literature. Annali di Igiene. 2021;30:30.

78. Dellazizzo L, Léveillé N, Landry C, Dumais A. Systematic Review on the Mental Health and Treatment Impacts of COVID-19 on Neurocognitive Disorders. Journal of Personalized Medicine. 2021;11(8):746.

79. Demissie DB, Bitew ZW. Mental health effect of COVID-19 pandemic among women who are pregnant and/or lactating: A systematic review and meta-analysis. SAGE Open Medicine. 2021;9:20503121211026195.

80. Deng J, Zhou F, Hou W, Silver Z, Wong CY, Chang O, et al. The prevalence of depressive symptoms, anxiety symptoms and sleep disturbance in higher education students during the COVID-19 pandemic: A systematic review and meta-analysis. Psychiatry Research. 2021;301:113863.

81. Dettmann LM, Adams S, Taylor G. Investigating the prevalence of anxiety and depression during the first COVID-19 lockdown in the United Kingdom: Systematic review and meta-analyses. British Journal of Clinical Psychology. 2022;61(3):757-80.

82. Dhada S, Stewart D, Cheema E, Hadi MA, Paudyal V. Cancer services during the COVID-19 pandemic: Systematic review of patient’s and caregiver’s experiences. Cancer management and research. 2021;13:5875.

83. Dong F, Liu H-l, Yang M, Lu C-l, Dai N, Zhang Y, et al. Immediate Psychosocial Impact on Healthcare Workers During COVID-19 Pandemic in China: A Systematic Review and Meta-Analysis. Frontiers in Psychology. 2021;12.

84. Dong F, Liu H-l, Dai N, Yang M, Liu J-p. A living systematic review of the psychological problems in people suffering from COVID-19. Journal of Affective Disorders. 2021;292:172-88.

85. Dorri M, Mozafari Bazargany MH, Khodaparast Z, Bahrami S, Seifi Alan M, Rahimi F, et al. Psychological problems and reduced health-related quality of life in the COVID-19 survivors. Journal of Affective Disorders Reports. 2021;6:100248.

86. Dos Santos ERR, de Paula JLS, Tardieux FM, Costa-e-Silva VN, Lal A, Leite AFB. Association between COVID-19 and anxiety during social isolation: A systematic review. World Journal of Clinical Cases. 2021;9(25):7433.

87. Dragioti E, Li H, Tsitsas G, Lee KH, Choi J, Kim J, et al. A large-scale meta-analytic atlas of mental health problems prevalence during the COVID-19 early pandemic. Journal of Medical Virology. 2022;94(5):1935-49.

88. Dubé JP, Smith MM, Sherry SB, Hewitt PL, Stewart SH. Suicide behaviors during the COVID-19 pandemic: A meta-analysis of 54 studies. Psychiatry Research. 2021;301:113998.

89. Dullius WR, Scortegagna SA, McCleary L. Coping strategies in health professionals facing Covid-19: s systematic review. Psicologia: teoria e prática. 2021;23(1):1-20.

90. Dutta A, Sharma A, Torres-Castro R, Pachori H, Mishra S. Mental health outcomes among health-care workers dealing with COVID-19/severe acute respiratory syndrome coronavirus 2 pandemic: A systematic review and meta-analysis. Indian Journal of Psychiatry. 2021;63(4).

91. Elharake JA, Akbar F, Malik AA, Gilliam W, Omer SB. Mental Health Impact of COVID-19 among Children and College Students: A Systematic Review. Child Psychiatry & Human Development. 2022.

92. Esposito C, Di Napoli I, Agueli B, Marino L, Procentese F, Arcidiacono C. Well-being and the COVID-19 pandemic: A community psychology systematic review. European Psychologist. 2021;26(4):285.

93. Fan FC, Zhang SY, Cheng Y. Incidence of psychological illness after coronavirus outbreak: a meta-analysis study. Journal of Epidemiology & Community Health. 2021;25:25.

94. Fan S, Guan J, Cao L, Wang M, Zhao H, Chen L, et al. Psychological effects caused by COVID-19 pandemic on pregnant women: A systematic review with meta-analysis. Asian Journal of Psychiatry. 2020;56:102533.

95. Farooq S, Tunmore J, Wajid Ali M, Ayub M. Suicide, self-harm and suicidal ideation during COVID-19: A systematic review. Psychiatry Research. 2021;306:114228.

96. Fleischmann E, Dalkner N, Fellendorf FT, Reininghaus EZ. Psychological impact of the COVID-19 pandemic on individuals with serious mental disorders: A systematic review of the literature. World Journal of Psychiatry. 2021;11(12):1387.

97. d'Ettorre G, Gentilini Cacciola E, Santinelli L, De Girolamo G, Spagnolello O, Russo A, et al. Covid-19 sequelae in working age patients: A systematic review. Journal of Medical Virology. 2022;94(3):858-68.

98. Gentry SV, Thomas-Meyer M, Tyrrell CSB, Mavrodaris A, Williams R, Wallbank S, et al. What are the mental health impacts of epidemics on relatives of people affected, and relatives of healthcare workers: What interventions are available to support them? A systematic review and narrative synthesis. Comprehensive Psychiatry. 2022;113:152288.

99. Ghazanfarpour M, Bahrami F, Rashidi Fakari F, Ashrafinia F, Babakhanian M, Dordeh M, et al. Prevalence of anxiety and depression among pregnant women during the COVID-19 pandemic: a meta-analysis. Journal of Psychosomatic Obstetrics & Gynecology. 2021:1-12.

100. Gianfredi V, Provenzano S, Santangelo OE. What can internet users' behaviours reveal about the mental health impacts of the COVID-19 pandemic? A systematic review. Public Health. 2021;198:44-52.

101. Gibson B, Schneider J, Talamonti D, Forshaw M. The impact of inequality on mental health outcomes during the COVID-19 pandemic: A systematic review. Canadian Psychology/Psychologie canadienne. 2021;62(1):101-26.

102. Gray KL, Birtles H, Reichelt K, James IA. The experiences of care home staff during the COVID-19 pandemic: A systematic review. Aging & Mental Health. 2022;26(10):2080-9.

103. Groff D, Sun A, Ssentongo AE, Ba DM, Parsons N, Poudel GR, et al. Short-term and Long-term Rates of Postacute Sequelae of SARS-CoV-2 Infection: A Systematic Review. JAMA Network Open. 2021;4(10):e2128568-e.

104. Gross JV, Mohren J, Erren TC. COVID-19 and healthcare workers: a rapid systematic review into risks and preventive measures. BMJ Open. 2021;11(1):e042270.

105. Guo S, Kaminga AC, Xiong J. Depression and Coping Styles of College Students in China During COVID-19 Pandemic: A Systemic Review and Meta-Analysis. Front Public Health. 2021;9.

106. Guzick AG, Candelari A, Wiese AD, Schneider SC, Goodman WK, Storch EA. Obsessive–Compulsive Disorder During the COVID-19 Pandemic: a Systematic Review. Current Psychiatry Reports. 2021;23(11):71.

107. Han Q, Zheng B, Daines L, Sheikh A. Long-Term Sequelae of COVID-19: A Systematic Review and Meta-Analysis of One-Year Follow-Up Studies on Post-COVID Symptoms. Pathogens. 2022;11(2):269.

108. Hannemann J, Abdalrahman A, Erim Y, Morawa E, Jerg-Bretzke L, Beschoner P, et al. The impact of the COVID-19 pandemic on the mental health of medical staff considering the interplay of pandemic burden and psychosocial resources—A rapid systematic review. PLOS ONE. 2022;17(2):e0264290.

109. Hao Q, Wang D, Xie M, Tang Y, Dou Y, Zhu L, et al. Prevalence and Risk Factors of Mental Health Problems Among Healthcare Workers During the COVID-19 Pandemic: A Systematic Review and Meta-Analysis. Frontiers in Psychiatry. 2021;12.

110. HEKMAT A, DIVANBEIGI R, YEGANE DSAF. Effects of COVID-19 on Student’s mental health: a systematic review. Pakistan Journal of Medical and Health Sciences. 2021:1543-50.

111. Hesary FB, Salehiniya H. The Impact of the COVID-19 Epidemic on Diagnosis, Treatment, Concerns, Problems, and Mental Health in Patients with Gastric Cancer. Journal of Gastrointestinal Cancer. 2022;53(3):797-804.

112. Hessami K, Romanelli C, Chiurazzi M, Cozzolino M. COVID-19 pandemic and maternal mental health: a systematic review and meta-analysis. Journal of Maternal-Fetal & Neonatal Medicine. 2020:1-8.

113. Hill JE, Harris C, Danielle L. C, Boland P, Doherty Alison J, Benedetto V, et al. The prevalence of mental health conditions in healthcare workers during and after a pandemic: Systematic review and meta-analysis. Journal of Advanced Nursing. 2022;78(6):1551-73.

114. Hintermeier M, Gencer H, Kajikhina K, Rohleder S, Hövener C, Tallarek M, et al. SARS-CoV-2 among migrants and forcibly displaced populations: A rapid systematic review. Journal of Migration and Health. 2021;4:100056.

115. Hosen I, al-Mamun F, Mamun MA. Prevalence and risk factors of the symptoms of depression, anxiety, and stress during the COVID-19 pandemic in Bangladesh: a systematic review and meta-analysis. Global Mental Health. 2021;8:e47.

116. Hossain MM, Rahman M, Trisha NF, Tasnim S, Nuzhath T, Hasan NT, et al. Prevalence of anxiety and depression in South Asia during COVID-19: A systematic review and meta-analysis. Heliyon. 2021;7(4):e06677.

117. Hu N, Deng H, Yang H, Wang C, Cui Y, Chen J, et al. The pooled prevalence of the mental problems of Chinese medical staff during the COVID-19 outbreak: A meta-analysis. Journal of Affective Disorders. 2022;303:323-30.

118. Huerta-González S, Selva-Medrano D, López-Espuela F, Caro-Alonso PÁ, Novo A, Rodríguez-Martín B. The Psychological Impact of COVID-19 on Front Line Nurses: A Synthesis of Qualitative Evidence. International Journal of Environmental Research and Public Health. 2021;18(24):12975.

119. Hugelius K, Harada N, Marutani M. Consequences of visiting restrictions during the COVID‐19 pandemic: An integrative review. International Journal of Nursing Studies. 2021;121:104000.

120. Imran N, Aamer I, Sharif MI, Bodla ZH, Naveed S. Psychological burden of quarantine in children and adolescents: A rapid systematic review and proposed solutions. Pakistan Journal of Medical Sciences. 2020;36(5):1106-16.

121. Imtiaz S, Nafeh F, Russell C, Ali F, Elton-Marshall T, Rehm J. The impact of the novel coronavirus disease (COVID-19) pandemic on drug overdose-related deaths in the United States and Canada: a systematic review of observational studies and analysis of public health surveillance data. Substance Abuse Treatment, Prevention, and Policy. 2021;16(1):87.

122. Iyengar U, Jaiprakash B, Haitsuka H, Kim S. One Year Into the Pandemic: A Systematic Review of Perinatal Mental Health Outcomes During COVID-19. Frontiers in Psychiatry. 2021;12.

123. Jammu AS, Chasen MR, Lofters AK, Bhargava R. Systematic rapid living review of the impact of the COVID-19 pandemic on cancer survivors: update to August 27, 2020. Supportive Care in Cancer. 2021;29(6):2841-50.

124. Jennings G, Monaghan A, Xue F, Mockler D, Romero-Ortuño R. A Systematic Review of Persistent Symptoms and Residual Abnormal Functioning following Acute COVID-19: Ongoing Symptomatic Phase vs. Post-COVID-19 Syndrome. Journal of Clinical Medicine. 2021;10(24):5913.

125. Jesline J, Romate J, Rajkumar E, George AJ. The plight of migrants during COVID-19 and the impact of circular migration in India: a systematic review. Humanities and Social Sciences Communications. 2021;8(1):231.

126. Johns G, Samuel V, Freemantle L, Lewis J, Waddington L. The global prevalence of depression and anxiety among doctors during the covid-19 pandemic: Systematic review and meta-analysis. Journal of Affective Disorders. 2022;298:431-41.

127. Jones EAK, Mitra AK, Bhuiyan AR. Impact of COVID-19 on Mental Health in Adolescents: A Systematic Review. International Journal of Environmental Research & Public Health [Electronic Resource]. 2021;18(5):03.

128. Jothishanmugam A, Begum S, Abdelgader AAM, Allahamid HIA, Abdalghani NAA. Psychological impact of covid-19 outbreak on nurses: A systematic review. International Journal of Research in Pharmaceutical Sciences. 2020;11(Special Issue 4):1905-13.

129. Jurecka A, Skucińska P, Gądek A. Impact of the SARS-CoV-2 Coronavirus Pandemic on Physical Activity, Mental Health and Quality of Life in Professional Athletes—A Systematic Review. International Journal of Environmental Research and Public Health. 2021;18(17):9423.

130. Kahil K, Cheaito MA, El Hayek R, Nofal M, El Halabi S, Kudva KG, et al. Suicide during COVID-19 and other major international respiratory outbreaks: A systematic review. Asian Journal of Psychiatry. 2020;56:102509.

131. Keskin S, Özkan B. Mental statuses of nursing students in the covid-19 pandemic period: a systematic review. Annals of Clinical and Analytical Medicine. 2021:245-9.

132. Khaffaf ES, Madalah ZT. The Relation between Mental Health and Covid-19 Pandemic: A Systematic Review Study. Pakistan Journal of Medical and Health Sciences. 2021;15(4):1155-7.

133. Khoundabi B, Soltani A, Marzaleh MA. Psychological Problems of the Nursing Staff in COVID-19 Pandemic: A Systematic Review. Iranian Red Crescent Medical Journal. 2021;23(6).

134. Khraisat BR, Al-Jeady AM, Alqatawneh DA, Toubasi AA, AlRyalat SA. The prevalence of mental health outcomes among eating disorder patients during the COVID-19 pandemic: A meta-analysis. Clinical Nutrition ESPEN. 2022;48:141-7.

135. Khraisat B, Toubasi A, AlZoubi L, Al-Sayegh T, Mansour A. Meta-analysis of prevalence: the psychological sequelae among COVID-19 survivors. International Journal of Psychiatry in Clinical Practice. 2021:1-10.

136. Killikelly C, Lenferink LIM, Xie H, Maercker A. Rapid Systematic Review of Psychological Symptoms in Health Care Workers COVID-19. Journal of Loss and Trauma. 2021.

137. Kirubarajan A, Patel P, Tsang J, Prethipan T, Sreeram P, Sierra S. The psychological impact of the COVID-19 pandemic on fertility care: a qualitative systematic review. Human Fertility. 2021:1-8.

138. Kisely S, Warren N, McMahon L, Dalais C, Henry I, Siskind D. Occurrence, prevention, and management of the psychological effects of emerging virus outbreaks on healthcare workers: rapid review and meta-analysis. BMJ. 2020;369:m1642.

139. Koontalay A, Suksatan W, Prabsangob K, Sadang JM. Healthcare workers’ burdens during the COVID-19 pandemic: A qualitative systematic review. Journal of Multidisciplinary Healthcare. 2021;14:3015.

140. Krishnamoorthy Y, Nagarajan R, Saya GK, Menon V. Prevalence of psychological morbidities among general population, healthcare workers and COVID-19 patients amidst the COVID-19 pandemic: A systematic review and meta-analysis. Psychiatry Research. 2020;293.

141. Kunz M, Strasser M, Hasan A. Impact of the coronavirus disease 2019 pandemic on healthcare workers: systematic comparison between nurses and medical doctors. Current Opinion in Psychiatry. 2021;34(4).

142. Kunzler AM, Rothke N, Gunthner L, Stoffers-Winterling J, Tuscher O, Coenen M, et al. Mental burden and its risk and protective factors during the early phase of the SARS-CoV-2 pandemic: systematic review and meta-analyses. Global Health. 2021;17(1):34.

143. Kuroda N, Kubota T. Psychological impact of the COVID-19 pandemic for patients with epilepsy: A systematic review and meta-analysis. Epilepsy & Behavior. 2021;124.

144. Kusumawati MW, Soeharto S, Windarwati HD. Psychosocial Impact and Protective Factor of COVID-19 Confirmed Patient During Isolation Enactment: A Systematic Review. Indian Journal of Forensic Medicine & Toxicology. 2022;16(1).

145. Lasheras I, Gracia-Garcia P, Lipnicki DM, Bueno-Notivol J, Lopez-Anton R, de la Camara C, et al. Prevalence of Anxiety in Medical Students during the COVID-19 Pandemic: A Rapid Systematic Review with Meta-Analysis. International Journal of Environmental Research & Public Health [Electronic Resource]. 2020;17(18):10.

146. Lee H-J, Park B-M. Feelings of Entrapment during the COVID-19 Pandemic Based on ACE Star Model: A Concept Analysis. Healthcare. 2021;9(10):1305.

147. Lee KW, Ang CS, Lim SH, Siau CS, Ong Lai Teik D, Ching SM, et al. Prevalence of mental health conditions among people living with HIV during the COVID-19 pandemic: A rapid systematic review and meta-analysis. HIV Medicine. 2022;23(9):990-1001.

148. Lehmann J, Lechner V, Scheithauer H. School Closures During the COVID-19 Pandemic: Psychosocial Outcomes in Children - a Systematic Review. International Journal of Developmental Science. 2021;15:85-111.

149. Li W, Zhang H, Zhang C, Luo J, Wang H, Wu H, et al. The Prevalence of Psychological Status During the COVID-19 Epidemic in China: A Systemic Review and Meta-Analysis. Frontiers in Psychology. 2021;12.

150. Li W, Zhao Z, Chen D, Peng Y, Lu Z. Prevalence and associated factors of depression and anxiety symptoms among college students: a systematic review and meta-analysis. Journal of Child Psychology and Psychiatry.n/a(n/a).

151. Li Y, Scherer N, Felix L, Kuper H. Prevalence of depression, anxiety and post-traumatic stress disorder in health care workers during the COVID-19 pandemic: A systematic review and meta-analysis. PLoS ONE [Electronic Resource]. 2021;16(3):e0246454.

152. Lieneck C, Bosworth M, Weaver E, Heinemann K, Patel J. Protective and Non-Protective Factors of Mental Health Distress in the United States during the COVID-19 Pandemic: A Systematic Review. Medicina. 2021;57(12):1377.

153. Lin CT, Ming LW, Kyi TK. Contemporary Issues in Well-being of Undergraduate Clinical Students: A Systematic Review. Education in Medicine Journal. 2021;13(1).

154. Lin CY, Lin YL. Anxiety and depression of general population in the early phase of COVID-19 pandemic: A systematic review of cross-sectional studies. Revista de Psiquiatria Clinica. 2020;47(6):199-208.

155. Linardon J, Messer M, Rodgers RF, Fuller-Tyszkiewicz M. A systematic scoping review of research on COVID-19 impacts on eating disorders: A critical appraisal of the evidence and recommendations for the field. International Journal of Eating Disorders. 2022;55(1):3-38.

156. Liyanage S, Saqib K, Khan AF, Thobani TR, Tang W-C, Chiarot CB, et al. Prevalence of Anxiety in University Students during the COVID-19 Pandemic: A Systematic Review. International Journal of Environmental Research and Public Health. 2022;19(1):62.

157. Luo F, Ghanei Gheshlagh R, Dalvand S, Saedmoucheshi S, Li Q. Systematic Review and Meta-Analysis of Fear of COVID-19. Frontiers in Psychology. 2021;12.

158. Luo M, Guo L, Yu M, Wang H. The psychological and mental impact of coronavirus disease 2019 (COVID-19) on medical staff and general public - A systematic review and meta-analysis. Psychiatry Research. 2020;291 (no pagination).

159. Mamun MA. Suicide and suicidal behaviors in the context of COVID-19 pandemic in Bangladesh: a systematic review. Psychology Research and Behavior Management. 2021;14:695.

160. Luo W, Zhong BL, Chiu HF. Prevalence of depressive symptoms among Chinese university students amid the COVID-19 pandemic: a systematic review and meta-analysis. Epidemiology & Psychiatric Science. 2021;30:e31.

161. Luo Y, Chua CR, Xiong Z, Ho RC, Ho CSH. A Systematic Review of the Impact of Viral Respiratory Epidemics on Mental Health: An Implication on the Coronavirus Disease 2019 Pandemic. Frontiers in Psychiatry. 2020;11.

162. Luo Y, Zhang K, Huang M, Qiu C. Risk factors for depression and anxiety in pregnant women during the COVID-19 pandemic: Evidence from meta-analysis. PLOS ONE. 2022;17(3):e0265021.

163. Ma L, Mazidi M, Li K, Li Y, Chen S, Kirwan R, et al. Prevalence of mental health problems among children and adolescents during the COVID-19 pandemic: A systematic review and meta-analysis. Journal of Affective Disorders. 2021;293:78-89.

164. Mahmud S, Hossain S, Muyeed A, Islam MM, Mohsin M. The global prevalence of depression, anxiety, stress, and, insomnia and its changes among health professionals during COVID-19 pandemic: A rapid systematic review and meta-analysis. Heliyon. 2021;7(7).

165. Mahmud S, Mohsin M, Dewan MN, Muyeed A. The Global Prevalence of Depression, Anxiety, Stress, and Insomnia Among General Population During COVID-19 Pandemic: A Systematic Review and Meta-analysis. Trends in Psychology. 2022.

166. Malik P, Patel K, Pinto C, Jaiswal R, Tirupathi R, Pillai S, et al. Post-acute COVID-19 syndrome (PCS) and health-related quality of life (HRQoL)—A systematic review and meta-analysis. Journal of Medical Virology. 2022;94(1):253-62.

167. Marciano L, Ostroumova M, Schulz PJ, Camerini A-L. Digital Media Use and Adolescents' Mental Health During the Covid-19 Pandemic: A Systematic Review and Meta-Analysis. Front Public Health. 2022;9.

168. Marconcin P, Werneck AO, Peralta M, Ihle A, Gouveia ÉR, Ferrari G, et al. The association between physical activity and mental health during the first year of the COVID-19 pandemic: a systematic review. BMC Public Health. 2022;22(1):209.

169. Marvaldi M, Mallet J, Dubertret C, Moro MR, Guessoum SB. Anxiety, depression, trauma-related, and sleep disorders among healthcare workers during the COVID-19 pandemic: A systematic review and meta-analysis. Neuroscience & Biobehavioral Reviews. 2021;126:252-64.

170. Matondang ERS, Suza DE, Tarigan AP. Nurse’s Experience In Caring For COVID-19 Patients: A Systematic Review. Malaysian Journal of Medicine and Health Sciences. 2021(SUPP4):137-42.

171. McGowan VJ, Lowther HJ, Meads C. Life under COVID-19 for LGBT+ people in the UK: systematic review of UK research on the impact of COVID-19 on sexual and gender minority populations. BMJ Open. 2021;11(7):e050092.

172. Meherali S, Punjani N, Louie-Poon S, Abdul Rahim K, Das JK, Salam RA, et al. Mental Health of Children and Adolescents Amidst COVID-19 and Past Pandemics: A Rapid Systematic Review. International Journal of Environmental Research & Public Health [Electronic Resource]. 2021;18(7):26.

173. Min S, Jeong YH, Kim J, Koo JW, Ahn YM. The Aftermath: Post-pandemic Psychiatric Implications of the COVID-19 Pandemic, a South Korean Perspective. Frontiers in Psychiatry. 2021;12.

174. Miniati M, Marzetti F, Palagini L, Marazziti D, Orrù G, Conversano C, et al. Eating Disorders Spectrum During the COVID Pandemic: A Systematic Review. Frontiers in Psychology. 2021;12.

175. Mohamed N, Abidin E, Rasdi I, Ismail Z, Ismail N. Occupational Stressors Experienced by Police Officers during Coronavirus Disease (Covid-19) Outbreak–A Systematic Literature Review. Malaysian Journal of Medicine and Health Sciences. 2021:167-76.

176. Momenimovahed Z, Salehiniya H, Hadavandsiri F, Allahqoli L, Günther V, Alkatout I. Psychological Distress Among Cancer Patients During COVID-19 Pandemic in the World: A Systematic Review. Frontiers in Psychology. 2021;12.

177. Monteleone AM, Cascino G, Barone E, Carfagno M, Monteleone P. COVID-19 Pandemic and Eating Disorders: What Can We Learn About Psychopathology and Treatment? A Systematic Review. Current Psychiatry Reports. 2021;23(12):83.

178. Morrish N, Medina-Lara A. Does unemployment lead to greater levels of loneliness? A systematic review. Soc Sci Med. 2021;287:114339.

179. Mousavizadeh SN, Merdasi PG, Safari M. Psychological challenges of nurses in pandemic Covid-19. Pakistan Journal of Medical and Health Sciences. 2021;15(1):448-55.

180. Muller AE, Hafstad EV, Himmels JPW, Smedslund G, Flottorp S, Stensland SØ, et al. The mental health impact of the covid-19 pandemic on healthcare workers, and interventions to help them: A rapid systematic review. Psychiatry Research. 2020;293.

181. Mulyadi M, Tonapa SI, Luneto S, Lin W-T, Lee B-O. Prevalence of mental health problems and sleep disturbances in nursing students during the COVID-19 pandemic: A systematic review and meta-analysis. Nurse Education in Practice. 2021;57:103228.

182. Munro A, Booth H, Gray NM, Love J, Mohan ARM, Tang J, et al. Understanding the Impacts of Novel Coronavirus Outbreaks on People Who Use Drugs: A Systematic Review to Inform Practice and Drug Policy Responses to COVID-19. International Journal of Environmental Research and Public Health. 2021;18(16):8470.

183. Nagarajan R, Krishnamoorthy Y, Basavarachar V, Dakshinamoorthy R. Prevalence of post-traumatic stress disorder among survivors of severe COVID-19 infections: A systematic review and meta-analysis. Journal of Affective Disorders. 2022;299:52-9.

184. Nagi R, Reddy SS, Rakesh N, Vyas T. Tobacco cessation is a challenge during COVID-19 pandemic: Is it a good time to quit?: A systematic review. Journal of Indian Academy of Oral Medicine and Radiology. 2021;33(1):82.

185. Nam S-H, Nam J-H, Kwon C-Y. Comparison of the Mental Health Impact of COVID-19 on Vulnerable and Non-Vulnerable Groups: A Systematic Review and Meta-Analysis of Observational Studies. International Journal of Environmental Research and Public Health. 2021;18(20):10830.

186. Nasserie T, Hittle M, Goodman SN. Assessment of the Frequency and Variety of Persistent Symptoms Among Patients With COVID-19: A Systematic Review. JAMA Network Open. 2021;4(5):e2111417-e.

187. Nearchou F, Flinn C, Niland R, Subramaniam SS, Hennessy E. Exploring the Impact of COVID-19 on Mental Health Outcomes in Children and Adolescents: A Systematic Review. International Journal of Environmental Research & Public Health [Electronic Resource]. 2020;17(22):16.

188. Necho M, Tsehay M, Birkie M, Biset G, Tadesse E. Prevalence of anxiety, depression, and psychological distress among the general population during the COVID-19 pandemic: A systematic review and meta-analysis. International Journal of Social Psychiatry. 2021:207640211003121.

189. Neelam K, Duddu V, Anyim N, Neelam J, Lewis S. Pandemics and pre-existing mental illness: A systematic review and meta-analysis. Brain, Behavior, and Immunity - Health. 2021;10 (no pagination).

190. Nobari H, Fashi M, Eskandari A, Villafaina S, Murillo-Garcia A, Perez-Gomez J. Effect of COVID-19 on Health-Related Quality of Life in Adolescents and Children: A Systematic Review. International Journal of Environmental Research & Public Health [Electronic Resource]. 2021;18(9):25.

191. Norhayati MN, Che Yusof R, Azman MY. Prevalence of Psychological Impacts on Healthcare Providers during COVID-19 Pandemic in Asia. International Journal of Environmental Research and Public Health. 2021;18(17):9157.

192. Nowrouzi-Kia B, Sithamparanathan G, Nadesar N, Gohar B, Ott M. Factors associated with work performance and mental health of healthcare workers during pandemics: a systematic review and meta-analysis. Journal of Public Health. 2021.

193. Nursalam N, Sukartini T, Priyantini D, Mafula D, Efendi F. Risk factors for psychological impact and social stigma among people facing COVID 19: A systematic review. Systematic Reviews in Pharmacy. 2020;11(6):1022-8.

194. Okpua NC, Edeogu OC, Hami R, Mujar NMM. Impacts of Covid-19 Pandemic on Frontline Health Care Workers in Africa and Asian Countries: A Systematic Review. Malaysian Journal of Medicine & Health Sciences. 2021:118-30.

195. Olashore A, Akanni O, Fela-Thomas A, Khutsafalo K. The psychological impact of COVID-19 on health-care workers in African Countries: A systematic review. Asian Journal of Social Health and Behavior. 2021;4(3):85-97.

196. Olaya B, Pérez-Moreno M, Bueno-Notivol J, Gracia-García P, Lasheras I, Santabárbara J. Prevalence of Depression among Healthcare Workers during the COVID-19 Outbreak: A Systematic Review and Meta-Analysis. Journal of Clinical Medicine. 2021;10(15):3406.

197. Oliveira Carvalho P, Hülsdünker T, Carson F. The Impact of the COVID-19 Lockdown on European Students&rsquo; Negative Emotional Symptoms: A Systematic Review and Meta-Analysis. Behavioral Sciences. 2022;12(1):3.

198. Oliveira JMDd, Butini L, Pauletto P, Lehmkuhl KM, Stefani CM, Bolan M, et al. Mental health effects prevalence in children and adolescents during the COVID-19 pandemic: A systematic review. Worldviews on Evidence-Based Nursing. 2022;19(2):130-7.

199. Ozamiz-Etxebarria N, Idoiaga Mondragon N, Bueno-Notivol J, Pérez-Moreno M, Santabárbara J. Prevalence of Anxiety, Depression, and Stress among Teachers during the COVID-19 Pandemic: A Rapid Systematic Review with Meta-Analysis. Brain Sciences. 2021;11(9):1172.

200. Özgüç S, Kaplan Serin E, Tanriverdi D. Death Anxiety Associated With Coronavirus (COVID-19) Disease: A Systematic Review and Meta-Analysis. OMEGA - Journal of Death and Dying. 2021:00302228211050503.

201. Pai N, Vella S-L. COVID-19 and loneliness: A rapid systematic review. Australian & New Zealand Journal of Psychiatry. 2021;55(12):1144-56.

202. Panchal U, Salazar de Pablo G, Franco M, Moreno C, Parellada M, Arango C, et al. The impact of COVID-19 lockdown on child and adolescent mental health: systematic review. European Child & Adolescent Psychiatry. 2021.

203. Panda PK, Gupta J, Chowdhury SR, Kumar R, Meena AK, Madaan P, et al. Psychological and Behavioral Impact of Lockdown and Quarantine Measures for COVID-19 Pandemic on Children, Adolescents and Caregivers: A Systematic Review and Meta-Analysis. Journal of tropical pediatrics. 2020;27.

204. Pappa S, Ntella V, Giannakas T, Giannakoulis VG, Papoutsi E, Katsaounou P. Prevalence of depression, anxiety, and insomnia among healthcare workers during the COVID-19 pandemic: A systematic review and meta-analysis. Brain, Behavior, and Immunity. 2020;92:247.

205. Pappa S, Chen J, Barnett J, Chang A, Dong RK, Xu W, et al. A systematic review and meta-analysis of the mental health symptoms during the Covid-19 pandemic in Southeast Asia. Psychiatry and Clinical Neurosciences. 2022;76(2):41-50.

206. Pashazadeh Kan F, Raoofi S, Rafiei S, Khani S, Hosseinifard H, Tajik F, et al. A systematic review of the prevalence of anxiety among the general population during the COVID-19 pandemic. Journal of Affective Disorders. 2021;293:391-8.

207. Phiri P, Ramakrishnan R, Rathod S, Elliot K, Thayanandan T, Sandle N, et al. An evaluation of the mental health impact of SARS-CoV-2 on patients, general public and healthcare professionals: A systematic review and meta-analysis. EClinicalMedicine. 2021;34:100806.

208. Pian W, Chi J, Ma F. The causes, impacts and countermeasures of COVID-19 “Infodemic”: A systematic review using narrative synthesis. Information Processing & Management. 2021;58(6):102713.

209. Raihan MMH. Mental health consequences of COVID-19 pandemic on adult population: a systematic review. Mental Health Review Journal. 2021;26(1):42-54.

210. Porter B, Zile A, Peryer G, Farquhar M, Sanderson K. The impact of providing end-of-life care during a pandemic on the mental health and wellbeing of health and social care staff: Systematic review and meta-synthesis. Social Science & Medicine. 2021;287:114397.

211. Premraj L, Kannapadi NV, Briggs J, Seal SM, Battaglini D, Fanning J, et al. Mid and long-term neurological and neuropsychiatric manifestations of post-COVID-19 syndrome: A meta-analysis. Journal of the Neurological Sciences. 2022;434.

212. Purnama A, Susaldi S, Zahro Mukhlida H, Hasro Maulida H, Purwati NH. Mental Health in Health Students during Coronavirus Disease-19: Systematic Review. Open Access Macedonian Journal of Medical Sciences. 2021;9(F):205-10.

213. Qiu D, Li Y, Li L, He J, Ouyang F, Xiao S. Prevalence of post-traumatic stress symptoms among people influenced by coronavirus disease 2019 outbreak: A meta-analysis. European Psychiatry: the Journal of the Association of European Psychiatrists. 2021;64(1):e30.

214. Qiu D, Li Y, Li L, He J, Ouyang F, Xiao S. Infectious Disease Outbreak and Post-Traumatic Stress Symptoms: A Systematic Review and Meta-Analysis. Frontiers in Psychology. 2021;12.

215. Racine N, McArthur BA, Cooke JE, Eirich R, Zhu J, Madigan S. Global Prevalence of Depressive and Anxiety Symptoms in Children and Adolescents During COVID-19: A Meta-analysis. JAMA Pediatrics. 2021;175(11):1142-50.

216. Raoofi S, Pashazadeh Kan F, Rafiei S, Khani S, Hosseinifard H, Tajik F, et al. Anxiety during the COVID-19 pandemic in hospital staff: systematic review plus meta-analysis. BMJ Supportive &amp; Palliative Care. 2021:bmjspcare-2021-003125.

217. Rawat D, Dixit V, Gulati S, Gulati A. Impact of COVID-19 outbreak on lifestyle behaviour: A review of studies published in India. Diabetes and Metabolic Syndrome: Clinical Research and Reviews. 2021;15(1):331-6.

218. Ren X, Huang W, Pan H, Huang T, Wang X, Ma Y. Mental Health During the Covid-19 Outbreak in China: a Meta-Analysis. Psychiatric Quarterly. 2020;91(4):1033-45.

219. Hachesu VR, Naderyan Fe’li S, Maajani K, Hokmabadi R, Golbabaei F. Prevalence of Anxiety and Depression in Iranian Health Care Workers during the COVID-19 Pandemic: A Systematic Review and Meta-Analysis. Journal of Health and Safety at Work. 2022;12(1):123-40.

220. Rezaei S, Hoseinipalangi Z, Rafiei S, Dolati Y, Hosseinifard H, Asl MT, et al. The global Prevalence of Depression among Health Workers during the COVID-19 pandemic: a Systematic review and Meta-analysis. Journal of Affective Disorders Reports. 2022;8:100326.

221. Roberts A, Rogers J, Mason R, Siriwardena AN, Hogue T, Whitley GA, et al. Alcohol and other substance use during the COVID-19 pandemic: A systematic review. Drug and Alcohol Dependence. 2021;229:109150.

222. Robinson E, Sutin AR, Daly M, Jones A. A systematic review and meta-analysis of longitudinal cohort studies comparing mental health before versus during the COVID-19 pandemic in 2020. Journal of Affective Disorders. 2022;296:567-76.

223. Rocha YM, de Moura GA, Desidério GA, de Oliveira CH, Lourenço FD, de Figueiredo Nicolete LD. The impact of fake news on social media and its influence on health during the COVID-19 pandemic: a systematic review. Journal of Public Health. 2021.

224. Rodríguez-Fernández P, González-Santos J, Santamaría-Peláez M, Soto-Cámara R, Sánchez-González E, González-Bernal JJ. Psychological Effects of Home Confinement and Social Distancing Derived from COVID-19 in the General Population—A Systematic Review. International Journal of Environmental Research and Public Health. 2021;18(12):6528.

225. Rogers JP, Chesney E, Oliver D, Pollak TA, McGuire P, Fusar-Poli P, et al. Psychiatric and neuropsychiatric presentations associated with severe coronavirus infections: a systematic review and meta-analysis with comparison to the COVID-19 pandemic. The Lancet Psychiatry. 2020;7(7):611-27.

226. Rogers JP, Watson CJ, Badenoch J, Cross B, Butler M, Song J, et al. Neurology and neuropsychiatry of COVID-19: a systematic review and meta-analysis of the early literature reveals frequent CNS manifestations and key emerging narratives. Journal of Neurology, Neurosurgery &amp; Psychiatry. 2021;92(9):932-41.

227. Ruksakulpiwat S, Zhou W, Phianhasin L, Benjasirisan C, Fan Y, Su T, et al. The experience of caregivers of chronically ill patients during the COVID-19: A Systematic Review. Chronic Illness. 2021;18(3):488-502.

228. Runacres A, Mackintosh KA, Knight RL, Sheeran L, Thatcher R, Shelley J, et al. Impact of the COVID-19 Pandemic on Sedentary Time and Behaviour in Children and Adults: A Systematic Review and Meta-Analysis. International Journal of Environmental Research and Public Health. 2021;18(21):11286.

229. Russo G, Jesus TS, Deane K, Osman AY, McCoy D. Epidemics, Lockdown Measures and Vulnerable Populations: A Mixed-Methods Systematic Review of the Evidence of Impacts on Mother and Child Health in Low- and Lower-Middle-Income Countries. International Journal of Health Policy and Management. 2021:-.

230. Saeed H, Eslami A, Nassif NT, Simpson AM, Lal S. Anxiety Linked to COVID-19: A Systematic Review Comparing Anxiety Rates in Different Populations. International Journal of Environmental Research and Public Health. 2022;19(4):2189.

231. Safi-Keykaleh M, Aliakbari F, Safarpour H, Safari M, Tahernejad A, Sheikhbardsiri H, et al. Prevalence of postpartum depression in women amid the COVID-19 pandemic: A systematic review and meta-analysis. International Journal of Gynecology & Obstetrics. 2022;157(2):240-7.

232. Salamanna F, Veronesi F, Martini L, Landini MP, Fini M. Post-COVID-19 Syndrome: The Persistent Symptoms at the Post-viral Stage of the Disease. A Systematic Review of the Current Data. Frontiers in Medicine. 2021;8.

233. Salari N, Hosseinian-Far A, Jalali R, Vaisi-Raygani A, Mohammadi M, Rasoulpoor S, et al. Prevalence of stress, anxiety, depression among the general population during the COVID-19 pandemic: A systematic review and meta-analysis. Globalization and Health. 2020;16(1).

234. Salari N, Khazaie H, Hosseinian-Far A, Khaledi-Paveh B, Kazeminia M, Mohammadi M, et al. The prevalence of stress, anxiety and depression within front-line healthcare workers caring for COVID-19 patients: a systematic review and meta-regression. Human Resources for Health. 2020;18(1).

235. Salazar de Pablo G, Vaquerizo-Serrano J, Catalan A, Arango C, Moreno C, Ferre F, et al. Impact of coronavirus syndromes on physical and mental health of health care workers: Systematic review and meta-analysis. Journal of Affective Disorders. 2020;275:48-57.

236. Salehi M, Amanat M, Mohammadi M, Salmanian M, Rezaei N, Saghazadeh A, et al. The prevalence of post-traumatic stress disorder related symptoms in Coronavirus outbreaks: A systematic-review and meta-analysis. Journal of Affective Disorders. 2021;282:527-38.

237. Samji H, Wu J, Ladak A, Vossen C, Stewart E, Dove N, et al. Review: Mental health impacts of the COVID-19 pandemic on children and youth – a systematic review. Child and Adolescent Mental Health. 2022;27(2):173-89.

238. Sanghera J, Pattani N, Hashmi Y, Varley KF, Cheruvu MS, Bradley A, et al. The impact of SARS-CoV-2 on the mental health of healthcare workers in a hospital setting-A Systematic Review. Journal of Occupational Health. 2020;62(1):e12175.

239. Santabarbara J, Lasheras I, Lipnicki DM, Bueno-Notivol J, Perez-Moreno M, Lopez-Anton R, et al. Prevalence of anxiety in the COVID-19 pandemic: An updated meta-analysis of community-based studies. Progress in Neuro-Psychopharmacology & Biological Psychiatry. 2020;109:110207.

240. Santabárbara J, Ozamiz-Etxebarria N, Idoiaga N, Olaya B, Bueno-Novitol J. Meta-Analysis of Prevalence of Depression in Dental Students during COVID-19 Pandemic. Medicina. 2021;57(11):1278.

241. Santabarbara J, Bueno-Notivol J, Lipnicki DM, Olaya B, Perez-Moreno M, Gracia-Garcia P, et al. Prevalence of anxiety in health care professionals during the COVID-19 pandemic: A rapid systematic review (on published articles in Medline) with meta-analysis. Progress in Neuro-Psychopharmacology & Biological Psychiatry. 2021:110244.

242. Santomauro DF, Mantilla Herrera AM, Shadid J, Zheng P, Ashbaugh C, Pigott DM, et al. Global prevalence and burden of depressive and anxiety disorders in 204 countries and territories in 2020 due to the COVID-19 pandemic. The Lancet. 2021;398(10312):1700-12.

243. Saragih ID, Tonapa SI, Saragih IS, Advani S, Batubara SO, Suarilah I, et al. Global prevalence of mental health problems among healthcare workers during the Covid-19 pandemic: A systematic review and meta-analysis. International Journal of Nursing Studies. 2021;121:104002.

244. Schneider J, Pegram G, Gibson B, Talamonti D, Tinoco A, Craddock N, et al. A mixed-studies systematic review of the experiences of body image, disordered eating, and eating disorders during the COVID-19 pandemic. International Journal of Eating Disorders.n/a(n/a).

245. Schou TM, Joca S, Wegener G, Bay-Richter C. Psychiatric and neuropsychiatric sequelae of COVID-19 – A systematic review. Brain, Behavior, and Immunity. 2021;97:328-48.

246. Schubert M, Ludwig J, Freiberg A, Hahne TM, Romero Starke K, Girbig M, et al. Stigmatization from Work-Related COVID-19 Exposure: A Systematic Review with Meta-Analysis. International Journal of Environmental Research and Public Health. 2021;18(12):6183.

247. Scortegagna SA, LimaI EdSd, Pasian SR, AmparoIII DMd. Mental health in health professionals facing Covid-19: a systematic review. Psicologia: teoria e prática. 2021;23(1):1-23.

248. Serrano-Ripoll MJ, Meneses-Echavez JF, Ricci-Cabello I, Fraile-Navarro D, Fiol-deRoque MA, Pastor-Moreno G, et al. Impact of viral epidemic outbreaks on mental health of healthcare workers: a rapid systematic review and meta-analysis. Journal of Affective Disorders. 2020;277:347-57.

249. Seyed ASA, Karimi A, Shobeiri P, Nowroozi A, Mehraeen E, Afsahi AM, et al. Psychological symptoms of COVID-19 epidemic: a systematic review of current evidence. Psihologija. 2021;54(2):173-92.

250. Shankar A, Yu BE, Malvankar-Mehta M. The psychological impact of COVID-19 on socially isolated individuals – a systematic review. Mental Health Review Journal. 2021;26(3):247-57.

251. Sheraton M, Deo N, Dutt T, Surani S, Hall-Flavin D, Kashyap R. Psychological effects of the COVID 19 pandemic on healthcare workers globally: A systematic review. Psychiatry Research. 2020;292:113360.

252. Shorey SY, Ng ED, Chee CYI. Anxiety and depressive symptoms of women in the perinatal period during the COVID-19 pandemic: A systematic review and meta-analysis. Scandinavian Journal of Public Health. 2021:14034948211011793.

253. Shukla J, Manohar Singh R. Psychological Health amidst COVID-19: A Review of existing literature in the Indian Context. Clinical Epidemiology and Global Health. 2021;11 (no pagination).

254. Sideli L, Lo Coco G, Bonfanti RC, Borsarini B, Fortunato L, Sechi C, et al. Effects of COVID-19 lockdown on eating disorders and obesity: A systematic review and meta-analysis. European Eating Disorders Review. 2021;29(6):826-41.

255. Silva DFO, Cobucci RN, Lima SCVC, de Andrade FB. Prevalence of anxiety, depression, and stress among teachers during the COVID-19 pandemic: A PRISMA-compliant systematic review. Medicine. 2021;100(44).

256. Simonetti A, Pais C, Jones M, Cipriani MC, Janiri D, Monti L, et al. Neuropsychiatric Symptoms in Elderly With Dementia During COVID-19 Pandemic: Definition, Treatment, and Future Directions. Frontiers in Psychiatry. 2020;11.

257. Simsir Z, Koc H, Seki T, Griffiths MD. The relationship between fear of COVID-19 and mental health problems: A meta-analysis. Death studies. 2021:1-9.

258. Singh RK, Bajpai R, Kaswan P. COVID-19 pandemic and psychological wellbeing among health care workers and general population: A systematic-review and meta-analysis of the current evidence from India. Clinical Epidemiology & Global Health. 2021;11:100737.

259. Ślusarska B, Nowicki GJ, Niedorys-Karczmarczyk B, Chrzan-Rodak A. Prevalence of Depression and Anxiety in Nurses during the First Eleven Months of the COVID-19 Pandemic: A Systematic Review and Meta-Analysis. International Journal of Environmental Research and Public Health. 2022;19(3):1154.

260. Smith CM, Gilbert EB, Riordan PA, Helmke N, von Isenburg M, Kincaid BR, et al. COVID-19-associated psychosis: A systematic review of case reports. General Hospital Psychiatry. 2021;73:84-100.

261. Soklaridis S, Lin E, Lalani Y, Rodak T, Sockalingam S. Mental health interventions and supports during COVID- 19 and other medical pandemics: A rapid systematic review of the evidence. General Hospital Psychiatry. 2020;66:133-46.

262. Solehati T, Kosasih CE, Hermayanti Y, Mediani HS. The Psychological and Sleep-Related Impact of Coronavirus Disease 2019 (COVID-19): A Systematic Review. Kesmas: Jurnal Kesehatan Masyarakat Nasional (National Public Health Journal). 2021.

263. Soltani S, Tabibzadeh A, Zakeri A, Zakeri AM, Latifi T, Shabani M, et al. COVID-19 associated central nervous system manifestations, mental and neurological symptoms: a systematic review and meta-analysis. Reviews in the Neurosciences. 2021;32(3):351-61.

264. Soysal P, Smith L, Trott M, Alexopoulos P, Barbagallo M, Tan SG, et al. The Effects of COVID-19 lockdown on neuropsychiatric symptoms in patients with dementia or mild cognitive impairment: A systematic review and meta-analysis. Psychogeriatrics. 2022;22(3):402-12.

265. Sterina E, Hermida AP, Gerberi DJ, Lapid MI. Emotional Resilience of Older Adults during COVID-19: A Systematic Review of Studies of Stress and Well-Being. Clinical Gerontologist. 2022;45(1):4-19.

266. Strasser MA, Sumner PJ, Meyer D. COVID-19 news consumption and distress in young people: A systematic review. Journal of Affective Disorders. 2022;300:481-91.

267. Suárez-González A, Rajagopalan J, Livingston G, Alladi S. The effect of COVID-19 isolation measures on the cognition and mental health of people living with dementia: A rapid systematic review of one year of quantitative evidence. eClinicalMedicine. 2021;39.

268. Sun F, Zhu J, Tao H, Ma Y, Jin W. A systematic review involving 11,187 participants evaluating the impact of COVID-19 on anxiety and depression in pregnant women. Journal of Psychosomatic Obstetrics and Gynecology. 2020.

269. Sun P, Wang M, Song T, Wu Y, Luo J, Chen L, et al. The Psychological Impact of COVID-19 Pandemic on Health Care Workers: A Systematic Review and Meta-Analysis. Frontiers in Psychology. 2021;12.

270. Tashakori-Miyanroudi M, Souresrafil A, Hashemi P, Jafar Ehsanzadeh S, Farrahizadeh M, Behroozi Z. Prevalence of depression, anxiety, and psychological distress in patients with epilepsy during COVID-19: A systematic review. Epilepsy & Behavior. 2021;125.

271. Thakur B, Pathak M. Burden of predominant psychological reactions among the healthcare workers and general population during COVID-19 pandemic phase: A systematic review and meta-analysis. Indian Journal of Community Medicine: Official Publication of Indian Association of Preventive & Social Medicine. 2021;46(4):600.

272. Thatrimontrichai A, Weber DJ, Apisarnthanarak A. Mental health among healthcare personnel during COVID-19 in Asia: A systematic review. Journal of the Formosan Medical Association. 2021;01:01.

273. Tibubos AN, Otten D, Ernst M, Beutel ME. A Systematic Review on Sex- and Gender-Sensitive Research in Public Mental Health During the First Wave of the COVID-19 Crisis. Frontiers in Psychiatry. 2021;12.

274. Tomfohr-Madsen LM, Racine N, Giesbrecht GF, Lebel C, Madigan S. Depression and anxiety in pregnancy during COVID-19: A rapid review and meta-analysis. Psychiatry Research. 2021;300:113912.

275. Troglio da Silva FC, Neto MLR. Psychiatric disorders in health professionals during the COVID-19 pandemic: A systematic review with meta-analysis. Journal of Psychiatric Research. 2021;140:474-87.

276. Uphoff EP, Lombardo C, Johnston G, Weeks L, Rodgers M, Dawson S, et al. Mental health among healthcare workers and other vulnerable groups during the COVID-19 pandemic and other coronavirus outbreaks: A rapid systematic review. PLOS ONE. 2021;16(8):e0254821.

277. Usmani S, Greca E, Javed S, Sharath M, Sarfraz Z, Sarfraz A, et al. Risk Factors for Postpartum Depression During COVID-19 Pandemic: A Systematic Literature Review. Journal of Primary Care & Community Health. 2021;12:21501327211059348.

278. van Reekum EA, Rosic T, Sergeant A, Sanger N, Rodrigues M, Rebinsky R, et al. Delirium and other neuropsychiatric manifestations of COVID-19 infection in people with preexisting psychiatric disorders: a systematic review. Journal of Medical Case Reports. 2021;15(1):586.

279. Vanderlind WM, Rabinovitz BB, Miao IY, Oberlin LE, Bueno-Castellano C, Fridman C, et al. A systematic review of neuropsychological and psychiatric sequalae of COVID-19: implications for treatment. Current Opinion in Psychiatry. 2021;34(4).

280. Varghese A, George G, Kondaguli SV, Naser AY, Khakha DC, Chatterji R. Decline in the mental health of nurses across the globe during COVID-19: A systematic review and meta-analysis. Journal of Global Health. 2021;11:05009.

281. Vescovi G, Riter HdS, Azevedo EC, Pedrotti BG, Frizzo GB. Parenting, mental health, and Covid-19: a rapid systematic review. Psicologia: teoria e prática São Paulo Vol 23, n 1 (2021), p 1-28. 2021.

282. Vindegaard N, Benros ME. COVID-19 pandemic and mental health consequences: Systematic review of the current evidence. Brain, Behavior, & Immunity. 2020;89:531-42.

283. Violant-Holz V, Gallego-Jimenez MG, Gonzalez-Gonzalez CS, Munoz-Violant S, Rodriguez MJ, Sansano-Nadal O, et al. Psychological Health and Physical Activity Levels during the COVID-19 Pandemic: A Systematic Review. International Journal of Environmental Research & Public Health [Electronic Resource]. 2020;17(24):15.

284. Vizheh M, Qorbani M, Arzaghi SM, Muhidin S, Javanmard Z, Esmaeili M. The mental health of healthcare workers in the COVID-19 pandemic: A systematic review. Journal of Diabetes & Matabolic Disorders. 2020:1-12.

285. Wang C, Wen W, Zhang H, Ni J, Jiang J, Cheng Y, et al. Anxiety, depression, and stress prevalence among college students during the COVID-19 pandemic: A systematic review and meta-analysis. Journal of American College Health. 2021:1-8.

286. Wang F, Zhang L, Ding L, Wang L, Deng Y. Fear of COVID-19 Among College Students: A Systematic Review and Meta-Analysis. Front Public Health. 2022;10.

287. Wang Y, Kala MP, Jafar TH. Factors associated with psychological distress during the coronavirus disease 2019 (COVID- 19) pandemic on the predominantly general population: A systematic review and metaanalysis. PLoS ONE. 2020;15(12 December).

288. Wang Z, Wang D. The influence and enlightenment of five public health emergencies on public psychology since new century: A systematic review. International Journal of Social Psychiatry. 2021:207640211002222.

289. Whear R, Abbott RA, Bethel A, Richards DA, Garside R, Cockcroft E, et al. Impact of COVID-19 and other infectious conditions requiring isolation on the provision of and adaptations to fundamental nursing care in hospital in terms of overall patient experience, care quality, functional ability, and treatment outcomes: systematic review. Journal of Advanced Nursing. 2022;78(1):78-108.

290. Willi S, Luthold R, Hunt A, Hanggi NV, Sejdiu D, Scaff C, et al. COVID-19 sequelae in adults aged less than 50 years: A systematic review. Travel Medicine & Infectious Disease. 2021;40:101995.

291. Wolf S, Seiffer B, Zeibig JM, Welkerling J, Brokmeier L, Atrott B, et al. Is Physical Activity Associated with Less Depression and Anxiety During the COVID-19 Pandemic? A Rapid Systematic Review. Sports Medicine. 2021;22:22.

292. Wu L, Wu Y, Xiong H, Mei B, You T. Persistence of Symptoms After Discharge of Patients Hospitalized Due to COVID-19. Frontiers in Medicine. 2021;8.

293. Wu T, Jia X, Shi H, Niu J, Yin X, Xie J, et al. Prevalence of mental health problems during the COVID-19 pandemic: A systematic review and meta-analysis. Journal of Affective Disorders. 2021;281:91-8.

294. Wu Y, Liu W, Liu A, Lin-Schilstra L, Lyu P. International Students' Mental Health Care in China: A Systematic Review. Healthcare. 2021;9(12):1634.

295. Xiong J, Lipsitz O, Nasri F, Lui LM, Gill H, Phan L, et al. Impact of COVID-19 pandemic on mental health in the general population: A systematic review. Journal of Affective Disorders. 2020;277:55-64.

296. Xiong N, Fritzsche K, Pan Y, Löhlein J, Leonhart R. The psychological impact of COVID-19 on Chinese healthcare workers: a systematic review and meta-analysis. Social Psychiatry and Psychiatric Epidemiology. 2022;57(8):1515-29.

297. Xu H, Stjernswärd S, Glasdam S. Psychosocial experiences of frontline nurses working in hospital-based settings during the COVID-19 pandemic - A qualitative systematic review. International Journal of Nursing Studies Advances. 2021;3:100037.

298. Yaghoubi M, Salimi M, Meskarpour-Amiri M. Systematic review of productivity loss among healthcare workers due to Covid-19. The International Journal of Health Planning and Management. 2022;37(1):94-111.

299. Yan H, Ding Y, Guo W. Mental Health of Pregnant and Postpartum Women During the Coronavirus Disease 2019 Pandemic: A Systematic Review and Meta-Analysis. Frontiers in Psychology. 2020;11.

300. Yan H, Ding Y, Guo W. Mental health of medical staff during the coronavirus disease 2019 (COVID-19) pandemic: A systematic review and meta-analysis. Psychosomatic medicine. 2021;10.

301. Yan Y, Du X, Lai L, Ren Z, Li H. Prevalence of depressive and anxiety symptoms among Chinese older adults during the COVID-19 pandemic: A systematic review and meta-analysis. Journal of Geriatric Psychiatry and Neurology. 2022;35(2):182-95.

302. Yılmaz B, Azak M, Şahin N. Mental health of parents of children with autism spectrum disorder during COVID-19 pandemic: A systematic review. World Journal of Psychiatry. 2021;11(7):388.

303. Yuan K, Gong Y-M, Liu L, Sun Y-K, Tian S-S, Wang Y-J, et al. Prevalence of posttraumatic stress disorder after infectious disease pandemics in the twenty-first century, including covid-19: A meta-analysis and systematic review. Molecular Psychiatry. 2021:No Pagination Specified.

304. Yunitri N, Chu H, Kang XL, Jen H-J, Pien L-C, Tsai H-T, et al. Global prevalence and associated risk factors of posttraumatic stress disorder during COVID-19 pandemic: A meta-analysis. International Journal of Nursing Studies. 2022;126:104136.

305. Zarghami A, Hussain MA, Campbell JA, Ezegbe C, van der Mei I, Taylor BV, et al. Psychological impacts of COVID-19 pandemic on individuals living with multiple sclerosis: A rapid systematic review. Multiple Sclerosis and Related Disorders. 2022;59.

306. Zhang H, Xie F, Yang B, Zhao F, Wang C, Chen X. Psychological experience of COVID-19 patients: A systematic review and qualitative meta-synthesis. American Journal of Infection Control. 2022;50(7):809-19.

307. Zhang L, Pan R, Cai Y, Pan J. The Prevalence of Post-Traumatic Stress Disorder in the General Population during the COVID-19 Pandemic: A Systematic Review and Single-Arm Meta-Analysis. Psychiatry Investigation. 2021;30:30.

308. Zhang SX, Chen J. Scientific evidence on mental health in key regions under the COVID-19 pandemic – meta-analytical evidence from Africa, Asia, China, Eastern Europe, Latin America, South Asia, Southeast Asia, and Spain. European Journal of Psychotraumatology. 2021;12(1):2001192.

309. Zhang SX, Chen RZ, Xu W, Yin A, Dong RK, Chen BZ, et al. A Systematic Review and Meta-Analysis of Symptoms of Anxiety, Depression, and Insomnia in Spain in the COVID-19 Crisis. International Journal of Environmental Research and Public Health. 2022;19(2):1018.

310. Zhang Y, Bao X, Yan J, Miao H, Guo C. Anxiety and Depression in Chinese Students During the COVID-19 Pandemic: A Meta-Analysis. Front Public Health. 2021;9.

311. Zhao YJ, Jin Y, Rao WW, Li W, Zhao N, Cheung T, et al. The prevalence of psychiatric comorbidities during the SARS and COVID-19 epidemics: a systematic review and meta-analysis of observational studies. Journal of Affective Disorders. 2021;287:145-57.

312. Zhou Y, Sun Z, Wang Y, Xing C, Sun L, Shang Z, et al. The prevalence of PTSS under the influence of public health emergencies in last two decades: A systematic review and meta-analysis. Clinical Psychology Review. 2021;83.

313. Zhu J, Racine N, Xie EB, Park J, Watt J, Eirich R, et al. Post-secondary Student Mental Health During COVID-19: A Meta-Analysis. Frontiers in Psychiatry. 2021;12.

314. Deng J, Zhou F, Hou W, Silver Z, Wong CY, Chang O, et al. The prevalence of depression, anxiety, and sleep disturbances in COVID-19 patients: a meta-analysis. Annals of the New York Academy of Sciences. 2021;1486(1):90-111.

315. Octavius GS, Silviani FR, Lesmandjaja A, Angelina, Juliansen A. Impact of COVID-19 on adolescents’ mental health: a systematic review. Middle East Current Psychiatry. 2020;27(1).

316. Hope C, Reilly JJ, Griffiths G, Lund J, Humes D. The impact of COVID-19 on surgical training: a systematic review. Techniques in Coloproctology. 2021;28:28.

317. Schneider J, Talamonti D, Gibson B, Forshaw M. Factors mediating the psychological well-being of healthcare workers responding to global pandemics: A systematic review. Journal of Health Psychology. 2021:13591053211012759.

318. Chmielewska B, Barratt I, Townsend R, Kalafat E, van der Meulen J, Gurol-Urganci I, et al. Effects of the COVID-19 pandemic on maternal and perinatal outcomes: a systematic review and meta-analysis. The Lancet Global Health. 2021;31:31.

319. Sirois FM, Owens J. Factors Associated With Psychological Distress in Health-Care Workers During an Infectious Disease Outbreak: A Rapid Systematic Review of the Evidence. Frontiers in psychiatry Frontiers Research Foundation. 2020;11:589545.

320. John A, Okolie C, Eyles E, Webb RT, Schmidt L, McGuiness LA, et al. The impact of the COVID-19 pandemic on self-harm and suicidal behaviour: a living systematic review. F1000Research. 2020;9:1097.

321. Eyles E, Moran P, Okolie C, Dekel D, Macleod-Hall C, Webb RT, et al. Systematic review of the impact of the COVID-19 pandemic on suicidal behaviour amongst health and social care workers across the world. Journal of Affective Disorders Reports. 2021;6:100271.

322. Lee Y, Lui LM, Chen-Li D, Liao Y, Mansur RB, Brietzke E, et al. Government response moderates the mental health impact of COVID-19: a systematic review and meta-analysis of depression outcomes across countries. Journal of affective disorders. 2021;290:364-77.

323. Li Y, Wang A, Wu Y, Han N, Huang H. Impact of the COVID-19 Pandemic on the Mental Health of College Students: A Systematic Review and Meta-Analysis. Frontiers in Psychology. 2021;12.

324. Muehlschlegel PA, Parkinson EA, Chan RY, Arden MA, Armitage CJ. Learning from previous lockdown measures and minimising harmful biopsychosocial consequences as they end: A systematic review. Journal of global health. 2021;11.

325. Nochaiwong S, Ruengorn C, Thavorn K, Hutton B, Awiphan R, Phosuya C, et al. Global prevalence of mental health issues among the general population during the coronavirus disease-2019 pandemic: a systematic review and meta-analysis. Scientific Reports. 2021;11(1):10173.

326. Renaud-Charest O, Lui LMW, Eskander S, Ceban F, Ho R, Di Vincenzo JD, et al. Onset and frequency of depression in post-COVID-19 syndrome: A systematic review. Journal of Psychiatric Research. 2021;144:129-37.

327. Schmidt RA, Genois R, Jin J, Vigo D, Rehm J, Rush B. The early impact of COVID-19 on the incidence, prevalence, and severity of alcohol use and other drugs: A systematic review. Drug and Alcohol Dependence. 2021;228:109065.

328. Soto-Cámara R, García-Santa-Basilia N, Onrubia-Baticón H, Cárdaba-García RM, Jiménez-Alegre JJ, Reques-Marugán AM, et al. Psychological Impact of the COVID-19 Pandemic on Out-of-Hospital Health Professionals: A Living Systematic Review. Journal of Clinical Medicine. 2021;10(23):5578.

329. Zhang H, Li W, Li H, Zhang C, Luo J, Zhu Y, et al. Prevalence and dynamic features of psychological issues among Chinese healthcare workers during the COVID-19 pandemic: a systematic review and cumulative meta-analysis. General Psychiatry. 2021;34(3):e100344.

330. Alimoradi Z, Ohayon MM, Griffiths MD, Lin CY, Pakpour AH. Fear of COVID-19 and its association with mental health-related factors: systematic review and meta-analysis. BJPsych Open. 2022;8(2):e73.

331. Jefferson L, Golder S, Heathcote C, Avila AC, Dale V, Essex H, et al. GP wellbeing during the COVID-19 pandemic: a systematic review. British Journal of General Practice. 2022;72(718):e325-e33.

332. Racine N, Eirich R, Cooke J, Zhu J, Pador P, Dunnewold N, et al. When the Bough Breaks: A systematic review and meta-analysis of mental health symptoms in mothers of young children during the COVID-19 pandemic. Infant Mental Health Journal. 2022;43(1):36-54.

333. Veazie S, Lafavor B, Vela K, Young S, Sayer NA, Carlson KF, et al. Mental health outcomes of adults hospitalized for COVID-19: A systematic review. Journal of Affective Disorders Reports. 2022;8:100312.

334. Viner R, Russell S, Saulle R, Croker H, Stansfield C, Packer J, et al. School Closures During Social Lockdown and Mental Health, Health Behaviors, and Well-being Among Children and Adolescents During the First COVID-19 Wave: A Systematic Review. JAMA Pediatrics. 2022;176(4):400-9.

335. Wan Mohd Yunus WMA, Kauhanen L, Sourander A, Brown JSL, Peltonen K, Mishina K, et al. Registered psychiatric service use, self-harm and suicides of children and young people aged 0–24 before and during the COVID-19 pandemic: a systematic review. Child and Adolescent Psychiatry and Mental Health. 2022;16(1):15.

336. Liu C, Pan W, Li L, Li B, Ren Y, Ma X. Prevalence of depression, anxiety, and insomnia symptoms among patients with COVID-19: A meta-analysis of quality effects model. Journal of Psychosomatic Research. 2021;147:110516.

337. Kilian C, O'Donnell A, Potapova N, López-Pelayo H, Schulte B, Miquel L, et al. Changes in alcohol use during the COVID-19 pandemic in Europe: A meta-analysis of observational studies. Drug and Alcohol Review. 2022;41(4):918-31.

338. Zhang SX, Miller SO, Xu W, Yin A, Chen BZ, Delios A, et al. Meta-analytic evidence of depression and anxiety in Eastern Europe during the COVID-19 pandemic. European Journal of Psychotraumatology. 2022;13(1):2000132.
